# A convergent synthetic approach to the tetracyclic core framework of khayanolide-type limonoids

**DOI:** 10.3762/bjoc.21.75

**Published:** 2025-05-12

**Authors:** Zhiyang Zhang, Jialei Hu, Hanfeng Ding, Li Zhang, Peirong Rao

**Affiliations:** 1 School of Chemistry and Chemical Engineering, Zhejiang Sci-Tech University, Hangzhou 310018, China; 2 Department of Chemistry, Zhejiang University, Hangzhou 310058, China; 3 Hangzhou DAC Biotechnology Co., Ltd 369 Qiaoxin Road, Qiantang District, Hangzhou 310018, Zhejiang, China

**Keywords:** enantioselective synthesis, interrupted Nazarov cyclization, khayanolide-type limonoids, tetracyclic framework

## Abstract

A convergent approach for the enantioselective construction of an advanced intermediate containing the [5,5,6,6]-tetracyclic core framework of the khayanolide-type limonoids was described. The strategy features an acylative kinetic resolution of the benzylic alcohol, a 1,2-Grignard addition and an AcOH-interrupted Nazarov cyclization.

## Introduction

**Figure 1:**
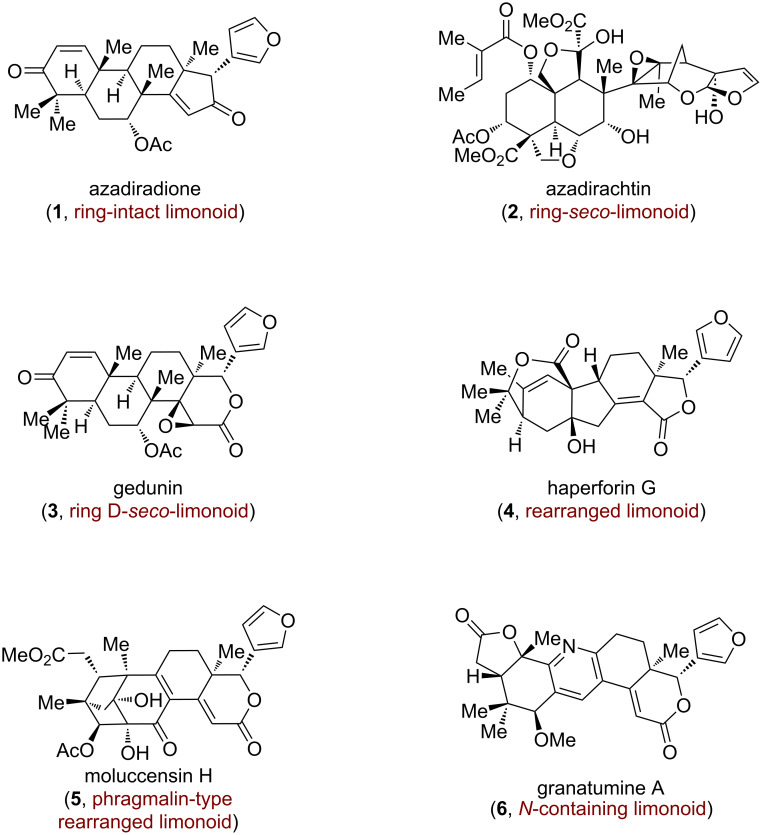
Representative limonoid triterpenes.

Limonoids, a class of tetranortriterpenoids derived biosynthetically from oxidative truncation of apotirucallane or apoeuphane precursors coupled with subsequent β-furan annulation [1–6], constitute an architecturally sophisticated family of natural products. Based on their specific skeletal rearrangements, limonoids can be systematically categorized into four subfamilies: ring intact limonoids, ring-seco limonoids, rearranged limonoids and N-containing limonoids (Figure 1). Moreover, these molecules exhibit a remarkable pharmacological portfolio encompassing anticancer, antimicrobial, anti-inflammatory, and insect antifeedant activities [7–9], positioning them as compelling targets for both therapeutic development and agrochemical innovation. Furthermore, limonoids are also renowned for their extraordinary structural complexity. For instance, the phragmalin-type limonoids represent a significant category of highly oxygenated rearranged limonoids, which contain a distinctive octahydro-1*H*-2,4-methanoindene cage. The fascinating architectures and remarkable biological profiles of these compounds have attracted widespread attention from the synthetic community. In 1989, Corey and Hahl made a seminal contribution [10] to the field by completing the total synthesis of azadiradione (**1**). Following this, Ley and his team successfully synthesized azadirachtin (**2**) in 2007, a limonoid extensively utilized in organic agriculture [11]. In recent years, remarkable progress has been made on the total syntheses of various limonoids, with notable contributions from researchers such as Williams [12], Yamashita [13], Hao/Yang/Shen [14], Gong/Hao/Yang [15], Newhouse [16–19], Yang/Chen [20], Renata [21], Qin/Yu [22], Ma [23], Watanabe [24], and Li [25]. Their groundbreaking work has provided valuable insights and inspired further research in synthetic chemistry involving limonoids.

**Scheme 1:**
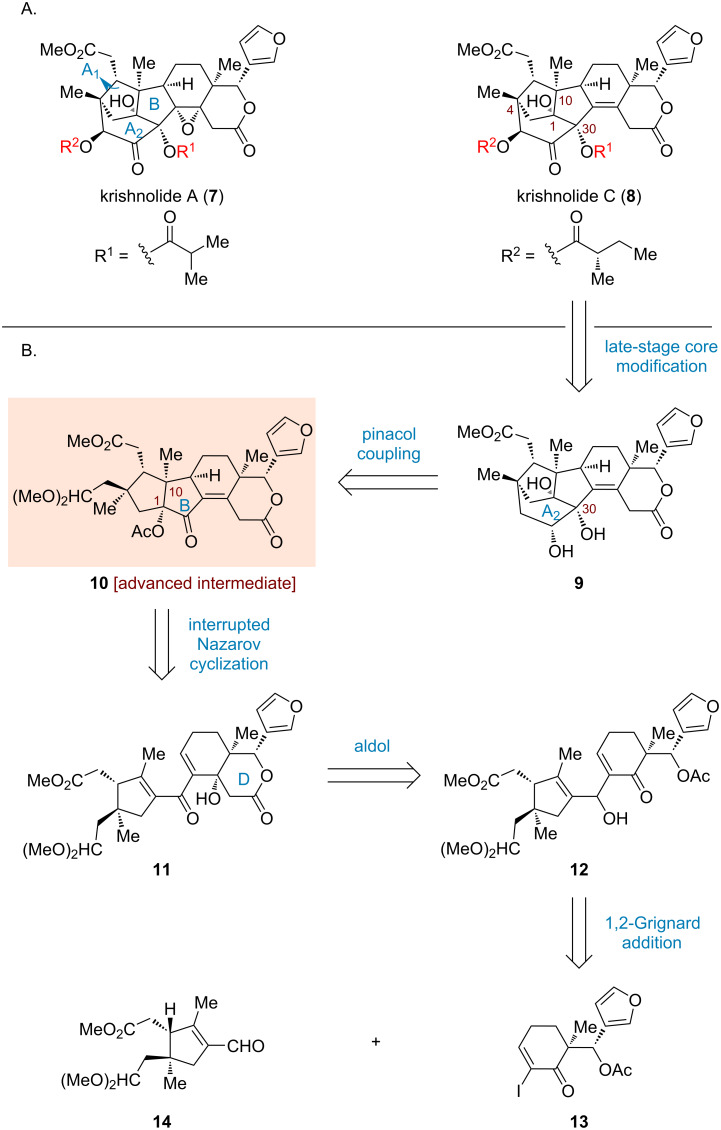
Structures and retrosynthetic analysis of krishnolides A (**7**) and C (**8**).

Krishnolides A and C (**7** and **8**, respectively; Scheme 1A) were identified by Wu and co-workers from the seeds of a Krishna mangrove *Xylocarpus moluccensis* [26]. These two molecules belong to khayanolides, a class of rearranged phragmalin limonoids characterized by a structurally intricate tricyclo[4.2.1^10,30^.1^1,4^]decane ring system. Additionally, krishnolides A and C contain 9–11 stereogenic centers and exhibit diverse oxidation patterns. Their relative and absolute configurations were determined through NMR, HRESIMS and ECD experiments, as well as single crystal X-ray diffraction analysis. A preliminary investigation revealed that krishnolide A (**7**) exhibited unique anti-human immunodeficiency virus (HIV) activity, representing the first report of anti-HIV activity in khayanolide-type limonoids. However, the highly oxygenated and polycyclic scaffolds pose substantial challenges toward their total synthesis. Two synthetic studies were disclosed successively by Sarpong [27] and Jirgensons [28], both focusing on the construction of the unique methanoindene cage structure (A_1_A_2_B ring system). Building upon our previous syntheses of phragmalin-type limonoids [29], we herein disclose a convergent approach leveraging an AcOH-interrupted Nazarov cyclization to establish the [5,5,6,6]-tetracyclic scaffold with precise stereochemical fidelity.

## Results and Discussion

Our retrosynthetic analysis toward krishnolides A (**7**) and C (**8**) is delineated in Scheme 1B. We hypothesized that these two molecules could be synthesized from diol **9** through a late-stage modification involving adjustment of the oxidation state and regioselective acylation. The formation of **9** was envisioned to proceed via an intramolecular pinacol coupling [30–31] of [5,5,6,6]-tetracycle **10**, which forges the A_2_ ring while simultaneously installing the hydroxy group at C30. The latter intermediate could in turn be derived from dienone **11** by an AcOH-interrupted Nazarov cyclization [32–34], thereby establishing the B ring with the desired all-*cis* stereochemical configuration, including the quaternary carbon at C10 and the essential tertiary alcohol at C1. The β-hydroxylactone moiety (D ring) in **11** could be introduced through an intramolecular aldol condensation [35] of acetate **12**. Ultimately, the preparation of **12** could be traced back to aldehyde **14** through 1,2-Grignard addition with an organomagnesium reagent [36] prepared from α-iodoenone **13**.

**Scheme 2:**
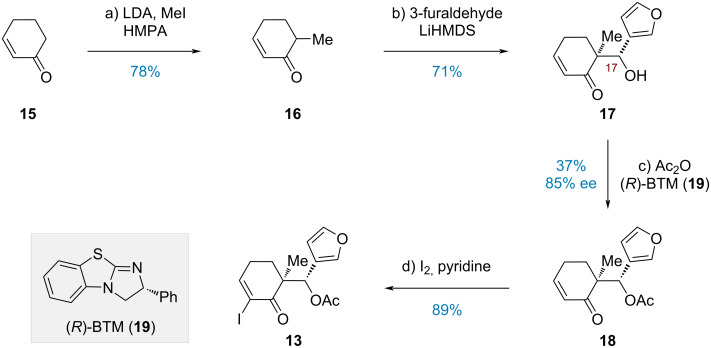
Construction of α-iodoenone **13**.

Our synthesis began with the preparation of α-iodoenone **13** (Scheme 2). The α-monomethylation of cyclohexenone **15** was efficiently carried out with LDA, HMPA, and MeI [37], producing enone **16** in 78% yield. Subsequently, a diastereoselective aldol reaction between **16** and 3-furaldehyde promoted by LiHMDS gave alcohol **17** with high regioselectivity (14:1 dr at C17) after an extensive screening of bases (LDA, NaHMDS, KHMDS, etc.). Drawing inspiration from the pioneering work of Birman [38], as well as Newhouse’s applications [18–19], an acylative kinetic resolution of the alcohol was achieved by using (*R*)-BTM (**19**), furnishing acetate **18** with satisfactory eﬃciency and enantioselectivity (37% yield, 85% ee). Finally, iodination of **18** employing Johnsen’s protocol (I_2_, pyridine) [39] provided α-iodoenone **13** in 89% yield.

**Scheme 3:**
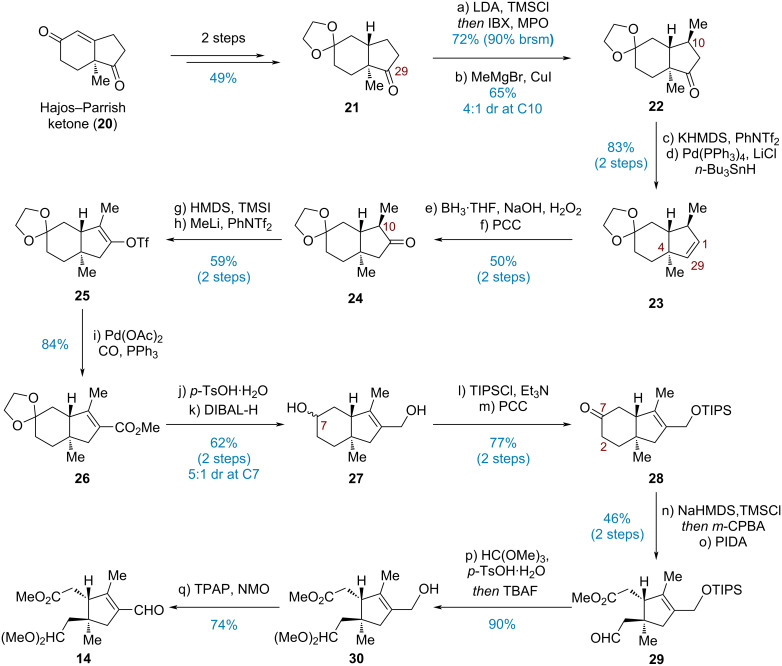
Construction of aldehyde **14**.

On the other hand, the synthesis of acetal aldehyde **14** commenced with bicyclic ketone **21**, which was prepared from (+)-Hajos–Parrish ketone in 49% yield over two steps (Scheme 3) [40–43]. Ensuring silyl enol etherification of the ketone at C29 coupled with IBX-mediated Nicolaou oxidation [44] furnished the corresponding enone in 72% yield (90% brsm). The methyl group at C10 was then introduced via a Michael addition (MeMgBr, CuI) to afford **22** in a yield of 65% (4:1 dr at C10). Initial attempts on the carbonyl 1,2-transposition protocol reported by Dong and co-workers were ineffective [45], leading to premature hydride termination and the formation of alkene **23**. As an alternative solution, by treating **22** with KHMDS and PhNTf_2_, enol triflation took place successfully. The resultant triflate was coupled with *n*-Bu_3_SnH to afford Δ^1,29^-alkene **23** in 83% yield over two steps. Subsequent hydroboration–oxidation by employing BH_3_·THF proceeded smoothly, providing a 4.4:1 mixture of regioisomeric alcohols with the desired isomer being the major component, presumably due to the considerable steric hindrance from the quaternary carbon at C4. However, decagram-scale separation of these two isomers by chromatography proved troublesome. Fortunately, the distinct reactivity of those two alcohols toward oxidation allowed for the selective conversion of desired alcohol to ketone **24** using PCC, producing a 50% overall yield, while the recovered undesired alcohol could be reverted to **21** by Swern oxidation.

Direct enol triflation (Et_3_N/Tf_2_O, NaH/PhNTf_2_, DTBMP/Tf_2_O, etc.) of **24** led only to epimerization at C10 or slow decomposition of the starting material (see Supporting Information File 1, Table S1). Pleasingly, treating **24** with HMDS and TMSI regioselectively generated the expected TMS enol ether [46], which underwent a Li/Si exchange in the presence of MeLi followed by interception of the lithium enolate with PhNTf_2_ to give enol triflate **25** in 59% overall yield. The following palladium-catalyzed methoxycarbonylation produced methyl ester **26** in a satisfactory yield of 84%. TIPS-protected allylic alcohol **28** was selected as the appropriate precursor for the α,β-unsaturated aldehyde and synthesized from **26** via a four-step transformation sequence, including deketalization, reduction, silylation of the primary alcohol and oxidation of the secondary alcohol. For the disconnection of the C2–C7 bond, a two-step protocol involving Rubottom oxidation and PIDA-promoted oxidative cleavage [47] was applied to deliver aldehyde **29**. Finally, a one-pot acetalization and desilylation effectively afforded the acetal alcohol, which was then oxidized with the aid of TPAP, furnishing acetal aldehyde **14** in 67% yield over two steps.

**Scheme 4:**
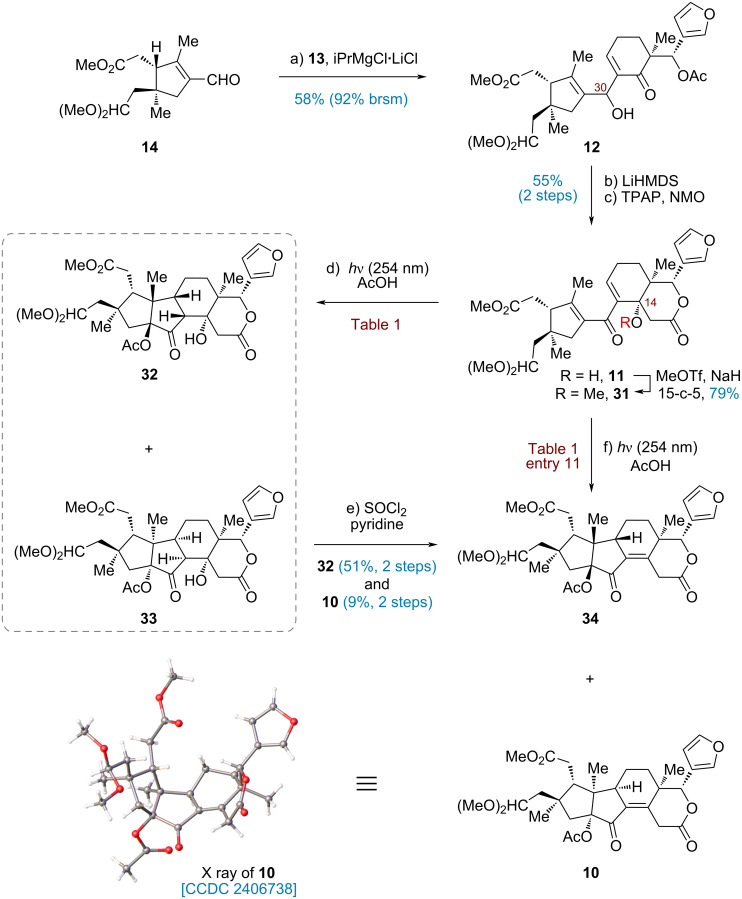
Synthesis of the advanced intermediate **10** (in the X ray structure of **10** solvent molecule is omitted for clarity).

With the two fragments **13** and **14** in hand, the next stage was set for the construction of the target skeleton through a 1,2-addition (Scheme 4). Preliminary trials to generate organometallic species via Li/I exchange under various conditions (*n*-BuLi, *t*-BuLi, or *t*-BuLi in combination with CeCl_3_ or MgBr_2_) led to rapid decomposition, likely due to inherent instability of α-iodoenone **13**. Inspired by the influential studies by Knochel [36] and Baran [48], we discovered that Mg/I exchange of **13** could be accomplished with iPrMgCl·LiCl at −78 °C. The resulting Grignard reagent reacted smoothly with aldehyde **14**, affording the corresponding adduct **12** in 58% yield (92% brsm). Since the newly created configuration at C30 was inconsequential, an intramolecular aldol reaction was directly carried out by treatment with LiHMDS to furnish β-hydroxylactone, which could be converted to dienone **11** through TPAP oxidation.

Having secured **11**, we proceeded to evaluate the pivotal Nazarov cyclization under a variety of conditions (Table 1). Initial trials under acid-mediated Nazarov conditions (AlCl_3_, BF_3_·Et_2_O and Me_2_AlCl) led to complete decomposition, while the exposure to AcOH resulted in recovery of the starting material (Table 1, entries 1–4). Recognizing the limitations of these approaches, we then turned to the milder photo-Nazarov cyclization, in which the UV-light sources were found to be critical. While irradiation at 365 or 313 nm failed to induce cyclization and only allowed recovery of the starting material (Table 1, entries 6 and 7), the disrotatory cyclization of **11** in the presence of AcOH by exposure to UV-light at 254 nm occurred exclusively to provide an inseparable mixture of **32** and **33** (Table 1, entry 5). Subsequent dehydration of the resultant mixture with SOCl_2_ and pyridine yielded separable enones **34** (51%) and **10** (9%) over two steps. The structure of **10** was unequivocally determined through X-ray crystallographic analysis (ORTEP drawing, Scheme 4). Further optimization by elevating the reaction temperature did not noticeably alter the ratio of **32** and **33** (Table 1, entries 8 and 9). Moreover, attempts to apply interrupted Nazarov cyclization with H_2_O under neutral conditions merely resulted in decomposition (Table 1, entry 10). To our delight, photoirradiation of the corresponding methyl ether **31** at 254 nm in the presence of AcOH at 20 °C led to a smooth cyclization, followed by spontaneous elimination, which produced the desired **10** as the single product, with no detection of **34** (Table 1, entry 11). This result presumably arises from the minimization of dipole–dipole repulsions between the carbonyl of the dienone moiety and the C14-OMe within the desired transition state (more details were discussed in our group’s previous work [29]).

**Table 1:** Optimization of the interrupted Nazarov cyclization.^a^

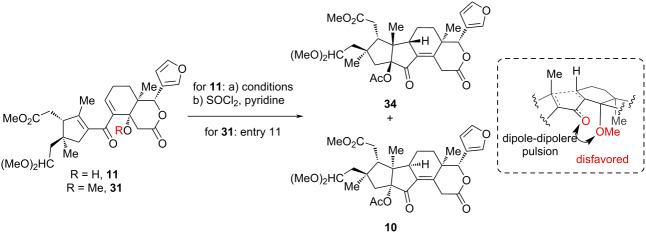			

Entry	Conditions	Yield (%)^b^	
		**34**	**10**

1^c^	**11**, AlCl_3_, DCE, 60 °C	0	0
2^c^	**11**, BF_3_·Et_2_O, DCE, 60 °C	0	0
3^c^	**11**, Me_2_AlCl, toluene, 100 °C	0	0
4	**11**, AcOH, DCE, 70 °C	0	0
5	**11**, 254 nm *h*ν, AcOH, DCM, 20 °C	57	7
6^d^	**11**, 313 nm *h*ν, AcOH, DCM, 20 °C	0	0
7^d^	**11**, 365 nm *h*ν, AcOH, DCM, 20 °C	0	0
8	**11**, 254 nm *h*ν, AcOH, DCE, 40 °C	54	8
9	**11**, 254 nm *h*ν, AcOH, DCE, 70 °C	51	9
10^c^	**11**, 254 nm *h*ν, H_2_O, DCM, 20 °C	0	0
11	**31**, 254 nm *h*ν, AcOH, DCM, 20 °C	0^e^	73^e^

^a^Reaction conditions: substrate (0.015 mmol), acid (2.0 equiv), solvent (5.0 mL). ^b^Isolated yields over two steps involving Nazarov cyclization and dehydration. ^c^Decomposition. ^d^No reaction. ^e^Isolated yields over Nazarov cyclization/elimination cascade.

## Conclusion

In conclusion, we have developed a convergent approach for the enantioselective assembly of an advanced intermediate en route to krishnolides A and C. Key steps of our strategy entail an acylative kinetic resolution of the alcohol, a 1,2-Grignard addition and an AcOH-interrupted Nazarov cyclization. Further elaboration of intermediate **10** to krishnolides A and C, as well as other khayanolide-type limonoids is currently ongoing, and the results will be disclosed in future reports.

## Supporting Information

Deposition number 2406738 contains the supplementary crystallographic data for this paper. These data can be obtained free of charge via the joint Cambridge Crystallographic Data Centre (CCDC) and Fachinformationszentrum Karlsruhe Access Structures service.

File 1 Experimental procedures, NMR spectra and other characterization data for all new compounds.

## Data Availability

All data that supports the findings of this study is available in the published article and/or the supporting information of this article.
