# Supplementary material for: A convergent synthetic approach to the tetracyclic core framework of khayanolide-type limonoids
Source: Beilstein J Org Chem. 2025 May 12;21:926–34. doi: 10.3762/bjoc.21.75 (PMC12117208; doi:10.3762/bjoc.21.75)

BEILSTEIN JOURNAL OF ORGANIC CHEMISTRY

# Supporting Information

for

# A convergent synthetic approach to the tetracyclic core framework of khayanolide-type limonoids

Zhiyang Zhang, Jialei Hu, Hanfeng Ding, Li Zhang and Peirong Rao

*Beilstein J. Org. Chem.* **2025,** *21,* 926–934. doi:10.3762/bjoc.21.75

# Experimental procedures, NMR spectra and other characterization data for all new compounds

License and Terms: This is a supporting information file under the terms of the Creative Commons Attribution License (https://creativecommons.org/licenses/by/4.0). Please note that the reuse, redistribution and reproduction in particular requires that the author(s) and source are credited and that individual graphics may be subject to special legal provisions.
The license is subject to the *Beilstein Journal of Organic Chemistry* terms and conditions: (https://www.beilstein-journals.org/bjoc/terms)

**Table of Contents**

**I) General procedures........................................................................................S2**

**II) Experimental procedures and spectroscopic data of compounds....................S3**

**III) Abbreviations .........................................................................................S33**

**IV) References ..............................................................................................S34**

**V) X-ray crystallography ..............................................................................S35**

**VI) HPLC chromatographs..............................................................................S36**

**VII) $^{1}H$ and $^{13}C$ NMR spectra of compounds......................................................S37**

## I) General procedures

All reactions were carried out under an argon atmosphere with dry solvents under anhydrous conditions, unless otherwise noted. Anhydrous methylene chloride ($CH_2Cl_2$), diisopropylamine ($iPr_2NH$) and hexamethyl phosphoryl triamide (HMPA) were distilled before use from calcium hydride. Diethyl ether ($Et_2O$), toluene, and tetrahydrofuran (THF) were distilled before use from sodium-benzophenone ketyl. Acetic acid (AcOH), acetonitrile (MeCN), acetic anhydride ($Ac_2O$), acetone, methanol (MeOH), dimethyl sulfoxide (DMSO), ethanol (EtOH), ethyl acetate (EtOAc), methyl *tert*-butyl ether (MTBE), petroleum ether (PE, b.p. 60–90 ºC), pyridine and triethylamine ($Et_3N$) were purchased at the highest commercial quality and used without further purification. Reactions that required heating were operated on a magnetic stirrer with an oil bath. Solvent degassing was conducted by bubbling with a stream of argon. Reactions were monitored by thin-layer chromatography (TLC) carried out on silica gel plates (0.4–0.5 mm) using UV light as visualizing agent and an ethanolic solution of ammonium molybdate, anisaldehyde, and heat as developing agents. Photo reactions were performed on the XPA-7 chemical reactor (Nanjing Xujiang Electromechanical Plant). Flash chromatography was performed with silica gel (200–300 mesh). NMR spectra were recorded on a Bruker AV-400 instrument and calibrated using residual undeuterated solvent as an internal reference. The following abbreviations were used to explain the multiplicities: s = singlet, d = doublet, t = triplet, q = quartet, m = multiplet, br = broad, dd = doublet of doublets, dt = doublet of triplets, td = triplet of doublets, ddd = doublet of doublet of doublets. Melting points (m.p.) were

recorded on a Büchi B-540 melting point apparatus. Optical rotation data were obtained on a PerkinElmer Model 341 Polarimeter. High-performance liquid chromatography (HPLC) analyses were performed on a Shimadzu Essentia LC-16 HPLC system, and the detection of eluent was carried out with SPD-M40 photodiode array detector at 210 nm. High-resolution mass spectra (HRMS) were recorded on Waters MALDI SYNAPT G2-Si High Definition Mass Spectrometry. X-ray diffraction data were obtained on Bruker D8 Venture, and the ORTEP drawings were generated using *Olex2* (Version 1.5) [1].

## II) Experimental procedures and spectroscopic data of compounds

### <u>Preparation of alcohol 17:</u>

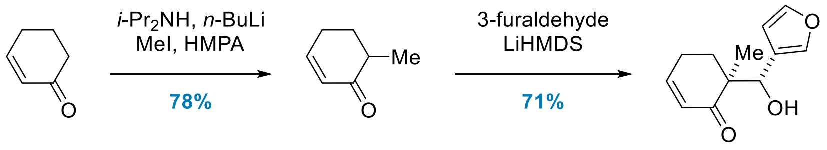


**15** **16** **17**

6-Methyl-2-cyclohexen-1-one (**16**) was prepared from 2-cyclohexen-1-one (**15**) according to known literature [2]: To a stirred solution of $iPr_2NH$ (17.5 mL, 0.125 mol, 1.2 equiv) in THF (180 mL) at –78 °C was added *n*-BuLi (52.1 mL, 2.4 M in *n*-heptane, 1.2 equiv). The resulting mixture was stirred at –78 °C for 10 min before it was warmed to 0 °C and stirred for an additional 10 min. The reaction was cooled to –78 °C before it was added **15** (10.0 g, 0.104 mol, 1.0 equiv) and stirred for further 0.5 h. The resulting mixture was added MeI (9.69 mL, 0.156 mol, 1.5 equiv) and stirred at –78 °C for 15 min before it was added HMPA (63.4 mL, 0.364 mol, 3.5 equiv). The reaction was stirred at –78 °C for an additional 2 h before it was quenched with $NH_4Cl$ (200 mL, sat.

aq.). The layers were separated, and the aqueous layer was extracted with EtOAc (3 × 200 mL). The combined organic layers were dried ($Na_2SO_4$) and concentrated in vacuo. Flash column chromatography (silica gel, PE:EtOAc 50:1) afforded enone **16** (8.93 g, 78%) as a yellow oil.

In a manner similar to [3]. To a stirred solution of enone **16** (12.1 g, 0.110 mol, 1.0 equiv) in THF (300 mL) at –78 °C was added LiHMDS (121 mL, 1.0 M in THF, 1.1 equiv). The resulting mixture was stirred at –78 °C for 0.5 h before it was added 3-furaldehyde (10.6 g, 0.110 mol, 1.0 equiv). The reaction was stirred at –78 °C for an additional 0.5 h before it was quenched with $NH_4Cl$ (200 mL, sat. aq.). The layers were separated, and the aqueous layer was extracted with EtOAc (3 × 200 mL). The combined organic layers were dried ($Na_2SO_4$) and concentrated in vacuo. Flash column chromatography (silica gel, PE:EtOAc 10:1) afforded alcohol **17** (16.1 g, 71%) as a yellow oil. **17**: $R_f$ = 0.30 (silica gel, PE:EtOAc 4:1); $[\alpha]_D^{20}$ = –0.3 (c = 1.0, $CHCl_3$); $^1$H NMR (400 MHz, $CDCl_3$) δ = 7.36 (d, $J$ = 5.2 Hz, 2 H), 7.01–6.89 (m, 1 H), 6.37 (s, 1 H), 5.95 (d, $J$ = 9.9 Hz, 1 H), 4.90 (s, 1 H), 4.46 (s, 1 H), 2.35 (s, 2 H), 1.76–1.71 (m, 1 H), 1.54 (dt, $J$ = 13.6, 4.1 Hz, 1 H), 1.19 ppm (s, 3 H); $^{13}$C NMR (100 MHz, $CDCl_3$) δ = 207.0, 150.6, 142.6, 140.7, 128.4, 124.0, 110.3, 71.5, 47.7, 31.0, 23.0, 14.8 ppm; HRMS (ESI): calcd for $C_{12}H_{14}NaO_3^+$ $[M + Na]^+$ 229.0835, found 229.0834.

**Preparation of acetate 18:**

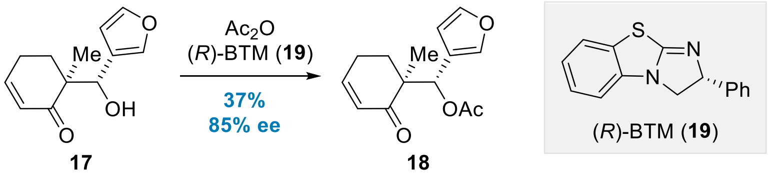


To a stirred solution of alcohol **17** (2.06 g, 10.0 mmol, 1.0 equiv) in toluene (133 mL) at –20 °C were added (*R*)-BTM [4] (378 mg, 1.50 mmol, 0.15 equiv) and $Ac_2O$ (469 µL, 5.00 mmol, 0.5 equiv). The resulting mixture was stirred at –20 °C for 24 h before it was quenched with $NaHCO_3$ (100 mL, sat. aq.). The layers were separated, and the aqueous layer was extracted with EtOAc (3 × 100 mL). The combined organic layers were dried ($Na_2SO_4$) and concentrated in vacuo. Flash column chromatography (silica gel, PE:EtOAc 8:1) afforded acetate **18** (918 mg, 37%, 85% ee) as a yellow oil, along with recovered **17** (1.19 g, 58%). **18**: $R_f$ = 0.35 (silica gel, PE:EtOAc 4:1); $[\alpha]_D^{20}$ = –2.9 (c = 1.0, $CHCl_3$); 93:7 er [Daicel Chiralpak AS-H (0.46 cm × 25 cm), *n*-hexane/2-propanol = 90/10, v = 1.0 mL·min$^{-1}$, λ = 210 nm, t (major) = 10.57 min, t (minor) = 7.57 min]; $^1$H NMR (400 MHz, $CDCl_3$) δ = 7.32 (s, 2 H), 6.88 (dt, *J* = 10.1, 4.0 Hz, 1 H), 6.37 (s, 1 H), 6.31 (br s, 1 H), 5.90 (dt, *J* = 10.1, 2.1 Hz, 1 H), 2.44–2.34 (m, 2 H), 2.07 (s, 3 H), 1.94–1.80 (m, 2 H), 1.19 ppm (s, 3 H); $^{13}$C NMR (100 MHz, $CDCl_3$) δ = 201.0, 169.7, 148.9, 142.5, 140.6, 128.5, 122.2, 110.0, 71.4, 48.9, 28.8, 22.6, 20.9, 18.7 ppm; HRMS (ESI): calcd for $C_{14}H_{16}NaO_4^+$ [M + Na]$^+$ 271.0941, found 271.0945.

## Preparation of alkenyl iodine 13:

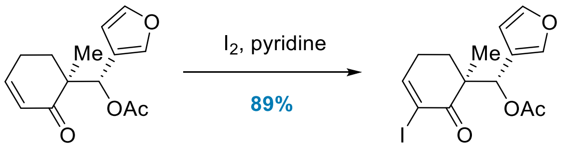


To a stirred solution of acetate **18** (3.23 g, 13.0 mmol, 1.0 equiv) in $CH_2Cl_2$ (40 mL) at 25 °C were sequentially added $I_2$ (4.30 g, 16.9 mmol, 1.3 equiv) and pyridine (1.57 mL, 19.5 mmol, 1.5 equiv). The resulting mixture was stirred at 25 °C for 3 h before it was quenched with $Na_2S_2O_3$ (50 mL, sat. aq.). The layers were separated, and the aqueous layer was extracted with EtOAc (3 × 50 mL). The combined organic layers were dried ($Na_2SO_4$) and concentrated in vacuo. Flash column chromatography (silica gel, PE:EtOAc 6:1) afforded alkenyl iodine **13** (4.33 g, 89%) as a yellow oil. **13**: $R_f$ = 0.25 (silica gel, PE:EtOAc 5:1); $[\alpha]_D^{20}$ = –4.9 (c = 1.0, $CHCl_3$); $^1H$ NMR (400 MHz, $CDCl_3$) δ = 7.63 (t, $J$ = 4.3 Hz, 1 H), 7.35–7.28 (m, 2 H), 6.34 (s, 1 H), 6.28 (br s, 1 H), 2.50–2.41 (m, 2 H), 2.07 (s, 3 H), 2.00–1.84 (m, 2 H), 1.22 ppm (s, 3 H); $^{13}C$ NMR (100 MHz, $CDCl_3$) δ = 194.1, 169.7, 157.7, 142.9, 140.9, 121.8, 110.1, 103.0, 71.6, 49.7, 29.0, 26.9, 21.0, 19.3 ppm; HRMS (ESI): calcd for $C_{14}H_{15}INaO_4^+$ $[M + Na]^+$ 396.9907, found 396.9916.

## Preparation of diketone S-1:

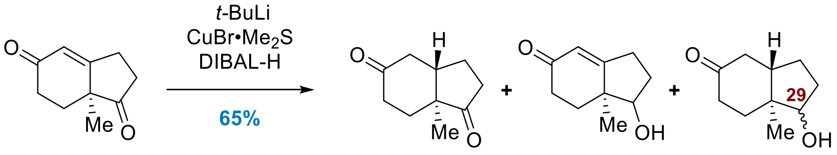

Diketone **S-1** was prepared from Hajos–Parrish ketone **20** according to known literature [5]: To a stirred solution of CuBr·$Me_2S$ (15.0 g, 72.8 mmol, 0.40 equiv) in THF (70 mL) at –78 °C was added *t*-BuLi (61.6 mL, 1.3 M in pentane, 0.44 equiv). The resulting mixture was warmed to –50 °C and stirred for 15 min before it was added HMPA (41.4 mL, 0.237 mol, 1.3 equiv). The reaction was cooled to –78 °C before it was added a solution of **20** (29.9 g, 0.182 mol, 1.0 equiv) in THF (40 mL). After stirred at –78 °C for an additional 15 min, the resulting mixture was added a solution of DIBAL-H (243 mL, 1.5 M in toluene, 2.0 equiv) in HMPA (90 mL) and slowly warmed to –40 °C. The reaction was stirred at –40 °C for further 2 h before it was quenched with HCl (500 mL, 2.0 M in $H_2O$). The layers were separated, and the aqueous layer was extracted with EtOAc (6 × 100 mL). The combined organic layers were dried ($Na_2SO_4$) and concentrated in vacuo. Flash column chromatography (silica gel, PE:EtOAc 10:1) afforded diketone **S-1** (16.0 g, 53%), enone **S-2** (9.07 g, 30%) and alcohol **S-3** (2.14 g, 7%) as yellow oils.

**S-1**: $R_f$ = 0.30 (silica gel, PE:EtOAc 1:1); $[\alpha]_D^{20}$ = +72.3 (c = 1.0, $CHCl_3$); $^1H$ NMR (400 MHz, $CDCl_3$) δ = 2.61–2.36 (m, 5 H), 2.29–2.17 (m, 1 H), 2.15–2.03 (m, 1 H), 2.03–1.92 (m, 2 H), 1.75–1.64 (m, 2 H), 1.10 ppm (s, 3 H); $^{13}C$ NMR (100 MHz, $CDCl_3$) δ = 218.2, 209.6, 46.8, 44.5, 42.4, 36.9, 36.2, 29.7, 23.9, 12.6 ppm; HRMS (ESI): calcd for $C_{10}H_{14}NaO_2^+$ $[M + Na]^+$ 189.0886, found 189.0896.

**S-2**: $R_f$ = 0.2 (silica gel, PE:EtOAc 1:1); $[\alpha]_D^{20}$ = +37.6 (c = 1.0, $CHCl_3$); $^1H$ NMR (400 MHz, $CDCl_3$) δ = 5.79 (s, 1 H), 3.85 (dd, *J* = 10.3, 7.6 Hz, 1 H), 2.71 (br dd, *J* = 19.7, 11.6 Hz, 1 H), 2.59–2.47 (m, 1 H), 2.47–2.33 (m, 2 H), 2.18–2.08 (m, 2 H), 1.85–

1.76 (m, 2 H), 1.15 ppm (s, 3 H); $^{13}$C NMR (100 MHz, CDCl$_3$) δ = 199.4, 175.3, 123.6, 80.77, 45.3, 34.2, 33.4, 29.3, 26.6, 15.2 ppm; HRMS (ESI): calcd for $C_{10}H_{14}NaO_2^+$ [M + Na]$^+$ 189.0886, found 189.0885.

**S-3**: $R_f$ = 0.25 (silica gel, PE:EtOAc 1:1, 2:1 dr at C29); $[\alpha]_D^{20}$ = +52.3 (c = 1.0, CHCl$_3$); $^1$H NMR (400 MHz, CDCl$_3$) δ = 3.89–3.82 (m, 0.3 H), 3.76 (t, $J$ = 8.5 Hz, 0.7 H), 2.46–2.15 (m, 5 H), 2.06–1.88 (m, 1 H), 1.85–1.35 (m, 6 H), 1.18 (s, 1 H), 1.00 ppm (s, 2 H); $^{13}$C NMR (100 MHz, CDCl$_3$) δ = 213.0, 211.6, 80.5, 80.1, 44.8, 43.9, 43.3, 43.0, 42.6, 42.0, 37.4, 37.0, 34.8, 32.2, 32.1, 31.3, 28.5, 25.4, 19.5, 9.9 ppm; HRMS (ESI): calcd for $C_{10}H_{16}NaO_2^+$ [M + Na]$^+$ 191.1043, found 191.1042.

**Recycle of enone S-2:**

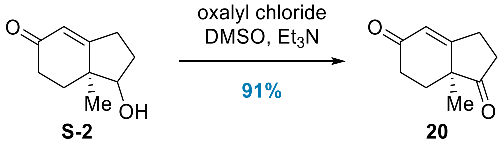


To a stirred solution of oxalyl chloride (6.05 mL, 70.9 mmol, 1.3 equiv) in $CH_2Cl_2$ (90 mL) at –78 °C was added DMSO (5.81 mL, 81.9 mmol, 1.5 equiv). The resulting mixture was stirred at –78 °C for 20 min before it was added a solution of alcohol **S-2** (9.07 g, 54.6 mmol, 1.0 equiv) in $CH_2Cl_2$ (10 mL) and stirred for an additional 30 min. The reaction was added $Et_3N$ (15.2 mL, 0.109 mol, 2.0 equiv) and stirred at –78 °C for further 30 min before it was quenched with $NaHCO_3$ (100 mL, sat. aq.). The layers were separated, and the aqueous layer was extracted with $CH_2Cl_2$ (3 × 80 mL). The combined organic layers were dried ($Na_2SO_4$) and concentrated in vacuo. Flash column chromatography (silica gel, PE:EtOAc 10:1) afforded Hajos–Parrish ketone **20** (8.16 g, 91%).

**Recycle of alcohol S-3:**

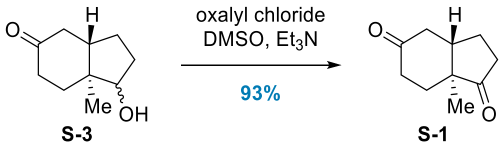


To a stirred solution of oxalyl chloride (1.63 mL, 19.1 mmol, 1.5 equiv) in $CH_2Cl_2$ (20 mL) at –78 °C was added DMSO (1.45 mL, 20.4 mmol, 1.6 equiv). The resulting mixture was stirred at –78 °C for 20 min before it was added a solution of alcohol **S-3** (2.14 g, 12.7 mmol, 1.0 equiv) in $CH_2Cl_2$ (5 mL) and stirred for an additional 30 min. The reaction was added $Et_3N$ (3.54 mL, 25.4 mmol, 2.0 equiv) and stirred at –78 °C for further 30 min before it was quenched with $NaHCO_3$ (50 mL, sat. aq.). The layers were separated, and the aqueous layer was extracted with $CH_2Cl_2$ (3 × 30 mL). The combined organic layers were dried ($Na_2SO_4$) and concentrated in vacuo. Flash column chromatography (silica gel, PE:EtOAc 10:1) afforded diketone **S-1** (1.97 g, 93%).

**Preparation of ketal 21:**

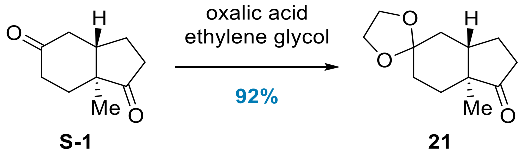


Ketal **21** was prepared from diketone **S-1** according to known literature [6]: To a stirred solution of diketone **S-1** (28.2 g, 170 mmol, 1.0 equiv) in MeCN (200 mL) at 25 °C were sequentially added ethylene glycol (94.6 mL, 1.70 mol, 10 equiv) and oxalic acid (7.64 g, 84.9 mmol, 0.5 equiv). The reaction was stirred at 25 °C for 2 h before it was quenched with $NaHCO_3$ (200 mL, sat. aq.). The layers were separated, and the aqueous layer was extracted with EtOAc (3 × 200 mL). The combined organic layers

were dried ($Na_2SO_4$) and concentrated in vacuo. Flash column chromatography (silica gel, PE:EtOAc 10:1) afforded ketal **21** (32.9 g, 92%) as a yellow oil. **21**: $R_f$ = 0.35 (silica gel, PE:EtOAc 5:1); $[\alpha]_D^{20}$ = –48.0 (c = 1.0, $CHCl_3$); $^1H$ NMR (400 MHz, $CDCl_3$) δ = 3.93 (t, *J* = 4.3 Hz, 4 H), 2.44 (dd, *J* = 19.3, 8.7 Hz, 1 H), 2.20–2.07 (m, 1 H), 2.07–1.94 (m, 1 H), 1.90–1.80 (m, 1 H), 1.76–1.66 (m, 5 H), 1.65–1.55 (m, 1 H), 1.48 (td, *J* = 13.8, 6.4 Hz, 1 H), 0.91 ppm (s, 3 H); $^{13}C$ NMR (100 MHz, $CDCl_3$) δ = 219.7, 109.6, 64.6, 64.4, 47.1, 42.8, 36.2, 35.6, 30.8, 28.6, 23.6, 12.2 ppm; HRMS (ESI): calcd for $C_{12}H_{18}NaO_3^+$ $[M + Na]^+$ 233.1148, found 233.1149.

**Preparation of enone S-4:**

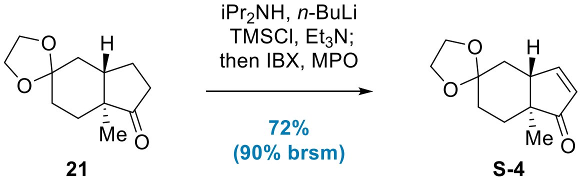


To a stirred solution of $iPr_2NH$ (53.7 mL, 0.383 mol, 1.2 equiv) in THF (500 mL) at –78 °C was added *n*-BuLi (160 mL, 2.4 M in *n*-heptane, 1.2 equiv). The reaction was stirred at –78 °C for 10 min before it was warmed to 0 °C and stirred for an additional 10 min. The resulting mixture was cooled to –78 °C and added a solution of ketone **21** (67.2 g, 0.320 mol, 1.0 equiv) in THF (70 mL). After stirred at –78 °C for further 0.5 h, the reaction was added $Et_3N$ (53.3 mL, 0.383 mol, 1.2 equiv) and TMSCl (48.7 mL, 0.383 mol, 1.2 equiv). The resulting mixture was warmed to 25 °C and stirred for 1 h before it was quenched with $NaHCO_3$ (500 mL, sat. aq.). The layers were separated, and the aqueous layer was extracted with EtOAc (5 × 400 mL). The combined organic layers were washed with brine (200 mL), dried ($Na_2SO_4$) and concentrated in vacuo to

afford the crude silyl enol ether as a yellow oil, which was used directly without further purification.

To a stirred solution of IBX (107 g, 0.384 mol, 1.2 equiv) in DMSO (400 mL) at 25 °C was added MPO (20.0 g, 0.160 mol, 0.5 equiv). The resulting mixture was stirred at 25 °C for 1 h before it was cooled to 0 °C and added a solution of silyl enol ether (crude, obtained above) in DMSO (100 mL). The reaction was warmed to 25 °C and stirred for an additional 2 h before it was quenched with $H_2O$ (400 mL). The resulting mixture was filtered through a short pad of celite, and the filtrate was extracted with MTBE (5 × 100 mL). The combined filtering liquors were separated, and the aqueous layer was extracted with EtOAc (5 × 300 mL). The combined organic layers were dried ($Na_2SO_4$) and concentrated in vacuo. Flash column chromatography (silica gel, PE:EtOAc 10:1) afforded enone **S-4** (48.0 g, 72%) as a yellow oil, along with recovered ketone **21** (13.4 g, 20%). **S-4**: $R_f$ = 0.30 (silica gel, PE:EtOAc 5:1); $[\alpha]_D^{20}$ = –24.3 (c = 1.0, $CHCl_3$); $^1H$ NMR (400 MHz, $CDCl_3$) δ = 7.37 (dd, $J$ = 5.9, 1.9 Hz, 1H), 6.02 (dd, $J$ = 6.0, 3.2 Hz, 1 H), 3.99–3.92 (m, 4 H), 3.08–3.00 (m, 1 H), 1.95–1.87 (m, 3 H), 1.78–1.67 (m, 3 H), 1.13 ppm (s, 3 H); $^{13}C$ NMR (100 MHz, $CDCl_3$) δ = 211.8, 160.6, 132.0, 109.7, 64.8, 64.3, 50.7, 48.0, 33.6, 31.7, 26.8, 19.4 ppm; HRMS (ESI): calcd for $C_{12}H_{16}NaO_3^+$ $[M + Na]^+$ 231.0992, found 231.0989.

**Preparation of ketone 22:**

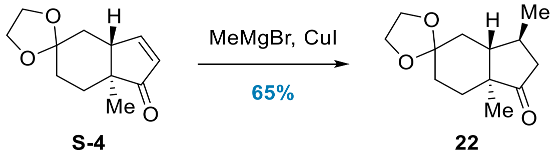

To a stirred solution of CuI (34.2 g, 0.180 mol, 2.0 equiv) in THF (500 mL) at –78 °C was added MeMgBr (120 mL, 3.0 M in $Et_2O$, 4.0 equiv). The resulting mixture was stirred at –78 °C for 15 min before it was added a solution of enone **S-4** (18.7 g, 90.0 mmol, 1.0 equiv) in THF (100 mL). The reaction was stirred at –78 °C for an additional 0.5 h before it was quenched with $NH_4Cl$ (400 mL, sat aq.). The layers were separated, and the aqueous layer was extracted with EtOAc (3 × 300 mL). The combined organic layers were dried ($Na_2SO_4$) and concentrated in vacuo. Flash column chromatography (silica gel, PE:EtOAc 8:1) afforded ketone **22** (13.1 g, 65%) as a yellow oil. **22**: $R_f$ = 0.40 (silica gel, PE:EtOAc 5:1); $[\alpha]_D^{20}$ = –104.5 (c = 1.0, $CHCl_3$); $^1$H NMR (400 MHz, $CDCl_3$) δ = 3.99–3.87 (m, 4 H), 2.48 (dd, $J$ = 19.0, 8.8 Hz, 1 H), 2.39–2.23 (m, 2 H), 2.18 (ddd, $J$ = 13.3, 7.6, 2.9 Hz, 1 H), 1.84 (t, $J$ = 13.2 Hz, 1 H), 1.75–1.58 (m, 4 H), 1.45 (td, $J$ = 12.8, 5.6 Hz, 1 H), 1.12–0.99 ppm (m, 6 H); $^{13}$C NMR (100 MHz, $CDCl_3$) δ = 220.1, 110.1, 64.6, 64.3, 46.8, 44.7, 44.3, 33.3, 31.0, 30.8, 28.9, 17.1, 16.5 ppm; HRMS (ESI): calcd for $C_{13}H_{20}NaO_3^+$ $[M + Na]^+$ 247.1305, found 247.1304.

**<u>Preparation of enol triflate S-5:</u>**

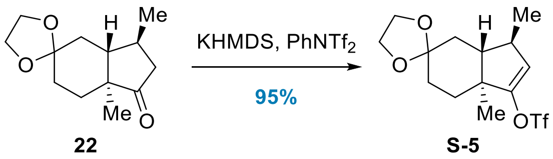


To a stirred solution of ketone **22** (31.4 g, 0.140 mol, 1.0 equiv) and $PhNTf_2$ (55.0 g, 0.154 mol, 1.1 equiv) in THF (400 mL) at –78 °C was added KHMDS (154 mL, 1.0 M in THF, 1.1 equiv). The reaction was stirred at –78 °C for 1 h before it was quenched with $NH_4Cl$ (300 mL, sat aq.). The layers were separated, and the aqueous layer was

extracted with EtOAc (3 × 300 mL). The combined organic layers were dried ($Na_2SO_4$) and concentrated in vacuo. Flash column chromatography (silica gel, PE:EtOAc 10:1) afforded enol triflate **S-5** (47.4 g, 95%) as a yellow oil. **S-5**: $R_f$ = 0.50 (silica gel, PE:EtOAc 10:1); $[\alpha]_D^{20}$ = –8.2 (c = 1.0, $CHCl_3$); $^1$H NMR (400 MHz, $CDCl_3$) δ = 5.64 (d, $J$ = 3.1 Hz, 1 H), 4.02–3.90 (m, 4 H), 2.70–2.60 (m, 1 H), 2.39 (ddd, $J$ = 14.1, 7.5, 2.8 Hz, 1 H), 1.86–1.73 (m, 3H), 1.71–1.59 (m, 3 H), 1.23 (s, 3 H), 1.00 ppm (d, $J$ = 7.4 Hz, 3 H); $^{13}$C NMR (100 MHz, $CDCl_3$) δ = 158.1, 120.4, 118.7 (q, $J_{C–F}$ = 318.4 Hz), 109.9, 64.7, 64.3, 46.5, 45.1, 36.8, 32.7, 32.4, 31.7, 19.9, 14.1 ppm; HRMS (ESI): calcd for $C_{14}H_{19}F_3NaO_5S^+$ $[M + Na]^+$ 379.0798, found 379.0799.

**Preparation of alkene 23:**

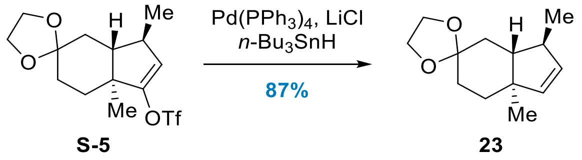


To a stirred solution of enol triflate **S-5** (49.9 g, 0.140 mol, 1.0 equiv) in THF (300 mL) at 25 °C were sequentially added LiCl (6.53 g, 0.154 mol, 1.1 equiv), $Pd(PPh_3)_4$ (4.85 g, 4.20 mmol, 0.03 equiv) and *n*-$Bu_3SnH$ (41.5 mL, 0.154 mol, 1.1 equiv). The resulting mixture was warmed to 50 °C and stirred for 2 h before it was cooled to 25 °C and quenched with brine (300 mL, sat aq.). The layers were separated, and the aqueous layer was extracted with EtOAc (3 × 200 mL). The combined organic layers were dried ($Na_2SO_4$) and concentrated in vacuo. Flash column chromatography (silica gel, PE:EtOAc 10:1) afforded alkene **23** (25.3 g, 87%) as a yellow oil. **23**: $R_f$ = 0.55 (silica gel, PE:EtOAc 10:1); $[\alpha]_D^{20}$ = –67.2 (c = 1.0, $CHCl_3$); $^1$H NMR (400 MHz, $CDCl_3$) δ =

5.82 (d, $J$ = 5.8 Hz, 1 H), 5.72 (dd, $J$ = 5.8, 2.8 Hz, 1 H), 3.94 (s, 4 H), 2.63–2.43 (m, 1 H), 2.10 (ddd, $J$ = 13.7, 7.7, 3.2 Hz, 1 H), 1.85 (td, $J$ = 13.6, 5.8 Hz, 1 H), 1.76–1.48 (m, 5 H), 1.01 (s, 3 H), 0.91 ppm (d, $J$ = 7.5 Hz, 3 H); $^{13}$C NMR (100 MHz, $CDCl_3$) δ = 141.2, 135.7, 110.8, 64.5, 64.2, 47.8, 46.1, 40.7, 35.2, 32.9, 32.4, 21.8, 14.7 ppm; HRMS (ESI): calcd for $C_{13}H_{20}NaO_2^+$ $[M + Na]^+$ 231.1356, found 231.1356.

**Preparation of alcohols S-6 and S-7 (0.8 mmol scale):**

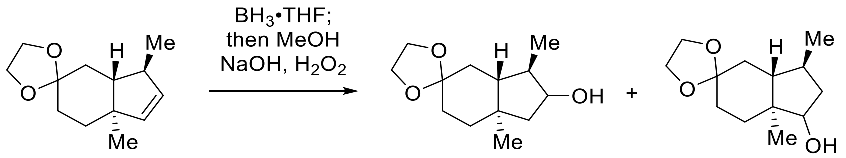

**23** **S-6** (70%) **S-7** (16%)

To a stirred solution of alkene **23** (170 mg, 0.816 mmol, 1.0 equiv) in THF (10 mL) at 0 °C was added $BH_3$·THF (816 µL, 1.0 M in THF, 1.0 equiv). The resulting mixture was stirred at 0 °C for 1 h before it was sequentially added MeOH (331 µL, 8.16 mmol, 10 equiv), NaOH (54.5 µL, 3.0 M in $H_2O$, 2.0 equiv) and $H_2O_2$ (166 µL, 30 wt % in $H_2O$, 2.0 equiv). The reaction was warmed to 50 °C and stirred for an additional 1 h before it was cooled to 25 °C and quenched with $NH_4Cl$ (20 mL, sat aq.). The layers were separated, and the aqueous layer was extracted with EtOAc (3 × 20 mL). The combined organic layers were dried ($Na_2SO_4$) and concentrated in vacuo. Flash column chromatography (silica gel, PE:EtOAc 3:1) afforded alcohols **S-6** (0.13 g, 70%) and **S-7** (30 mg, 16% ) as colorless oils.

**S-6**: $R_f$ = 0.40 (silica gel, PE:EtOAc 1:1); $[\alpha]_D^{20}$ = +6.5 (c = 1.0, $CHCl_3$); $^1$H NMR (400 MHz, $CDCl_3$) δ = 4.11 (ddd, $J$ = 8.7, 6.9, 3.7 Hz, 1 H), 3.92 (br s, 4 H), 2.13 (td, $J$ = 11.1, 5.6 Hz, 1 H), 2.07–2.01 (m, 2 H), 1.94–1.84 (m, 1 H), 1.70 (td, $J$ = 13.9, 4.8

Hz, 1 H), 1.61–1.48 (m, 4 H), 1.41 (td, $J$ = 13.3, 4.2 Hz, 1 H), 1.20 (dd, $J$ = 11.7, 8.5 Hz, 1 H), 0.97 (d, $J$ = 7.7 Hz, 3 H), 0.88 ppm (s, 3 H); $^{13}$C NMR (100 MHz, $CDCl_3$) δ = 110.5, 82.1, 64.4, 64.2, 50.4, 46.2, 43.7, 41.4, 37.1, 32.7, 31.1, 19.5, 16.5 ppm; HRMS (ESI): calcd for $C_{13}H_{22}NaO_3^+$ [M + Na]$^+$ 249.1461, found 249.1463.

**S-7**: $R_f$ = 0.42 (silica gel, PE:EtOAc 1:1); $[\alpha]_D^{20}$ = +10.3 (c = 1.0, $CHCl_3$); $^1$H NMR (400 MHz, methanol-$d_4$) δ = 3.92 (br s, 4 H), 3.73 (d, $J$ = 5.2 Hz, 1 H), 2.31–2.16 (m, 2 H), 1.96 (dd, $J$ = 14.9, 9.0 Hz, 1 H), 1.84–1.62 (m, 7 H), 1.31–1.25 (m, 1 H), 0.89 (d, $J$ = 7.4 Hz, 3 H), 0.87 ppm (s, 3 H); $^{13}$C NMR (100 MHz, methanol-$d_4$) δ = 111.7, 80.3, 65.3, 65.2, 46.8, 43.1, 43.0, 33.8, 32.5, 32.1, 31.3, 18.9, 18.6 ppm; HRMS (ESI): calcd for $C_{13}H_{22}NaO_3^+$ [M + Na]$^+$ 249.1461, found 249.1463.

**Preparation of ketone 24 (decagram scale):**

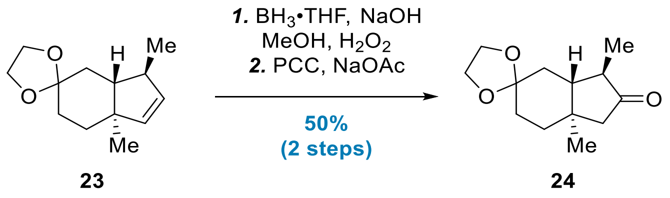


To a stirred solution of alkene **23** (17.3 g, 83.1 mmol, 1.0 equiv) in THF (150 mL) at 0 °C was added $BH_3 \cdot THF$ (83.1 mL, 1.0 M in THF, 1.0 equiv). The resulting mixture was stirred at 0 °C for 1 h before it was sequentially added MeOH (33.6 mL, 0.831 mol, 10 equiv), NaOH (55.4 mL, 3.0 M in $H_2O$, 2.0 equiv) and $H_2O_2$ (16.9 mL, 30 wt % in $H_2O$, 2.0 equiv). The reaction was warmed to 50 °C and stirred for an additional 1 h before it was cooled to 25 °C and quenched with $NH_4Cl$ (200 mL, sat aq.). The layers were separated, and the aqueous layer was extracted with EtOAc (3 × 200 mL). The combined organic layers were dried ($Na_2SO_4$) and concentrated in vacuo. Flash column

chromatography (silica gel, PE:EtOAc 2:1) afforded a mixture of alcohols **S-6** and **S-7** (16.5 g, 88%) as a yellow oil.

To a stirred solution of alcohols **S-6** and **S-7** (obtained above, 16.5 g, 72.9 mmol, 1.0 equiv) in $CH_2Cl_2$ (200 mL) at 25 °C were sequentially added NaOAc (29.9 g, 0.364 mol, 5.0 equiv) and PCC (23.6 g, 0.109 mol, 1.5 equiv). The reaction was stirred for 1 h before it was added $Et_2O$ (200 mL). The resulting mixture was filtered through a short pad of celite, and the filtrate was extracted with MTBE (3 × 100 mL). The combined organic layers were quenched with $NaHCO_3$ (200 mL, sat, aq.). The layers were separated, and the aqueous layer was extracted with EtOAc (3 × 200 mL). The combined organic layers were dried ($Na_2SO_4$) and concentrated in vacuo. Flash column chromatography (silica gel, PE:EtOAc 5:1) afforded ketone **24** (9.32 g, 50% over 2 steps) as a yellow oil, along with recovered **S-7** (2.82 g, 15% over 2 steps). **24**: $R_f = 0.4$ (silica gel, PE:EtOAc 5:1); $[\alpha]_D^{20} = +12.6$ (c = 1.0, $CHCl_3$); $^1H$ NMR (400 MHz, $CDCl_3$) δ = 3.93 (s, 4 H), 2.40–2.22 (m, 2 H), 2.09 (q, $J$ = 16.0 Hz, 2 H), 1.82–1.52 (m, 6 H), 0.99 (d, $J$ = 7.4 Hz, 3 H), 0.91 ppm (s, 3 H); $^{13}C$ NMR (100 MHz, $CDCl_3$) δ = 221.7, 109.5, 64.5, 64.3, 55.1, 44.9, 43.3, 38.7, 36.3, 32.2, 31.7, 19.1, 12.9 ppm; HRMS (ESI): calcd for $C_{13}H_{20}NaO_3^+$ $[M + Na]^+$ 247.1305, found 247.1306.

**Recycle of alcohol S-7:**

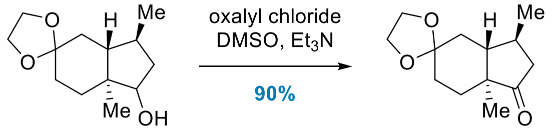


**S-7** **22**

To a stirred solution of oxalyl chloride (1.59 mL, 18.7 mmol, 1.5 equiv) in $CH_2Cl_2$ (20 mL) at –78 °C was added DMSO (1.42 mL, 19.9 mmol, 1.6 equiv). The resulting mixture was stirred at –78 °C for 20 min before it was added a solution of alcohol **S-7** (2.82 g, 12.5 mmol, 1.0 equiv) in $CH_2Cl_2$ (5 mL) and stirred for an additional 0.5 h. The reaction was added $Et_3N$ (3.46 mL, 24.9 mmol, 2.0 equiv) and stirred at –78 °C for further 0.5 h before it was quenched with $NaHCO_3$ (50 mL, sat. aq.). The layers were separated, and the aqueous layer was extracted with $CH_2Cl_2$ (3 × 30 mL). The combined organic layers were dried ($Na_2SO_4$) and concentrated in vacuo. Flash column chromatography (silica gel, PE:EtOAc 10:1) afforded ketone **22** (2.52 g, 90%).

**Table S1.** Optimization on preparation of enol triflate **25**

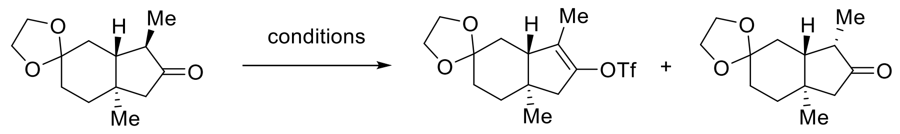


**24** **25** **24'**

| entry | conditions[a] | result (yield[b]) |
|---|---|---|
| 1 | *t*-BuOK (5.0 equiv), $Tf_2O$ (2.0 equiv), THF, 20 °C | no reaction |
| 2 | $Et_3N$ (5.0 equiv), $Tf_2O$ (2.0 equiv), DCE, 60 °C | no reaction |
| 3 | DTBMP (5.0 equiv), $Tf_2O$ (2.0 equiv), $CH_2Cl_2$, 40 °C | **24′** (41%) |
| 4 | NaH (5.0 equiv), $Tf_2O$ (2.0 equiv), THF, 20 °C | **24′** (39%) |
| 5 | KH (5.0 equiv), $Tf_2O$ (2.0 equiv), THF, 20 °C | decomposition |
| 6 | NaH (5.0 equiv), $PhNTf_2$ (2.0 equiv), THF, 78°C | **24′** (36%) |
| 7 | NaH (5.0 equiv), Comins' reagent (2.0 equiv), THF, 78°C | decomposition |
| 8 | HMDS (5.0 equiv), TMSCl (2.0 equiv), LiCl (2.0 equiv), $CH_2Cl_2$; then MeLi (2.0 equiv), $PhNTf_2$ (2.0 equiv), THF | **25** (61%[c]) |
| 9[d] | HMDS (2.0 equiv), TMSCl (1.5 equiv), LiCl (1.5 equiv), $CH_2Cl_2$; then MeLi (1.0 equiv), $PhNTf_2$ (1.0 equiv), THF | **25** (59%[c]) |

[a]Reaction conditions: **24** (1.0 mmol), solvent (10 mL); [b]Isolated yields; [c]Yield over 2 steps; [d]Reaction was performed on 13.0 mmol scale.

**Preparation of ketone 24′ (entry 3, Table S1):**

To a stirred solution of ketone **24** (224 mg, 1.0 mmol, 1.0 equiv) in $CH_2Cl_2$ (10 mL) at 0 °C were sequentially added DTBMP (696 µL, 5.0 mmol, 5.0 equiv) and $Tf_2O$ (336 µL, 2.0 mmol, 2.0 equiv). The resulting mixture was warmed to 40 °C and stirred for 2 h before it was cooled to 0 °C and quenched with $NaHCO_3$ (10 mL, sat. aq.). The layers were separated, and the aqueous layer was extracted with EtOAc (3 × 8 mL). The combined organic layers were dried ($Na_2SO_4$) and concentrated in vacuo. Flash column chromatography (silica gel, PE:EtOAc 10:1) afforded ketone **24′** (91.9 mg, 41%) as a yellow oil. **24′**: $R_f$ = 0.4 (silica gel, PE:EtOAc 5:1); $[\alpha]_D^{20}$ = +35.7 (c = 1.0, $CHCl_3$); $^1$H NMR (400 MHz, $CDCl_3$) δ = 3.96 (d, $J$ = 2.0 Hz, 4 H), 2.18 (d, $J$ = 16.4 Hz, 1 H), 2.01–1.63 (m, 8 H), 1.59–1.50 (m, 1 H), 1.02 (d, $J$ = 6.9 Hz, 3 H), 0.94 ppm (s, 3 H); $^{13}$C NMR (100 MHz, $CDCl_3$) δ = 220.5, 109.1, 64.5, 64.3, 53.6, 50.0, 45.4, 36.7, 35.6, 33.9, 31.8, 17.6, 12.8 ppm; HRMS (ESI): calcd for $C_{13}H_{20}NaO_3^+$ $[M + Na]^+$ 247.1305, found 247.1302.

**Preparation of enol triflate 25 (entry 9, Table S1):**

To a stirred solution of ketone **24** (2.91 g, 13.0 mmol, 1.0 equiv) in $CH_2Cl_2$ (130 mL) at 0 °C were sequentially added HMDS (5.47 mL, 25.9 mmol, 2.0 equiv), TMSCl (2.47 mL, 19.5 mmol, 1.5 equiv) and LiI (2.61 g, 19.5 mmol, 1.5 equiv). The reaction was stirred at 0 °C for 1.5 h before it was quenched with $NaHCO_3$ (80 mL, sat. aq.). The layers were separated, and the aqueous layer was extracted with $CH_2Cl_2$ (3 × 80 mL). The combined organic layers were dried ($Na_2SO_4$) and concentrated in vacuo.

Flash column chromatography (silica gel, PE:EtOAc:$Et_3N$ 100:1:1) afforded silyl enol ether (2.46 g, 64%) as a yellow oil, along with recovered **24** (349 mg, 12%).

To a stirred solution of silyl enol ether (2.46 g, 8.31 mmol, 1.0 equiv) in THF (60 mL) at 0 °C was added MeLi (7.79 mL, 1.6 M in THF, 1.5 equiv). The resulting mixture was stirred at 0 °C for 15 min before it was added $PhNTf_2$ (4.45 g, 12.5 mmol, 1.5 equiv). The reaction was stirred at 0 °C for an additional 0.5 h before it was quenched with $NH_4Cl$ (50 mL, sat. aq.). The layers were separated, and the aqueous layer was extracted with EtOAc (3 × 80 mL). The combined organic layers were dried ($Na_2SO_4$) and concentrated *in vacuo*. Flash column chromatography (silica gel, PE:EtOAc 10:1) afforded enol triflate **25** (2.72 g, 92%) as a yellow oil. **25**: $R_f$ = 0.45 (silica gel, PE:EtOAc 10:1); $[\alpha]_D^{20}$ = +132.3 (c = 1.0, $CHCl_3$); $^1H$ NMR (400 MHz, $CDCl_3$) δ = 3.96 (d, $J$ = 2.3 Hz, 4 H), 2.62 (dd, $J$ = 10.7, 7.4 Hz, 1 H), 2.44 (dt, $J$ = 14.2, 3.2 Hz, 1 H), 2.18 (d, $J$ = 14.1 Hz, 1 H), 1.89–1.79 (m, 1 H), 1.78–1.67 (m, 3 H), 1.67–1.59 (m, 5 H), 1.02 ppm (s, 3 H); $^{13}C$ NMR (100 MHz, $CDCl_3$) δ = 144.4, 129.6, 118.6 (q, $J_{C-F}$ = 319.9 Hz), 109.3, 64.6, 64.3, 49.7, 45.1, 42.9, 33.5, 32.0, 31.8, 17.9, 10.1 ppm; HRMS (ESI): calcd for $C_{14}H_{19}F_3NaO_5S^+$ $[M + Na]^+$ 379.0798, found 379.0798.

**<u>Preparation of ester 26:</u>**

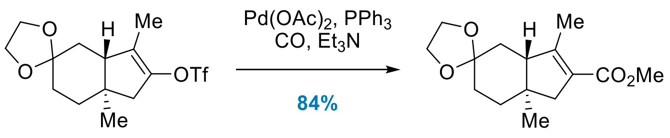


25 26

To a stirred solution of enol triflate **25** (2.92 g, 8.20 mmol, 1.0 equiv) in MeOH (20 mL) and DMF (20 mL) at 25 °C were sequentially added $PPh_3$ (172 mg, 0.656

mmol, 0.08 equiv), $Et_3N$ (2.28 mL, 16.4 mmol ,2.0 equiv) and $Pd(OAc)_2$ (73.6 mg, 0.328 mmol, 0.04 equiv). The resulting mixture was degassed with CO for 5 min before it was warmed to 50 °C. The reaction was stirred at 50 °C for 10 h in CO atmosphere before it was cooled to 25 °C and quenched with brine (50 mL, sat. aq.). The layers were separated, and the aqueous layer was extracted with EtOAc (3 × 50 mL). The combined organic layers were dried ($Na_2SO_4$) and concentrated in vacuo. Flash column chromatography (silica gel, PE:EtOAc 20:1) afforded ester **26** (1.83 g, 84%) as a yellow oil. **26**: $R_f$ = 0.50 (silica gel, PE:EtOAc 5:1); $[\alpha]_D^{20}$ = +45.8 (c = 1.0, $CHCl_3$); $^1H$ NMR (400 MHz, $CDCl_3$) δ = 3.96 (br s, 4 H), 3.70 (s, 3 H), 2.59 (d, $J$ = 13.7 Hz, 1 H), 2.35 (d, $J$ = 14.2 Hz, 1 H), 2.19 (dt, $J$ = 14.2, 3.0 Hz, 1 H), 2.03 (s, 3 H), 1.88–1.75 (m, 2 H), 1.68–1.63 (m, 4 H), 0.86 ppm (s, 3 H); $^{13}C$ NMR (100 MHz, $CDCl_3$) δ = 167.3, 157.8, 127.3, 109.9, 64.6, 64.3, 55.7, 51.0, 45.7, 42.9, 33.8, 32.1, 31.9, 17.4, 14.2 ppm; HRMS (ESI): calcd for $C_{15}H_{22}NaO_4^+$ $[M + Na]^+$ 289.1410, found 289.1412.

**<u>Preparation of keto-ester S-8:</u>**

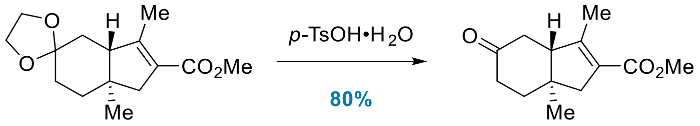


**26** **S-8**

To a stirred solution of ester **26** (6.12 g, 23.0 mmol, 1.0 equiv) in acetone (100 mL) at 25 °C was added $p$-TsOH·$H_2O$ (2.19 g, 11.5 mmol, 0.5 equiv). The reaction was stirred at 25 °C for 2.5 h before it was quenched with $NaHCO_3$ (80 mL, sat. aq.). The layers were separated, and the aqueous layer was extracted with EtOAc (3 × 80 mL). The combined organic layers were dried ($Na_2SO_4$) and concentrated in vacuo. Flash column chromatography (silica gel, PE:EtOAc 20:1) afforded keto-ester **S-8** (4.09 g,

80%) as a yellow oil. **S-8**: $R_f$ = 0.43 (silica gel, PE:EtOAc 10:1); $[\alpha]_D^{20}$ = –13.2 (c = 1.0, $CHCl_3$); $^1H$ NMR (400 MHz, $CDCl_3$) δ = 3.73 (s, 3 H), 2.69 (br d, $J$ = 17.7 Hz, 1 H), 2.56 (dd, $J$ =15.6, 4.2 Hz, 1 H), 2.51–2.33 (m, 4 H), 2.30 (dt, $J$ = 14.7, 3.1 Hz, 1 H), 2.14–1.98 (m, 3 H), 1.98–1.81 (m, 2 H), 1.03 (s, 3 H); $^{13}C$ NMR (100 MHz, $CDCl_3$) δ = 210.5, 166.8, 155.2, 128.4, 56.5, 51.2, 45.4, 42.4, 39.1, 38.2, 34.6, 18.0, 14.0 ppm; HRMS (ESI): calcd for $C_{13}H_{18}NaO_3^+$ $[M + Na]^+$ 245.1148, found 245.1149.

**Preparation of diol 27:**

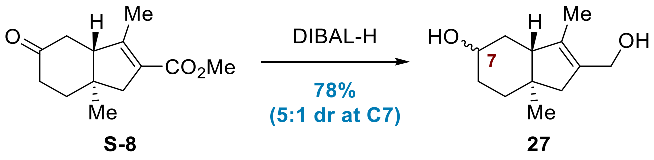


To a stirred solution of keto-ester **S-8** (4.89 g, 22.0 mmol, 1.0 equiv) in toluene (60 mL) at –78 °C was added DIBAL-H (58.7 mL, 1.5 M in toluene, 4.0 equiv). The reaction was stirred at –78 °C for 1 h before it was quenched with Rochelle salt (80 mL, sat. aq.). The layers were separated, and the aqueous layer was extracted with EtOAc (3 × 80 mL). The combined organic layers were dried ($Na_2SO_4$) and concentrated in vacuo. Flash column chromatography (silica gel, PE:EtOAc 1:1) afforded diol **27** (3.37 g, 78%, 5:1 dr at C7) as a colorless oil. **27**: $R_f$ = 0.25 (silica gel, PE:EtOAc 1:1); $[\alpha]_D^{20}$ = –23.5 (c = 1.0, $CHCl_3$); $^1H$ NMR (400 MHz, $CDCl_3$) δ = 4.14 (q, $J$ = 11.9 Hz, 2 H), 3.74–3.61 (m, 0.83 H), 2.63 (d, $J$ = 14.2 Hz, 0.17 H), 2.16–1.92 (m, 4 H), 1.80–1.64 (m, 3 H), 1.60 (br s, 3 H), 1.55–1.49 (m, 1 H), 1.44–1.30 (m, 1 H), 0.85 (s, 2.5 H), 0.78 ppm (s, 0.5 H); $^{13}C$ NMR (100 MHz, $CDCl_3$) δ = 137.5, 136.9, 134.5, 133.7, 72.2, 66.7, 59.5, 55.0 (2C), 49.1, 47.4, 46.6, 43.2, 43.0, 34.8, 32.2, 31.7, 31.5, 29.9, 29.6, 18.6,

17.3, 11.7 (2C) ppm; HRMS (ESI): calcd for $C_{12}H_{20}NaO_2^+$ $[M + Na]^+$ 219.1356, found 219.1358.

**Preparation of ketone 28:**

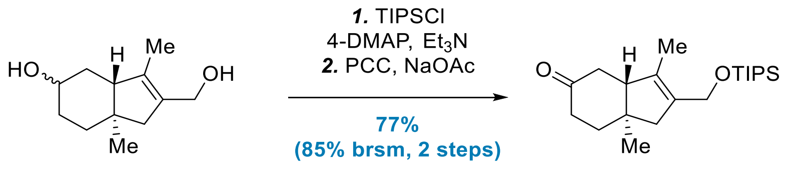


To a stirred solution of diol **27** (4.63 g, 23.6 mmol, 1.0 equiv) in $CH_2Cl_2$ (50 mL) at 25 °C were sequentially added $Et_3N$ (6.56 mL, 47.2 mmol, 2.0 equiv), 4-DMAP (2.88 g, 23.6 mmol, 1.0 equiv) and TIPSCl (5.05 mL, 23.6 mmol, 1.0 equiv). The resulting mixture was stirred at 25 °C for 2.5 h before it was quenched with $NaHCO_3$ (50 mL, sat. aq.). The layers were separated, and the aqueous layer was extracted with $CH_2Cl_2$ (3 × 50 mL). The combined organic layers were dried ($Na_2SO_4$) and concentrated in vacuo. Flash column chromatography (silica gel, PE:EtOAc 10:1 to 2:1) afforded a mixture of silyl-ethers (7.32 g, 88%) as a colorless oil, along with recovered **27** (415 mg, 9%).

To a stirred solution of the silyl-ethers (obtained above, 7.32 g, 20.8 mmol, 1.0 equiv) in $CH_2Cl_2$ (80 mL) at 25 °C were sequentially added NaOAc (8.51 g, 0.104 mol, 5.0 equiv) and PCC (8.95 g, 41.5 mmol, 2.0 equiv). The reaction was stirred at 25 °C for 1 h before it was added $Et_2O$ (50 mL). The resulting mixture was filtered through a

short pad of celite, and the filtrate was extracted with MTBE (3 × 50 mL). The combined organic layers were quenched with $NaHCO_3$ (100 mL, sat, aq.). The layers were separated, and the aqueous layer was extracted with EtOAc (3 × 100 mL). The combined organic layers were dried ($Na_2SO_4$) and concentrated in vacuo. Flash column chromatography (silica gel, PE:EtOAc 20:1) afforded ketone **28** (6.35 g, 87%) as a colorless oil. **28**: $R_f$ = 0.5 (silica gel, PE:EtOAc 10:1); $[\alpha]_D^{20}$ = –0.3 (c = 1.0, $CHCl_3$); $^1H$ NMR (400 MHz, $CDCl_3$) δ = 4.55–4.07 (m, 2 H), 2.63 (d, $J$ = 14.8 Hz, 1 H), 2.56–2.16 (m, 5 H), 2.10 (d, $J$ = 14.3 Hz, 1 H), 1.96–1.76 (m, 2 H), 1.56 (s, 3 H), 1.26–1.04 (m, 21 H), 1.01 ppm (s, 3 H); $^{13}C$ NMR (100 MHz, $CDCl_3$) δ = 212.1, 136.7, 132.4, 60.2, 55.1, 46.1, 42.3, 39.8, 38.5, 34.9, 18.2 (6C), 17.8, 12.2 (3C), 11.6 ppm; HRMS (ESI): calcd for $C_{21}H_{38}NaO_2Si^+$ $[M + Na]^+$ 373.2533, found 373.2533.

**Preparation of *α*-ketol S-9:**

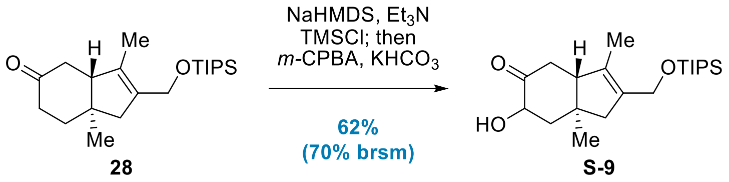


To a stirred solution of ketone **28** (4.20 g, 12.0 mmol, 1.0 equiv) in THF (100 mL) at –78 °C was added NaHMDS (60.0 mL, 2.0 M in THF, 10 equiv). The resulting mixture was stirred at –78 °C for 1 h before it was sequentially added $Et_3N$ (3.33 mL, 24.0 mmol, 2.0 equiv) and TMSCl (3.04 mL, 24.0 mmol, 2.0 equiv). The reaction was warmed to 50 °C and stirred for an additional 1 h before it was cooled to 0 °C and quenched with $NaHCO_3$ (100 mL, sat. aq.). The layers were separated, and the aqueous layer was extracted with EtOAc (3 × 100 mL). The combined organic layers were dried

($Na_2SO_4$) and concentrated in vacuo to afford the crude silyl enol ether as a yellow oil, which was used directly without further purification.

To a stirred solution of the silyl enol ether (crude, obtain above) in $CH_2Cl_2$ (150 mL) at 0 °C were sequentially added $KHCO_3$ (12.0 g, 120 mmol, 10 equiv) and *m*-CPBA (2.92 g, 85 wt. %, 1.2 equiv). The reaction was stirred at 0 °C for 10 min before it was quenched with $NaHCO_3$ (100 mL, sat. aq.). The layers were separated, and the aqueous layer was extracted with $CH_2Cl_2$ (3 × 100 mL). The combined organic layers were sequentially washed with HCl (200 mL, 2.0 M in $H_2O$) and brine (200 mL, sat. aq.), dried ($Na_2SO_4$) and concentrated in vacuo. Flash column chromatography (silica gel, PE:EtOAc 10:1 to 5:1) afforded *α*-ketol **S-9** (2.72 g, 62%) as a yellow oil, along with recovered **28** (462 mg, 11%). **S-9**: $R_f$ = 0.35 (silica gel, PE:EtOAc 5:1); $[\alpha]_D^{20}$ = –76.2 (c = 1.0, $CHCl_3$); $^1H$ NMR (400 MHz, $CDCl_3$) δ = 4.32–4.19 (m, 3 H), 3.60 (s, 1 H), 2.68–2.43 (m, 4 H), 2.24 (d, *J* = 14.2 Hz, 1 H), 2.09 (d, *J* = 14.4 Hz, 1 H), 1.70 (t, *J* = 11.7 Hz, 1 H), 1.58 (s, 3 H), 1.14 (s, 3 H), 1.10–1.02 ppm (m, 21 H); $^{13}C$ NMR (100 MHz, $CDCl_3$) δ = 211.2, 137.1, 132.0, 73.8, 60.1, 57.0, 45.6, 44.9, 43.4, 37.9, 19.3 (6C), 18.1, 12.1 (3C), 11.7 ppm; HRMS (ESI): calcd for $C_{21}H_{38}NaO_3Si^+$ $[M + Na]^+$ 389.2488, found 389.2484.

**Preparation of aldehyde 30:**

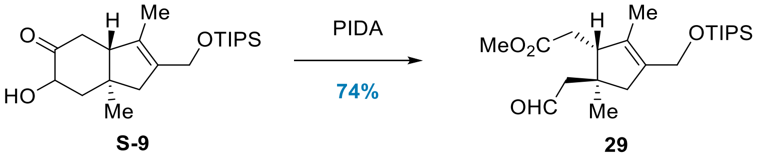


To a stirred solution of *α*-ketol **S-9** (3.08 g, 8.40 mmol, 1.0 equiv) in MeOH (100 mL) at 25 °C was added PIDA (5.42 g, 16.8 mmol, 2.0 equiv). The reaction was stirred

at 25 °C for 2 h before it was quenched with $Na_2S_2O_3$ (100 mL, sat. aq.). The layers were separated, and the aqueous layer was extracted with EtOAc (3 × 100 mL). The combined organic layers were dried ($Na_2SO_4$) and concentrated in vacuo. Flash column chromatography (silica gel, PE:EtOAc 8:1) afforded aldehyde **29** (2.46 g, 74%) as a yellow oil. **30**: $R_f$ = 0.45 (silica gel, PE:EtOAc 5:1); $[\alpha]_D^{20}$ = –5.5 (c = 1.0, $CHCl_3$); $^1H$ NMR (400 MHz, $CDCl_3$) δ = 9.71 (t, $J$ = 3.1 Hz, 1 H), 4.21 (s, 2 H), 3.68 (s, 3 H), 2.81 (t, $J$ = 7.3 Hz, 1 H), 2.45–2.31 (m, 6 H), 1.55 (s, 3 H), 1.08–1.03 ppm (m, 24 H); $^{13}C$ NMR (100 MHz, $CDCl_3$) δ = 202.7, 173.9, 135.6, 133.9, 59.8, 56.0, 54.4, 51.8, 45.8, 42.7, 33.7, 22.5, 18.1 (6C), 12.4, 12.1 (3C) ppm; HRMS (ESI): calcd for $C_{22}H_{40}NaO_4Si^+$ $[M + Na]^+$ 419.2588, found 419.2598.

**Preparation of acetal alcohol 30:**

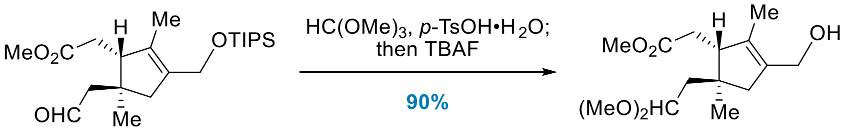


**29** **30**

To a stirred solution of aldehyde **29** (2.73 g, 6.89 mmol, 1.0 equiv) in MeOH (350 mL) at 25 °C were sequentially added $CH(OMe)_3$ (7.53 mL, 68.8 mmol, 10 equiv) and $p$-TsOH·$H_2O$ (393 mg, 2.07 mmol, 0.3 equiv). The reaction was stirred at 25 °C for 10 min before it was quenched with $NaHCO_3$ (200 mL, sat. aq.). The layers were separated, and the aqueous layer was extracted with EtOAc (3 × 150 mL). The combined organic layers were dried ($Na_2SO_4$) and concentrated in vacuo to afford crude acetal as a yellow oil, which was used directly without further purification.

To a stirred solution of the acetal (crude, obtain above) in THF (50 mL) at 25 °C was added TBAF (13.8 mL, 1.0 M in THF, 2.0 equiv). The reaction was stirred at 25 °C for 1 h before it was quenched with $NH_4Cl$ (50 mL, sat. aq.). The layers were separated, and the aqueous layer was extracted with EtOAc (3 × 50 mL). The combined organic layers were dried ($Na_2SO_4$) and concentrated in vacuo. Flash column chromatography (silica gel, silica gel, PE:EtOAc 2:1) afforded acetal alcohol **30** (1.78 g, 90%) as a yellow oil. **S-9**: $R_f$ = 0.50 (silica gel, PE:EtOAc 1:1); $[\alpha]_D^{20}$ = –6.7 (c = 1.0, $CHCl_3$); $^1H$ NMR (400 MHz, $CDCl_3$) δ = 4.45 (t, $J$ = 5.1 Hz, 1 H), 4.13 (q, $J$ = 12.1 Hz, 2 H), 3.68 (s, 3 H), 3.28 (d, $J$ = 6.7 Hz, 6 H), 2.80 (t, $J$ = 7.3 Hz, 1 H), 2.40–2.15 (m, 4 H), 1.75–1.64 (m, 3 H), 1.60 (s, 3 H), 0.95 ppm (s, 3 H); $^{13}C$ NMR (100 MHz, $CDCl_3$) δ = 174.1, 136.0, 134.0, 102.7, 59.1, 54.2, 52.8, 51.9, 51.8, 45.8, 44.1, 41.6, 33.4, 22.1, 12.3 ppm; HRMS (ESI): calcd for $C_{15}H_{26}NaO_5^+$ $[M + Na]^+$ 309.1672, found 309.1677.

**Preparation of acetal aldehyde 14:**

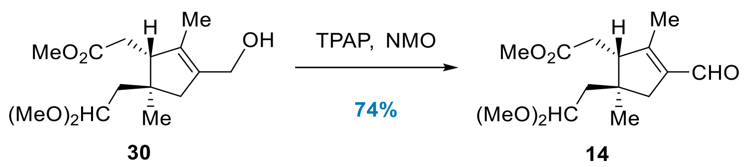


To a stirred solution of acetal alcohol **S-9** (2.63 g, 9.20 mmol, 1.0 equiv) in $CH_2Cl_2$ (50 mL) at 25 °C were sequentially added NMO (1.61 g, 13.8 mmol, 1.5 equiv) and TPAP (484 mg, 1.38 mmol, 0.15 equiv). The reaction was stirred at 25 °C for 0.5 h before it was filtered through a short pad of celite, and the filtrate was extracted with $Et_2O$ (3 × 100 mL). The combined organic layers were concentrated in vacuo. Flash column chromatography (silica gel, PE:EtOAc 5:1) afforded acetal aldehyde **14** (1.93

g, 74%) as a yellow oil. **14**: $R_f$ = 0.55 (silica gel, PE:EtOAc 2:1); $[\alpha]_D^{20}$ = –43.9 (c = 1.0, $CHCl_3$); $^1$H NMR (400 MHz, acetone-$d_6$) δ = 10.02 (s, 1 H), 4.45 (t, *J* = 5.2 Hz, 1 H), 3.68 (s, 3 H), 3.25 (d, *J* = 2.2 Hz, 6 H), 3.15–3.06 (m, 1 H), 2.50–2.38 (m, 3 H), 2.26 (dt, *J* = 15.8, 1.7 Hz, 1 H), 2.08 (d, *J* = 1.8 Hz, 3 H), 1.77–1.62 (m, 2 H), 0.93 ppm (s, 3 H); $^{13}$C NMR (100 MHz, acetone-$d_6$) δ = 188.7, 173.9, 161.8, 137.7, 103.5, 56.3, 52.8, 52.5, 51.9, 44.2, 42.9, 42.5, 32.4, 21.9, 12.6 ppm; HRMS (ESI): calcd for $C_{15}H_{24}NaO_5^+$ $[M + Na]^+$ 307.1516, found 307.1527.

**Preparation of allyl alcohol S-10:**

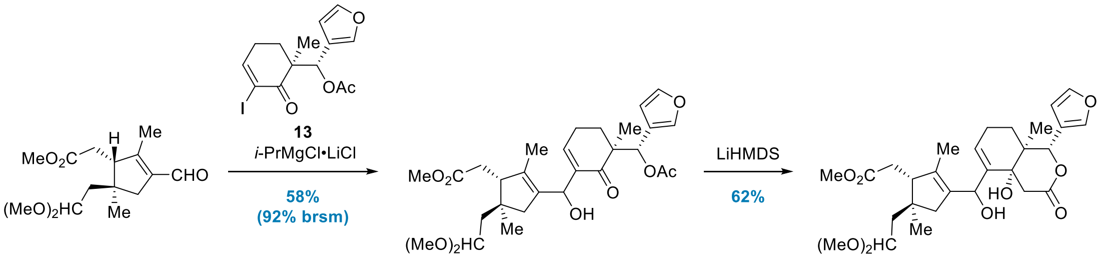


14 12 S-10

To a stirred solution of alkenyl iodide **13** (2.17 g, 5.80 mmol, 2.0 equiv) in toluene (60 mL) at –78 °C was added *i*-PrMgCl·LiCl (4.46 mL, 1.3 M in THF, 2.0 equiv). The resulting mixture was stirred at –78 °C for 5 min before it was added a solution of **14** (824 mg, 2.90 mmol, 1.0 equiv) in toluene (5 mL). The reaction was stirred at –78 °C for an additional 10 min before it was quenched with $H_2O$ (1 mL). The resulting mixture was filtered through a short pad of celite, and the filtrate was extracted with EtOAc (3 × 50 mL). The combined organinc layers were concentrated in vacuo. Flash column chromatography (silica gel, PE:EtOAc 10:1 to 1:2) afforded crude allyl alcohol **12** (895 mg, 58%) as a yellow oil, along with recovered **14** (239 mg, 29%). **12** was unstable, and it was used without further purification.

To a stirred solution of **12** (obtained above, 895 mg, 1.68 mmol, 1.0 equiv) in THF (20 mL) at –78 °C was added LiHMDS (6.72 mL, 1.0 M in THF, 4.0 equiv). The reaction was stirred at –78 °C for 0.5 h before it was quenched with $NH_4Cl$ (15 mL, sat. aq.). The layers were separated, and the aqueous layer was extracted with EtOAc (3 × 20 mL). The combined organic layers were dried ($Na_2SO_4$) and concentrated in vacuo. Flash column chromatography (silica gel, PE:EtOAc 1:1) afforded allyl alcohol **S-10** (556 mg, 62%) as a yellow oil. **S-10**: $R_f$ = 0.40 (silica gel, PE:EtOAc 1:2); $[\alpha]_D^{20}$ = –17.2 (c = 1.0, $CHCl_3$); $^1$H NMR (400 MHz, $CDCl_3$) δ = 7.43 (s, 1 H), 7.40 (s, 1 H), 6.42 (s, 1 H), 5.77 (br s, 1 H), 5.21 (s, 1 H), 5.19 (s, 1 H), 4.39 (t, *J* = 4.8 Hz, 1 H), 3.89 (br s, 1 H), 3.65 (s, 3 H), 3.57 (br s, 1 H), 3.27 (s, 3 H), 3.22 (s, 3 H), 3.13 (d, *J* = 17.8 Hz, 1 H), 2.91 (d, *J* = 17.8 Hz, 1 H), 2.70 (t, *J* = 6.9 Hz, 1 H), 2.38–2.23 (m, 3 H), 2.13 (br s, 2 H), 2.05–1.97 (m, 1 H), 1.85–1.78 (m, 1 H), 1.69 (dd, *J* = 14.3, 5.1 Hz, 1 H), 1.65–1.58 (s, 4 H), 1.36–1.30 (m, 1 H), 0.99 (s, 3 H), 0.94 ppm (s, 3 H); $^{13}$C NMR (100 MHz, $CDCl_3$) δ = 174.0, 170.7, 143.1, 140.7, 138.8, 137.9, 134.5, 126.2, 121.3, 110.0, 102.3, 77.9, 71.6, 67.9, 55.0, 53.5, 51.8, 50.7, 44.3, 42.3, 41.4, 40.5, 39.1, 33.3, 27.2, 22.6, 21.9, 15.3, 12.8 ppm; HRMS (ESI): calcd for $C_{29}H_{40}NaO_9^+$ $[M + Na]^+$555.2565, found 555.2589.

**Preparation of dienone 11:**

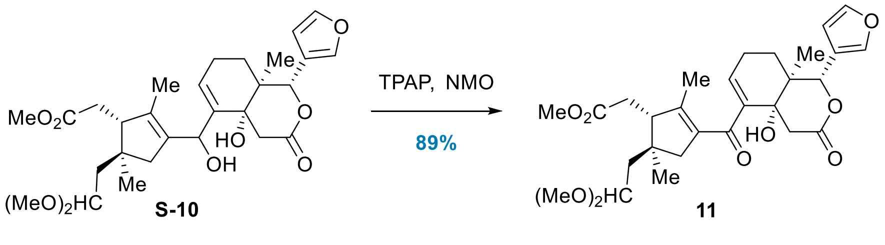

To a stirred solution of allyl alcohol **S-10** (463 mg, 0.870 mmol, 1.0 equiv) in $CH_2Cl_2$ (20 mL) at 0 °C were sequentially added NMO (153 mg, 1.30 mmol, 1.5 equiv) and TPAP (61.1 mg, 0.174 mmol, 0.2 equiv). The reaction was warmed to 25 °C and stirred for 0.5 h. The resulting mixture was filtered through a short pad of celite, and the filtrate was extracted with $Et_2O$ (3 × 50 mL). The combined organic layers were concentrated in vacuo. Flash column chromatography (silica gel, PE:EtOAc 3:1) afforded dienone **11** (411 mg, 89%) as a yellow oil. **11**: $R_f$ = 0.55 (silica gel, PE:EtOAc 1:1); $[\alpha]_D^{20}$ = +22.5 (c = 1.0, $CHCl_3$); $^1H$ NMR (400 MHz, $CDCl_3$) δ = 7.45 (br s, 1 H), 7.40 (t, $J$ = 1.7 Hz, 1 H), 6.68 (t, $J$ = 4.0 Hz, 1 H), 6.44 (br s, 1 H), 5.16 (s, 1 H), 4.77 (s, 1 H), 4.44 (t, $J$ = 5.2 Hz, 1 H), 3.66 (s, 3 H), 3.27 (s, 6 H), 3.05–2.92 (m, 3 H), 2.70 (dt, $J$ = 15.6, 2.5 Hz, 1 H), 2.44–2.30 (m, 4 H), 2.20 (dt, $J$ = 15.4, 1.6 Hz, 1 H), 1.85–1.79 (m, 1 H), 1.71 (br s, 5 H), 1.44 (dt, $J$ = 13.8, 5.4 Hz, 1 H), 0.99 (s, 3 H), 0.91 ppm (s, 3 H); $^{13}C$ NMR (100 MHz, $CDCl_3$) δ = 199.7, 173.3, 170.0, 151.2, 143.2, 142.7, 141.8, 140.8, 135.2, 120.8, 109.9, 102.6, 78.0, 71.3, 55.2, 52.9, 52.5, 51.9, 47.1, 43.1, 42.1, 41.1, 40.4, 32.2, 27.9, 22.8, 21.6, 15.5, 15.1 ppm; HRMS (ESI): calcd for $C_{29}H_{38}NaO_9^+$ $[M + Na]^+$ 553.2408, found 553.2418.

**Preparation of enone 10:**

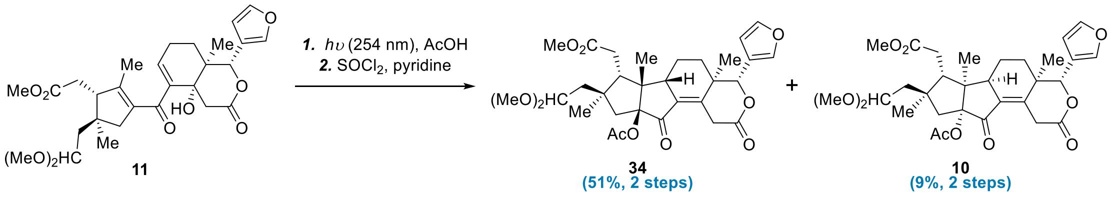


A solution of dienone **11** (7.95 mg, 15 μmol, 1.0 equiv) and AcOH (1.72 μL, 0.03 mmol, 2.0 equiv) in DCE (5.0 mL) was added into a quartz tube and purged with argon

gas for 5 min. The resulting mixture was stirred at 20 °C and irradiated with a 254 nm lamp (500 W) for 35 min before it was quenched with $NaHCO_3$ (10 mL, sat. aq.). The layers were separated, and the aqueous layer was extracted with $CH_2Cl_2$ (3 × 10 mL). The combined organic layers were dried ($Na_2SO_4$) and concentrated in vacuo. Flash column chromatography (silica gel, PE:EtOAc 1:1) afforded a mixture of ketones (6.29 mg, 71%) as a colorless oil.

To a stirred solution of the ketones (obtained above, 6.29 mg, 10.7 μmol, 1.0 equiv) in $CH_2Cl_2$ (5 mL) at 0 °C were sequentially added pyridine (4.29 μL, 53.3 μmol, 5.0 equiv) and $SOCl_2$ (2.32 μL, 32.0 μmol, 3.0 equiv). The reaction was stirred at 0 °C for 0.5 h before it was quenched with $NaHCO_3$ (10 mL, sat. aq.). The layers were separated, and the aqueous layer was extracted with $CH_2Cl_2$ (3 × 10 mL). The combined organic layers were dried ($Na_2SO_4$) and concentrated in vacuo. Flash column chromatography (silica gel, PE:EtOAc 2:1) afforded enone **10** (0.77 mg, 9% over two steps) as a white solid, along with enone **34** (4.38 mg, 51% over two steps) as a colorless oil.

**10**: m.p. 63–65 °C ($Et_2O$/PE); $R_f$ = 0.55 (silica gel, PE:EtOAc 1:1); $[\alpha]_D^{20}$ = –13.0 (c = 0.5, $CHCl_3$); $^1H$ NMR (400 MHz, $CDCl_3$) δ = 7.53 (br s, 1 H), 7.45 (t, *J* = 1.8 Hz, 1 H), 6.45 (br s, 1 H), 5.41 (s, 1 H), 4.50 (d, *J* = 21.3 Hz, 1 H), 4.38 (t, *J* = 5.2 Hz, 1 H), 3.70 (s, 3 H), 3.66 (d, *J* = 5.1 Hz, 1 H), 3.26 (s, 3 H), 3.23 (s, 3 H), 2.81–2.72 (m, 1 H), 2.43 (dd, *J* = 15.9, 9.3 Hz, 1 H), 2.35 (dd, *J* = 16.0, 2.4 Hz, 1 H), 2.19–2.11 (m, 2 H), 2.11 (s, 3 H), 1.91 (d, *J* = 15.4 Hz, 1 H), 1.81–1.73 (m, 1 H), 1.73–1.60 (m, 2 H), 1.50–1.45 (m, 1 H), 1.44–1.33 (m, 2 H), 1.18 (s, 3 H), 1.10 (s, 3 H), 1.08 ppm (s, 3 H); $^{13}C$ NMR (100 MHz, $CDCl_3$) δ = 203.3, 173.7, 171.0, 168.4, 146.9, 143.5, 141.9, 131.7,

120.2, 109.8, 102.4, 92.5, 80.5, 53.3, 52.3, 52.2, 52.0, 51.9, 49.1, 48.7, 43.7, 42.5, 39.4, 33.8, 32.1, 32.0, 21.2, 20.5, 19.3, 19.0, 18.8 ppm; HRMS (ESI): calcd for $C_{31}H_{40}NaO_{10}^+$ $[M + Na]^+$ 595.2514, found 595.2513.

**34**: $R_f$ = 0.50 (silica gel, PE:EtOAc 1:1); $[\alpha]_D^{20}$ = –128.3 (c = 0.5, $CHCl_3$); $^1H$ NMR (400 MHz, $CDCl_3$) δ = 7.49 (br s, 1 H), 7.45 (t, $J$ = 1.9 Hz, 1 H), 6.43 (br s, 1 H), 5.18 (s, 1 H), 4.62 (d, $J$ = 21.8 Hz, 1 H), 4.34 (t, $J$ = 5.2 Hz, 1 H), 3.71 (s, 4 H), 3.29 (s, 3 H), 3.25 (s, 3 H), 3.04–2.98 (m, 1 H), 2.60 (dd, $J$ = 14.2, 3.0 Hz, 1 H), 2.52–2.42 (m, 2 H), 2.17 (d, $J$ = 3.8 Hz, 2 H), 2.09 (s, 3 H), 2.04–1.97 (m, 1 H), 1.89 (dt, $J$ = 14.6, 3.5 Hz, 1 H), 1.54–1.39 (m, 3 H), 1.37–1.30 (m, 1 H), 1.27 (s, 3 H), 1.15 (s, 3 H), 0.79 ppm (s, 3 H); $^{13}C$ NMR (100 MHz, $CDCl_3$) δ = 204.0, 173.7, 171.3, 168.3, 143.5, 142.8, 141.5, 134.5, 120.2, 110.0, 102.8, 92.8, 79.2, 60.3, 53.4, 52.4, 52.2, 51.7, 50.4, 49.8, 45.0, 40.0, 39.0, 33.5, 32.9, 32.1, 27.5, 23.2, 20.4, 20.2, 20.0 ppm; HRMS (ESI): calcd for $C_{31}H_{40}NaO_{10}^+$ $[M + Na]^+$ 595.2514, found 595.2518.

**Preparation of methyl ether 31:**

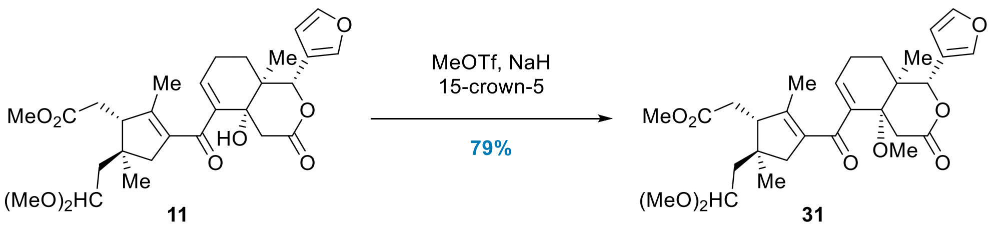


To a stirred solution of dienone **11** (10.0 mg, 18.9 μmol, 1.0 equiv) in THF (5 mL) at –20 °C were sequentially added NaH (7.54 mg, 60 wt. % in mineral oil, 10 equiv), MeOTf (6.83 μL, 66.0 μmol, 3.5 equiv) and 15-crown-5 (22.4 μL, 0.113 mmol, 6.0 equiv). The reaction was stirred at –20 °C for 1 h before it was quenched with $NH_4Cl$ (5 mL, sat. aq.). The layers were separated, and the aqueous layer was extracted with

EtOAc (3 × 5 mL). The combined organic layers were dried ($Na_2SO_4$) and concentrated in vacuo. Flash column chromatography (silica gel, PE:EtOAc 3:1) afforded methyl ether **31** (8.16 mg, 79%) as a colorless oil. $R_f$ = 0.42 (silica gel, PE:EtOAc 2:1); $[\alpha]_D^{20}$ = +29.0 (c = 0.5, $CHCl_3$); $^1$H NMR (400 MHz, acetone-$d_6$) δ = 7.70 (br s, 1 H), 7.60 (t, *J* = 1.8 Hz, 1 H), 7.06 (t, *J* = 3.9 Hz, 1 H), 6.57 (d, *J* = 1.4 Hz, 1 H), 5.36 (s, 1 H), 4.48 (t, *J* = 5.1 Hz, 1 H), 3.67 (s, 3 H), 3.59 (d, *J* = 17.6 Hz, 1 H), 3.26 (s, 3 H), 3.25 (s, 3 H), 3.24 (s, 3 H), 3.02–2.96 (m, 1 H), 2.90–2.83 (m, 1 H), 2.67 (dt, *J* = 15.5, 2.1 Hz, 1 H), 2.57–2.51 (m, 1 H), 2.49–2.38 (m, 3 H), 2.34 (dt, *J* = 15.5, 1.6 Hz, 1 H), 1.95 (ddd, *J* = 14.4, 10.9, 7.5 Hz, 1 H), 1.76 (s, 3 H), 1.72 (t, *J* = 5.3 Hz, 2 H), 1.36 (dd, *J* = 14.3, 6.6 Hz, 1 H), 1.04 (s, 3 H), 0.98 (s, 3 H); $^{13}$C NMR (100 MHz, acetone-$d_6$) δ = 197.5, 174.0, 169.2, 149.0, 148.0, 144.1, 142.1, 138.8, 136.7, 122.5, 110.9, 103.5, 77.5, 75.4, 55.8, 53.3, 52.9, 52.4, 51.9, 48.4, 44.3, 42.8, 42.0, 37.1, 32.9, 26.7, 23.6, 21.9, 16.1, 15.0 ppm; HRMS (ESI): calcd for $C_{30}H_{40}O_9Na^+$ $[M + Na]^+$ 567.2565, found 567.2571.

**<u>Preparation of enone 10:</u>**

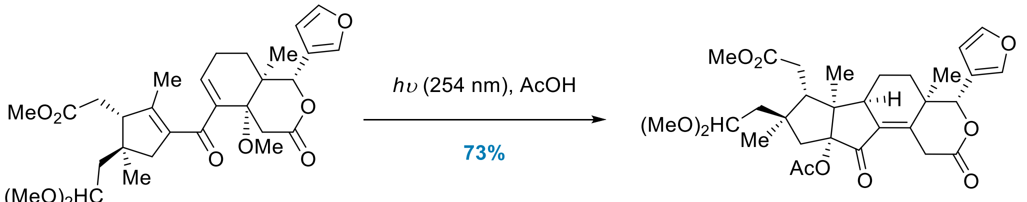


To a stirred solution of the dienone **31** (8.16 mg, 15.0 μmol, 1.0 equiv) and AcOH (1.72 μL, 0.03 mmol, 2.0 equiv) in $CH_2Cl_2$ (5.0 mL) was added into a quartz tube and purged with argon gas for 5 min. The resulting mixture was stirred at 20 °C and irradiated with a 254 nm lamp (500 W) for 35 min before it was quenched with $NaHCO_3$ (10 mL, sat. aq.). The layers were separated, and the aqueous layer was extracted with

$CH_2Cl_2$ (3 × 10 mL). The combined organic layers were dried ($Na_2SO_4$) and concentrated in vacuo. Flash column chromatography (silica gel, PE:EtOAc 1:1) afforded enone **10** (6.27 mg, 73%) as a white solid.

**III) Abbreviations**

| | |
|---|---|
| Ac | acetyl |
| DIBAL-H | diisobutylaluminium hydride |
| DTBMP | 2,6-di-*tert*-butyl-4-methylpyridine |
| 4-DMAP | 4-dimethylaminopyridine |
| HMDS | 1,1,1,3,3,3-hexamethyl-disilazan |
| IBX | 2-iodoxybenzoic acid |
| LDA | lithium diisopropylamide |
| *m*-CPBA | 3-chloroperoxybenzoic acid |
| MPO | 4-methoxypyridine *N*-oxide |
| NMO | 4-methylmorpholine *N*-oxide |
| PCC | pyridinium chlorochromate |
| PIDA | (diacetoxyiodo)benzene |
| Tf | trifluoromethanesulfonyl |
| TMS | trimethylsilyl |
| TIPS | triisopropylsilyl |
| TBAF | tetrabutylammonium fluoride |
| TPAP | tetrapropylammonium perruthenate |

## IV) References


1. Dolomanov, O. V.; Bourhis, L. J.; Gildea, R. J.; Howard, J. A. K.; Puschmann, H. *J. Appl. Cryst.* **2009**, *42*, 339–341. doi:10.1107/s0021889808042726
2. Marques, F. A.; Lenz, C. A.; Simonelli, F.; Maia, B. H. L. N. S.; Vellasco, A. P.; Eberlin, M. N. *J. Nat. Prod.* **2004**, *67*, 1939–1941. doi:10.1021/np049771x
3. He, C.; Bai, Z.; Hu, J.; Wang, B.; Xie, H.; Yu, L.; Ding, H. *Chem. Commun.* **2017**, *53*, 8435–8438. doi:10.1039/C7CC04292B
4. Birman, V. B.; Li, X. *Org. Lett.* **2006**, *8*, 1351–1354. doi:10.1021/ol060065s
5. Corey, E. J.; Huang, A. X. *J. Am. Chem. Soc.* 1999, **121**, 710-714. doi:10.1021/ja983179a
6. Hamphreys, D. J.; Newall, C. E.; Paskins, H. A.; Phillips, G. H. *J. Chem. Soc. Perkin Trans. 1,* **1978**, 15–19. doi:10.1039/p19780000015

## V) X-ray Crystallography of Compounds 10

**Table S2.** Crystal data and structure refinement for compound **10** (in the X ray structure of **10** solvent molecule is omitted for clarity).

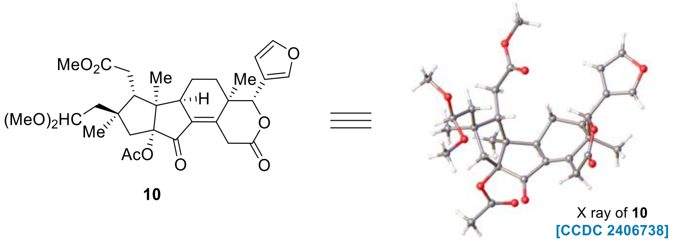


| | |
|---|---|
| Empirical Formula | $C_{33}H_{45}O_{10.5}$ |
| Formula weight | 609.69 |
| Temperature | 170.0 K |
| Crystal system | tetragonal |
| Space group | $P4_12_12$ |
| Unit cell dimensions | $a$ = 11.5324(2) Å, $\alpha$ =90°<br>$b$ = 11.5324(2) Å, $\beta$ = 90°<br>$c$ = 47.5262(13), $\gamma$= 90° |
| *Volume*/Å$^3$ | 6320.8(3) |
| $Z$ | 8 |
| $\rho_{calc}$g/g·cm$^{-3}$ | 1.281 |
| $\mu$/mm$^{-1}$ | 0.783 |
| $F$(000) | 2616.0 |
| Crystal size/mm$^3$ | 0.42 × 0.29 × 0.2 |
| Radiation | CuKα (λ = 1.54178) |
| 2$\theta$ range for data collection/° | 7.44 to 136.35 |
| Index ranges | -13 ≤ h ≤ 13, -13 ≤ k ≤ 12, -55 ≤ l ≤ 57 |
| Reflections collected | 77866 |
| Independent reflections | 5764 [$R_{int}$ = 0.0427, $R_{sigma}$ = 0.0189] |
| Data/restraints/parameters | 5764/54/424 |
| Goodness-of-fit on $F^2$ | 1.035 |
| Final $R$ indexes [$I$>=2$\sigma$ ($I$)] | $R_1$ = 0.0596, $wR_2$ = 0.1734 |
| Final $R$ indexes [all data] | $R_1$ = 0.0609, $wR_2$ = 0.1748 |
| Largest diff. peak/hole / e Å$^{-3}$ | 0.63/-0.37 |
| Flack parameter | 0.04(4) |

## VI) HPLC Chromatographs

Daicel Chiralcel AS-H (0.46 cm × 25 cm), *n*-hexane/2-propanol = 90/10, *v* = 1.0 mL·min$^{-1}$, λ = 210 nm.

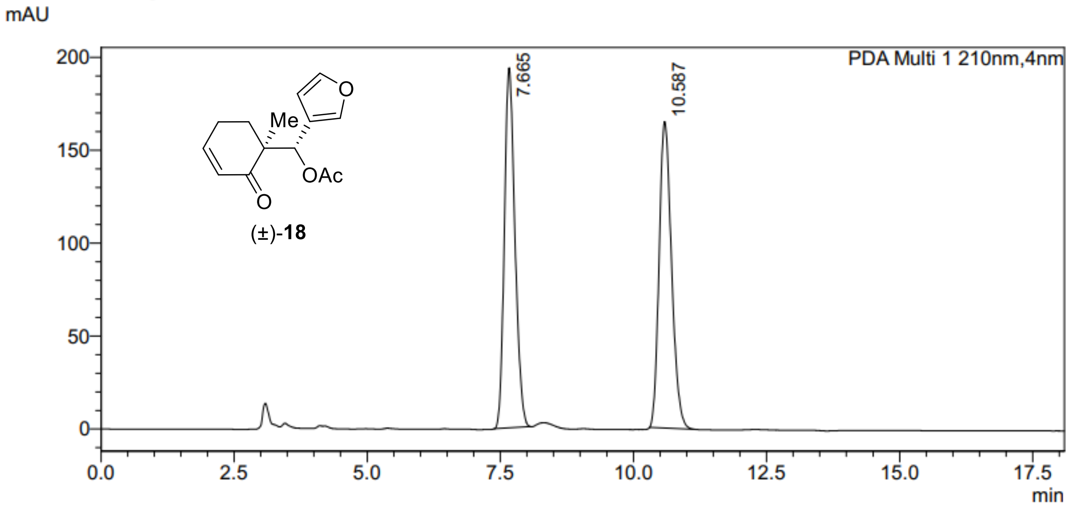


**<Peak Table>**

PDA Ch1 210nm

| Peak# | Ret. Time | Area | Area% |
|---|---|---|---|
| 1 | 7.665 | 2674764 | 50.060 |
| 2 | 10.587 | 2668342 | 49.940 |
| Total | | 5343105 | 100.000 |

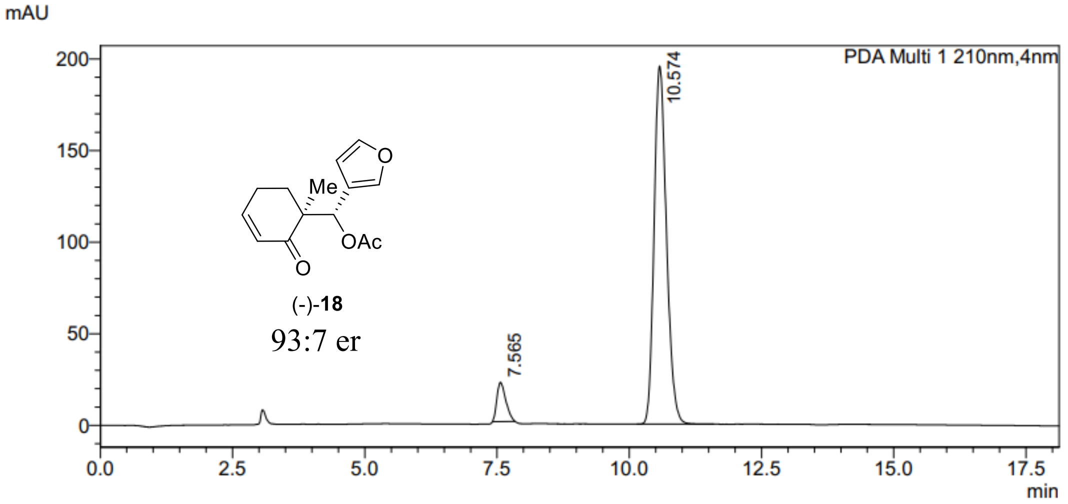


**<Peak Table>**

PDA Ch1 210nm

| Peak# | Ret. Time | Area | Area% |
|---|---|---|---|
| 1 | 7.565 | 251041 | 7.315 |
| 2 | 10.574 | 3180614 | 92.685 |
| Total | | 3431655 | 100.000 |

## VII) $^1$H and $^{13}$C NMR Spectra of Compounds

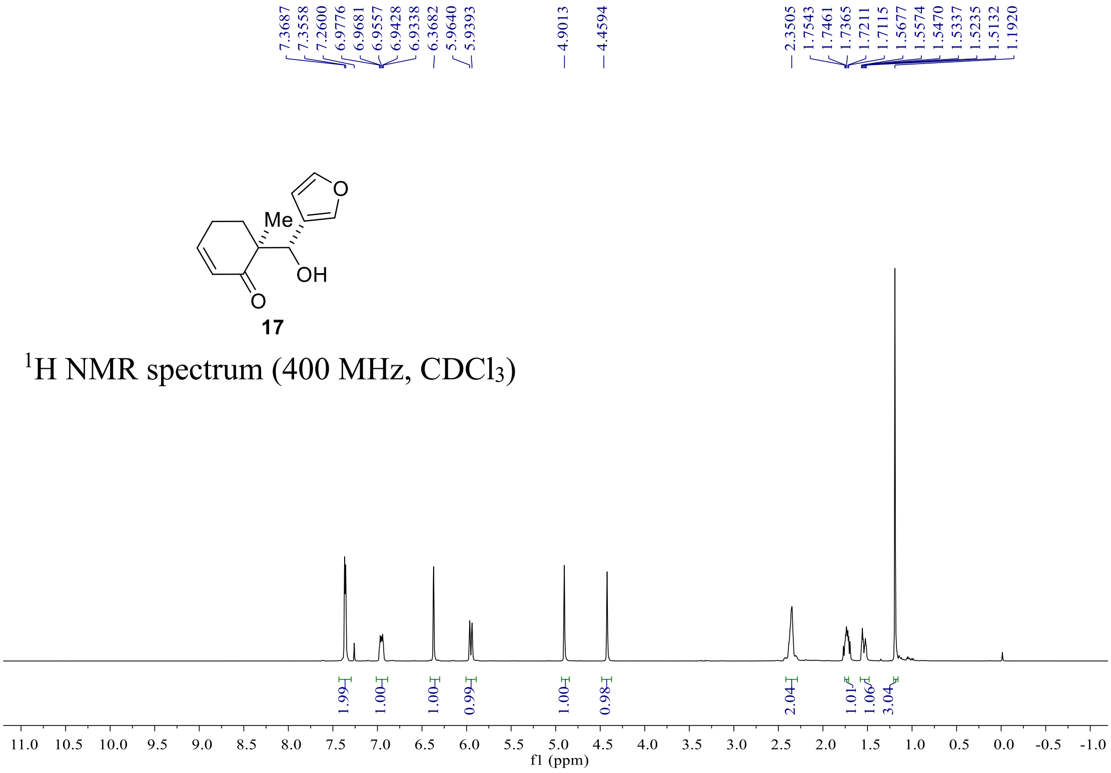


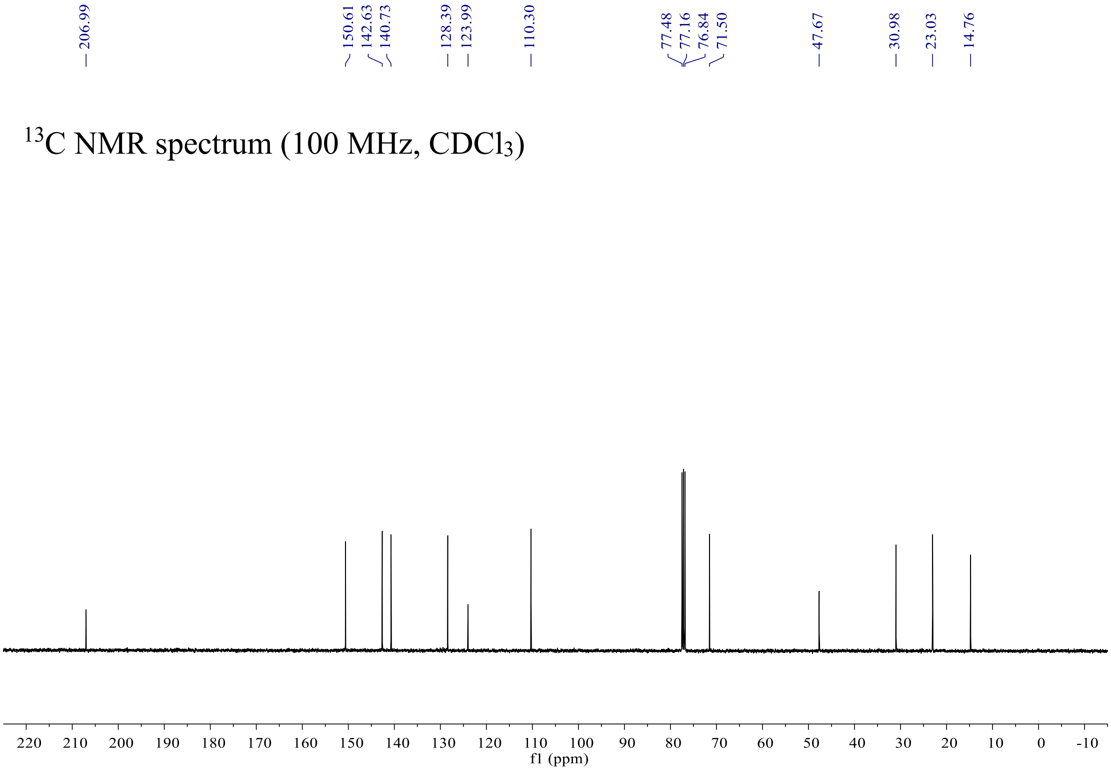

**18**

$^{1}$H NMR spectrum (400 MHz, $CDCl_3$)

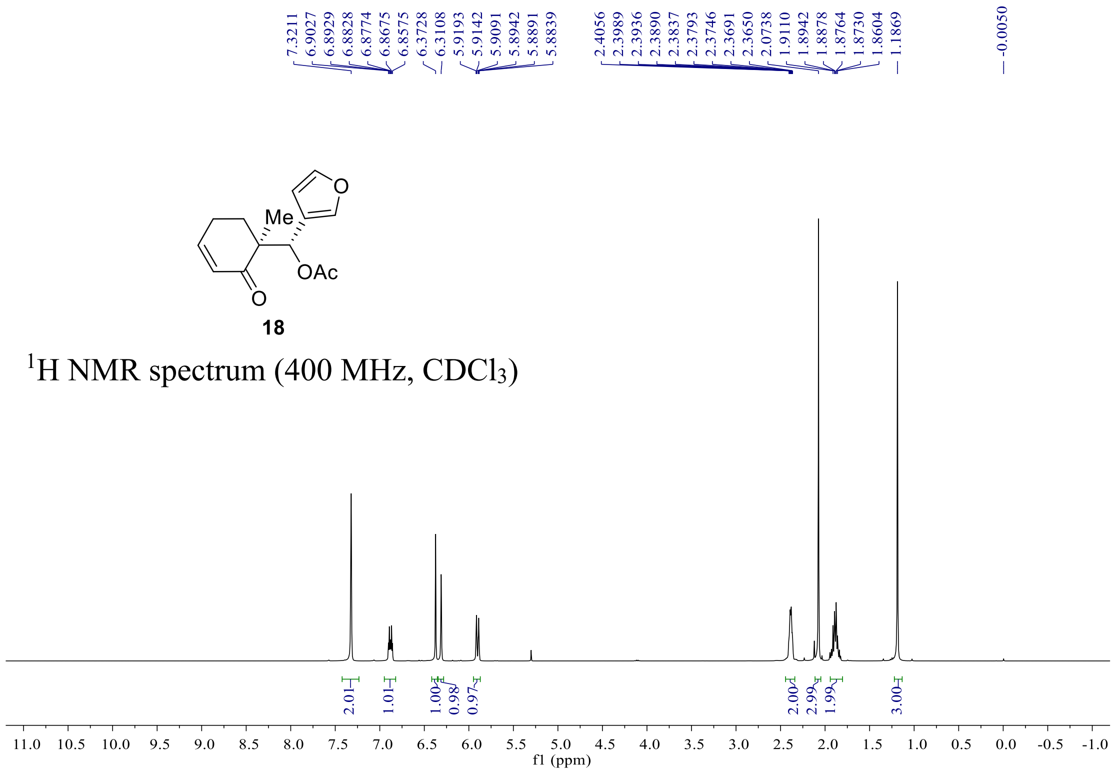


$^{13}$C NMR spectrum (100 MHz, $CDCl_3$)

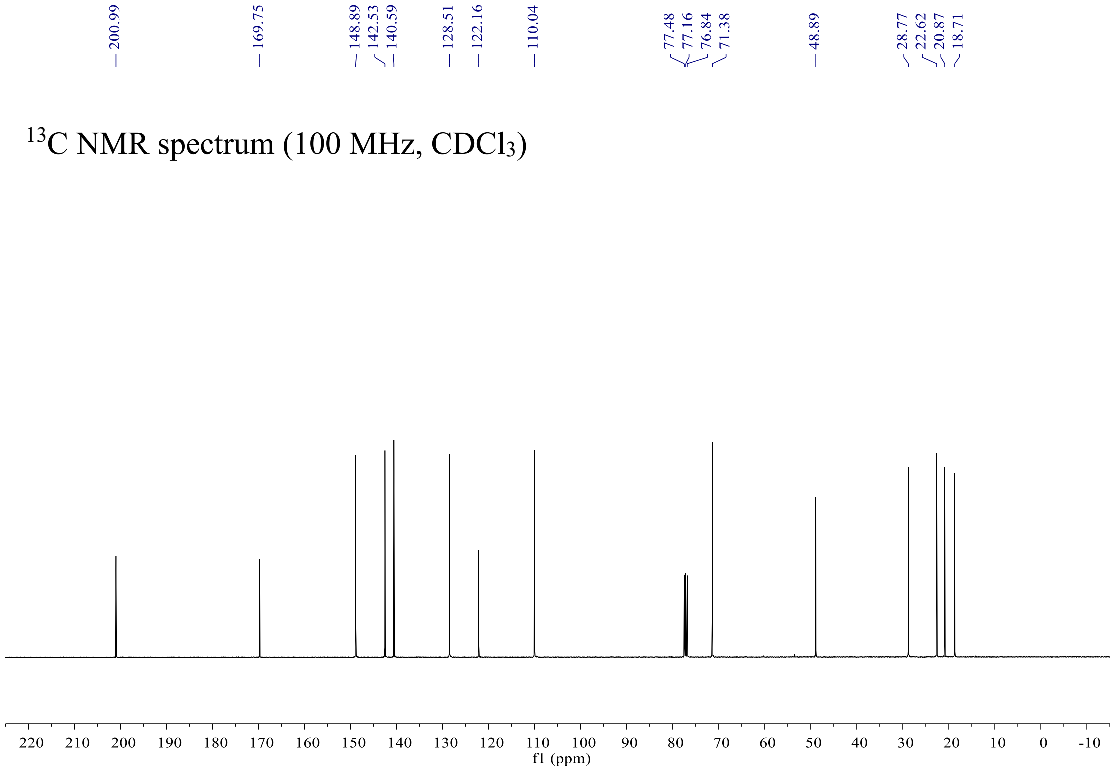

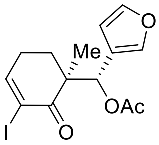


**13**

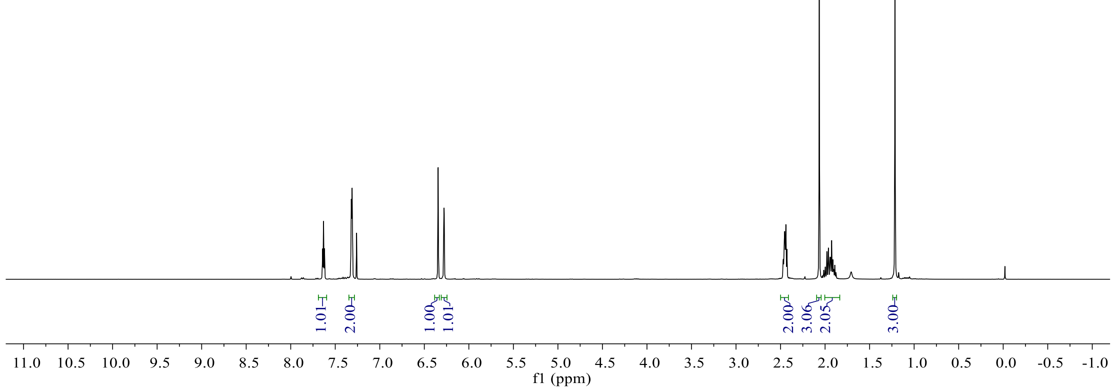


$^{1}$H NMR spectrum (400 MHz, $CDCl_3$)

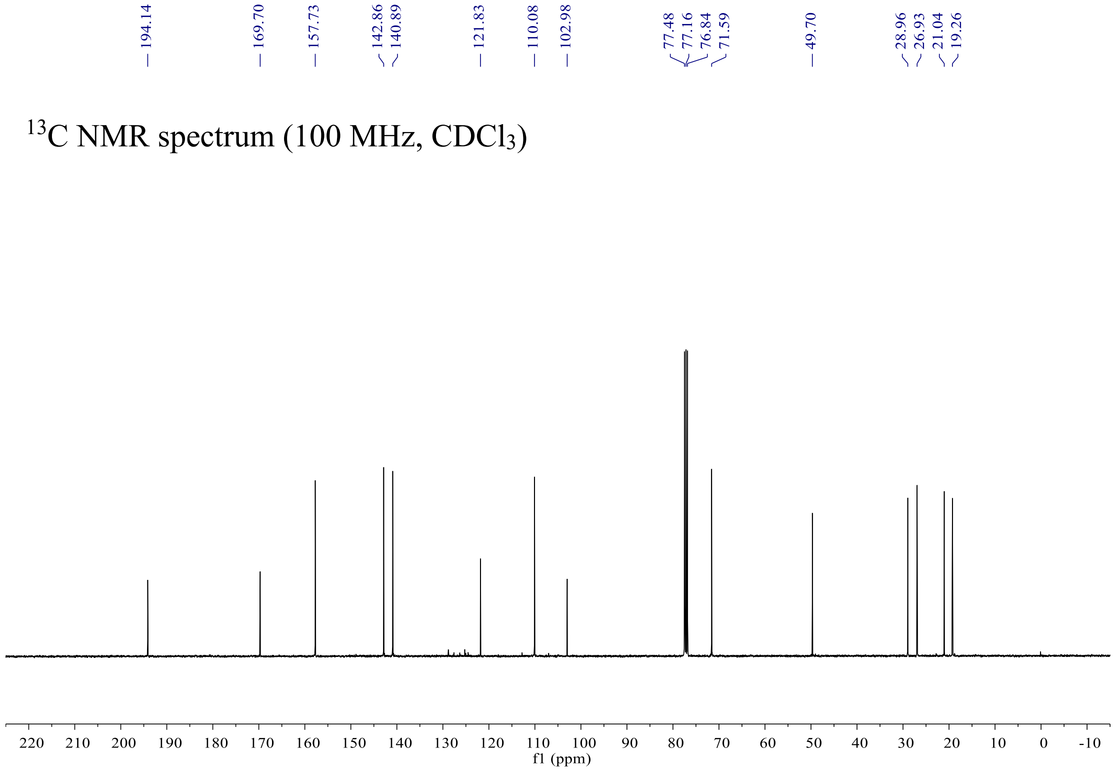


$^{13}$C NMR spectrum (100 MHz, $CDCl_3$)

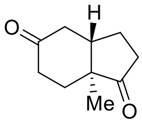


**S-1**

$^1$H NMR spectrum (400 MHz, $CDCl_3$)

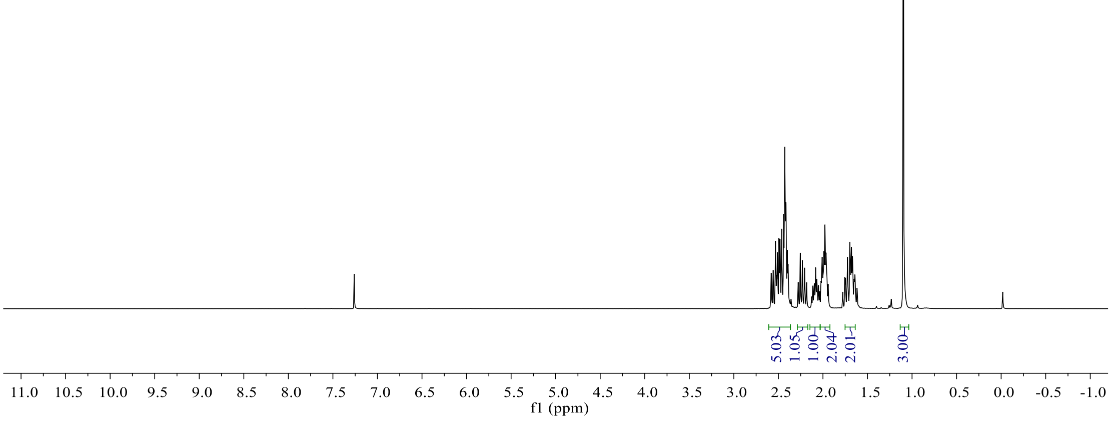

$^{13}$C NMR spectrum (100 MHz, $CDCl_3$)

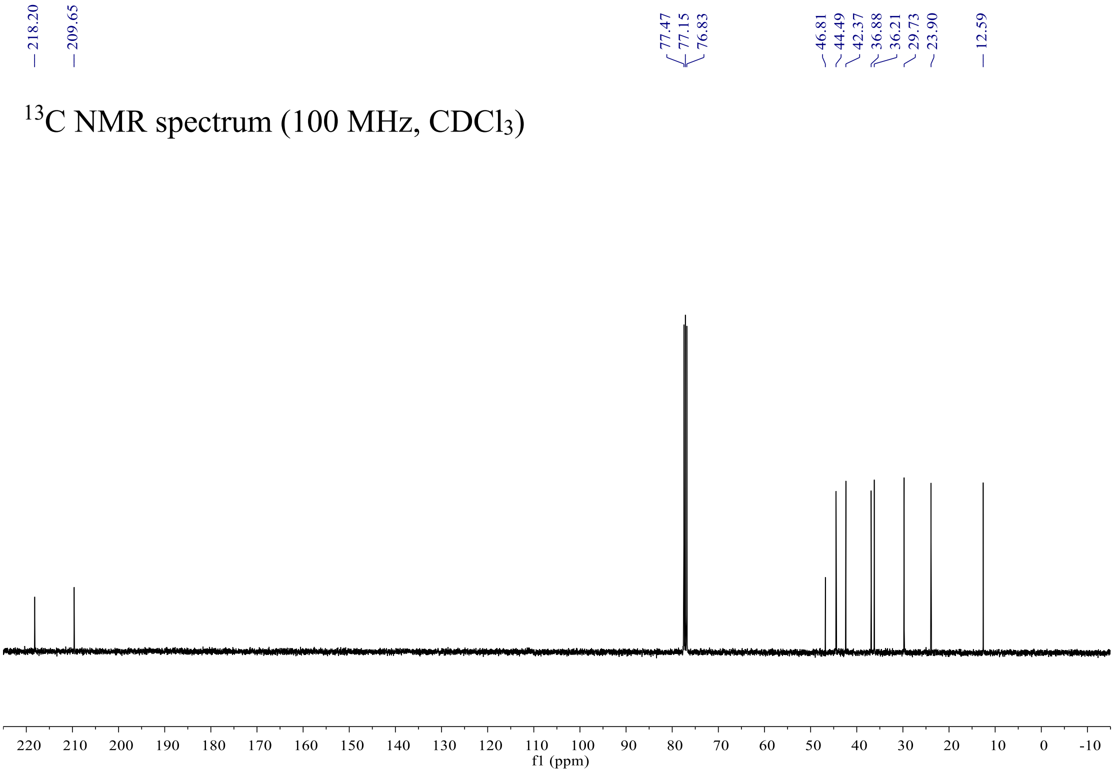

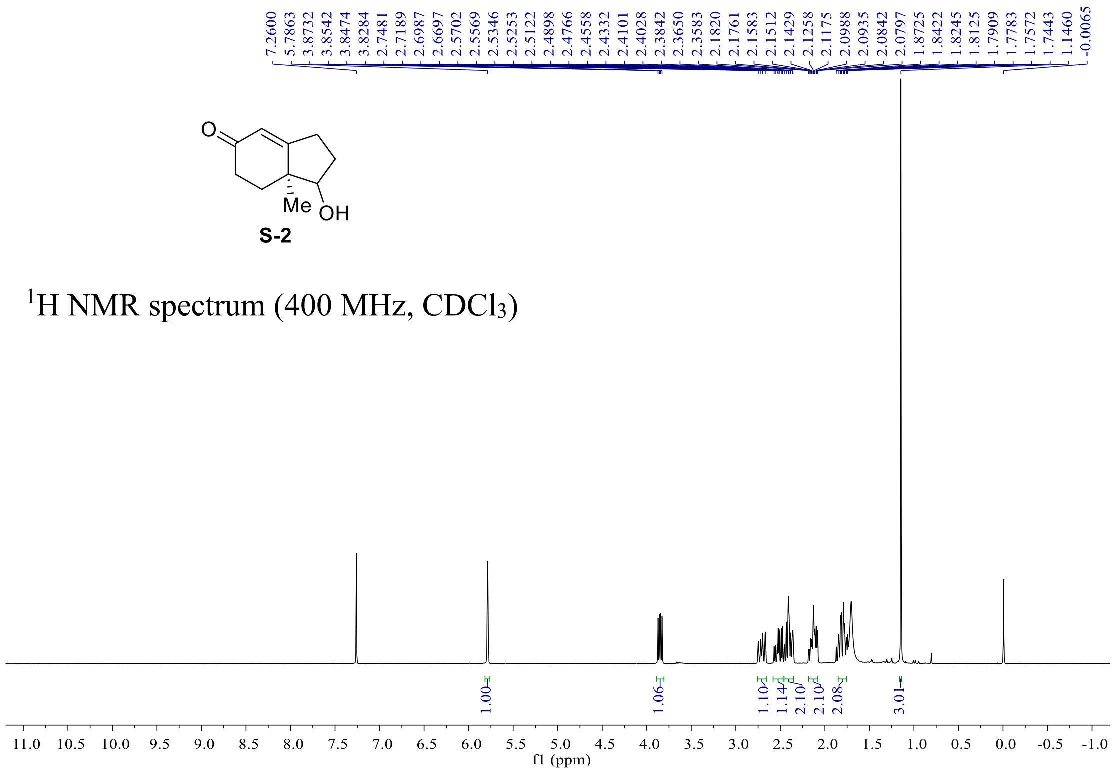


$^{1}$H NMR spectrum (400 MHz, $CDCl_3$)

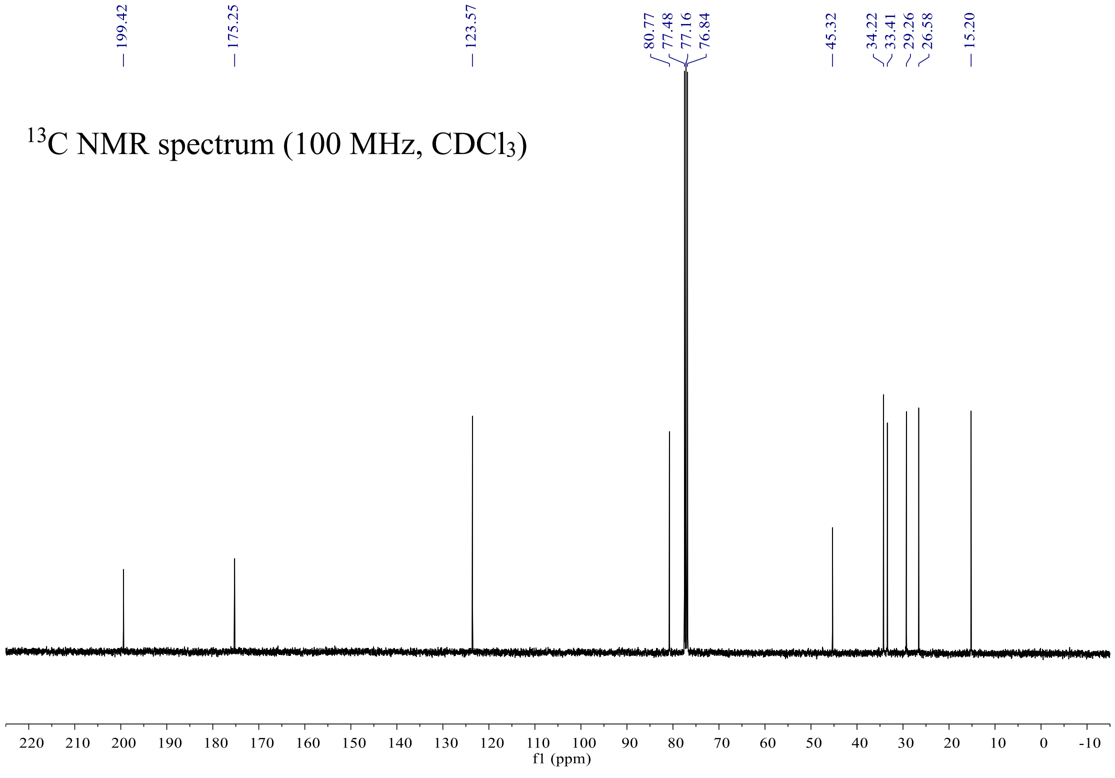


$^{13}$C NMR spectrum (100 MHz, $CDCl_3$)

**S-3**

$^{1}$H NMR spectrum (400 MHz, $CDCl_3$)
(2:1 mixture of diastereomers)

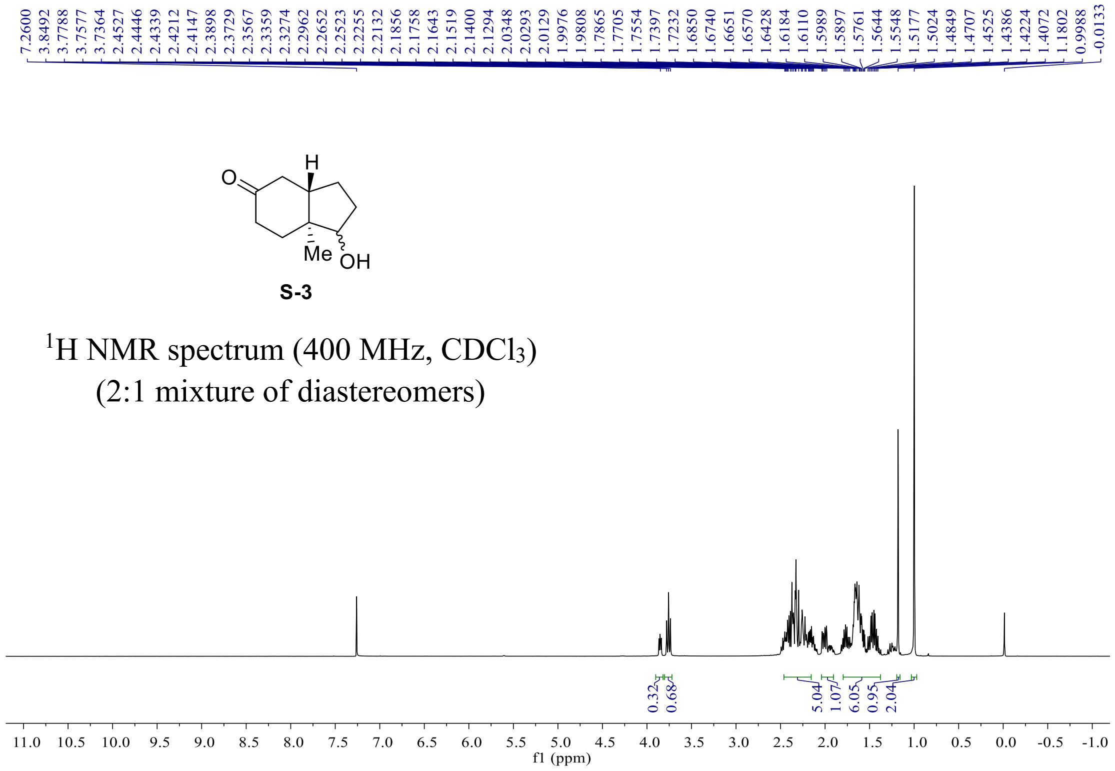


$^{13}$C NMR spectrum (100 MHz, $CDCl_3$)

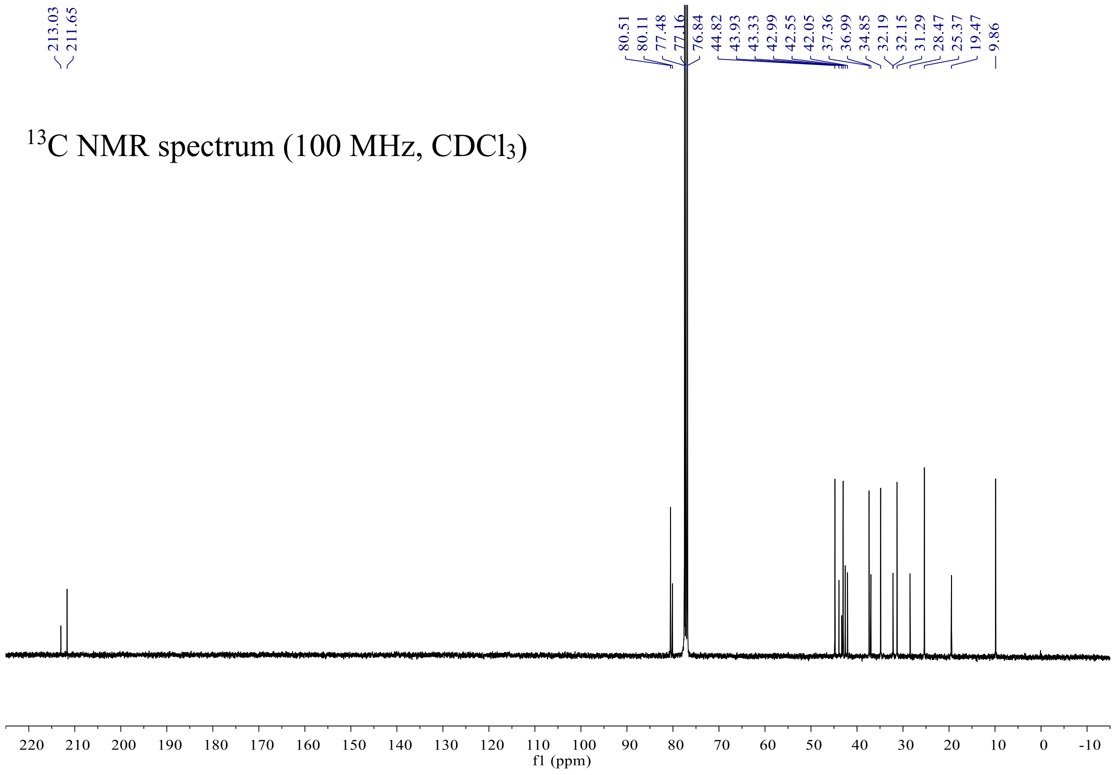

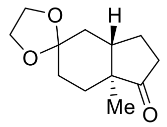


**14**

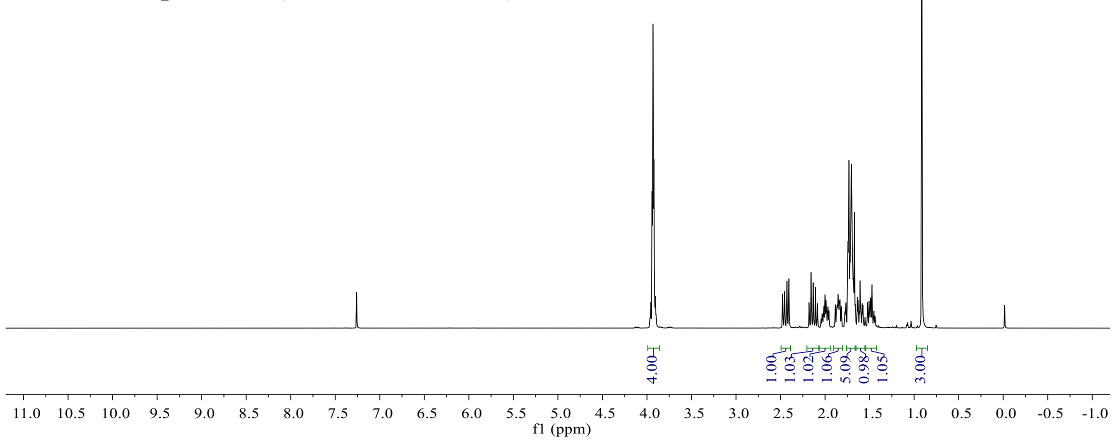


$^{1}$H NMR spectrum (400 MHz, $CDCl_3$)

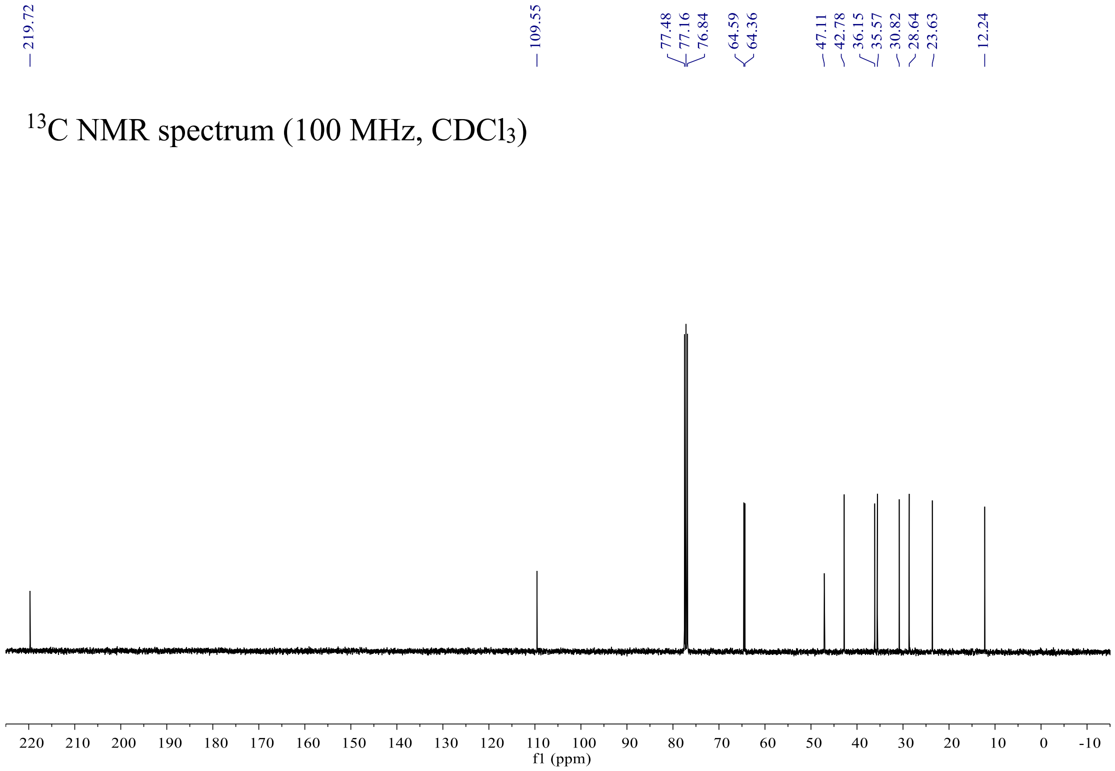


$^{13}$C NMR spectrum (100 MHz, $CDCl_3$)

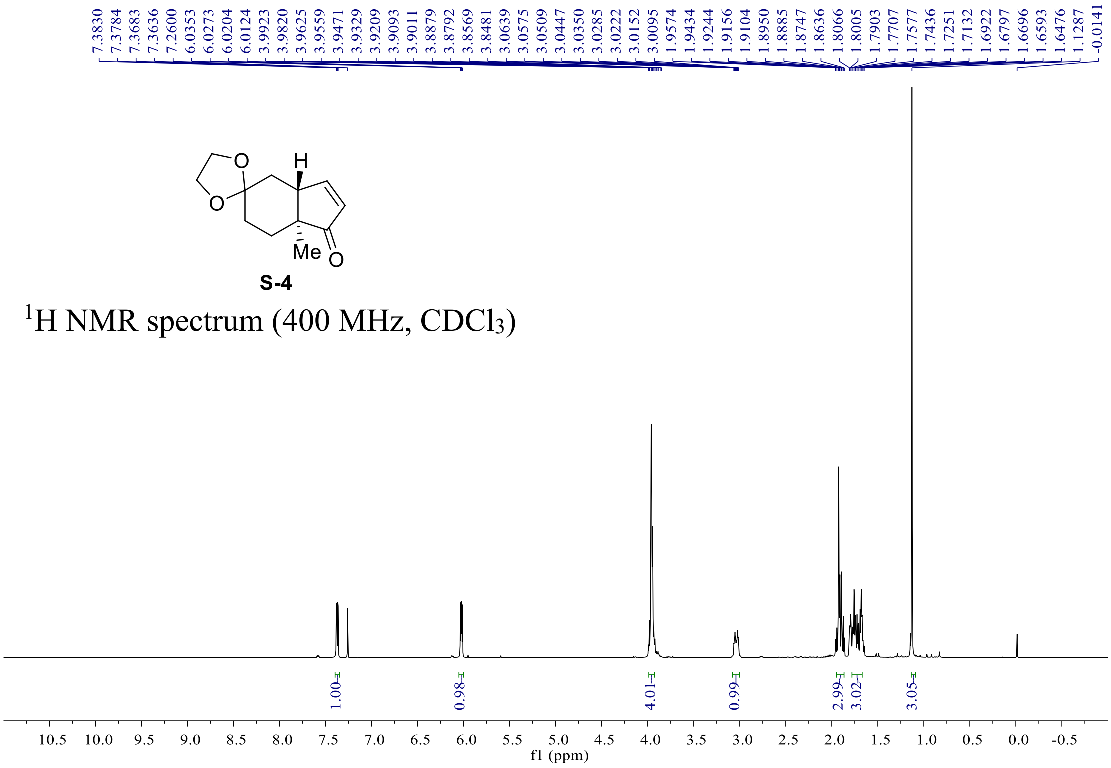


$^{1}$H NMR spectrum (400 MHz, $CDCl_3$)

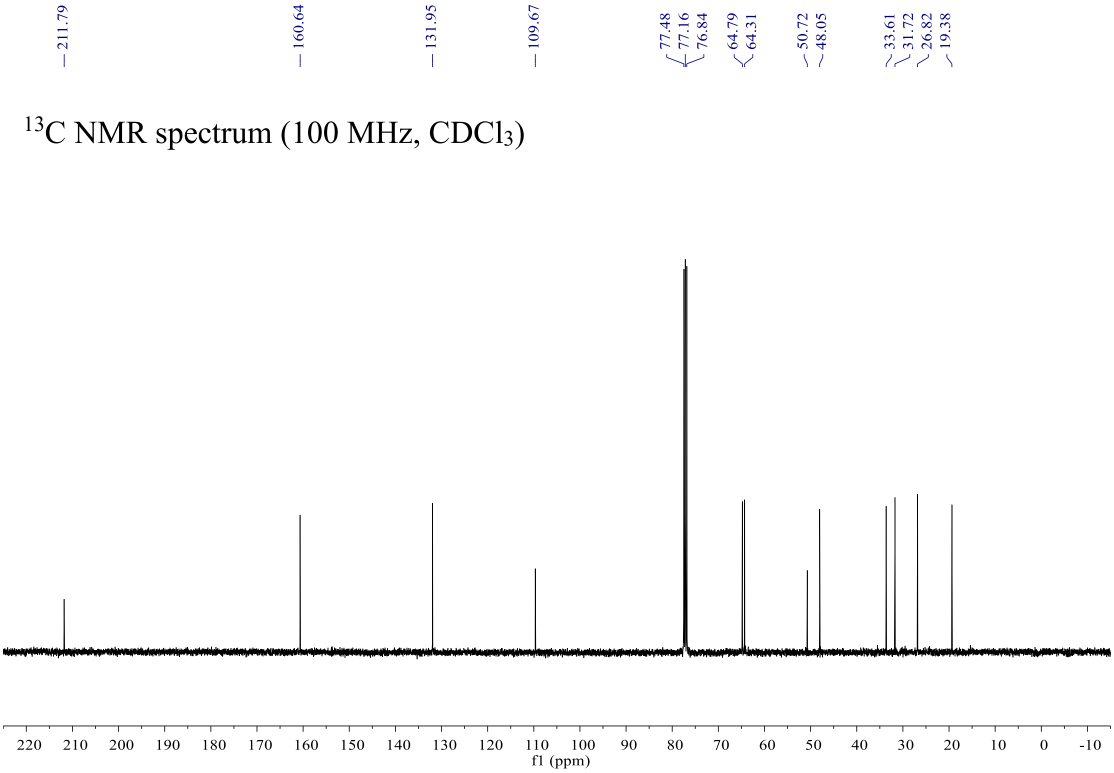


$^{13}$C NMR spectrum (100 MHz, $CDCl_3$)

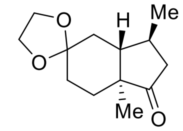


**22**

$^{1}$H NMR spectrum (400 MHz, $CDCl_3$)

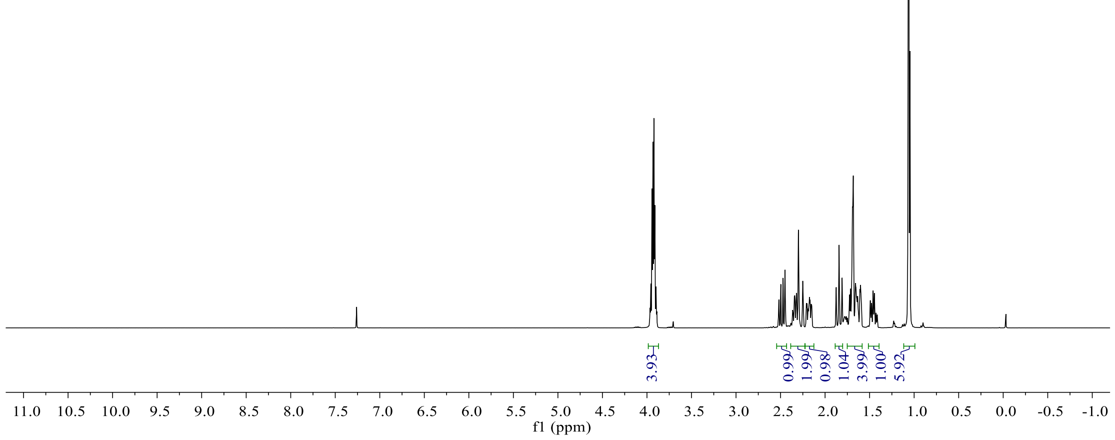


$^{13}$C NMR spectrum (100 MHz, $CDCl_3$)

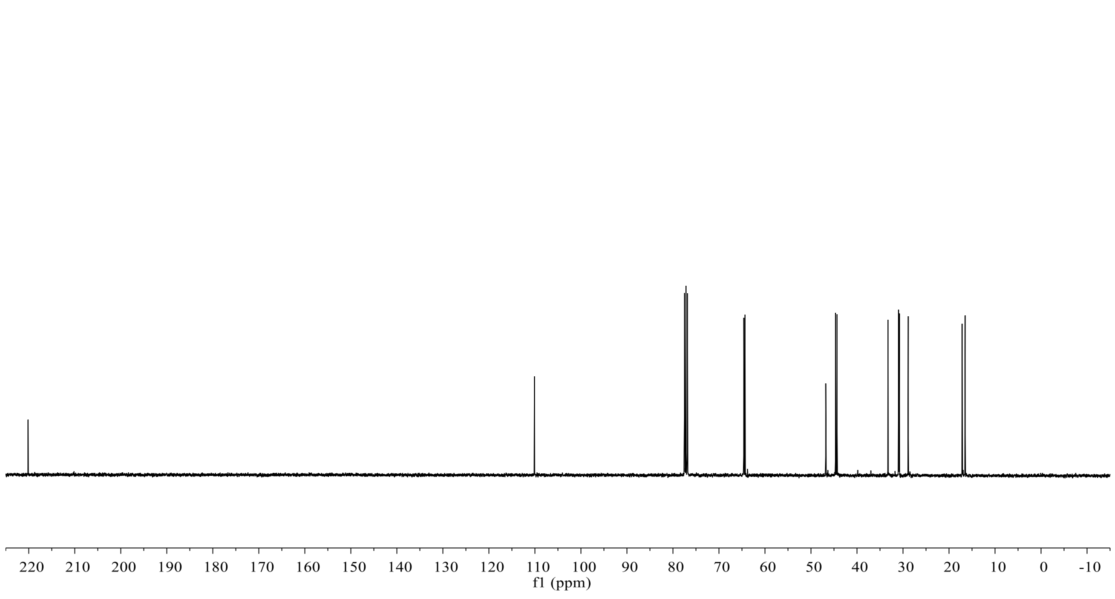

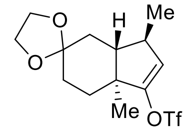


**S-5**

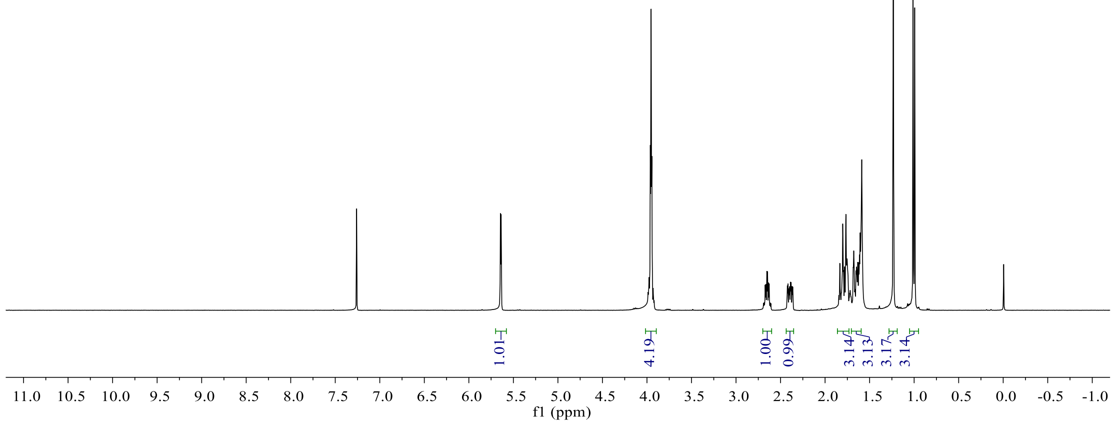


$^{1}$H NMR spectrum (400 MHz, $CDCl_3$)

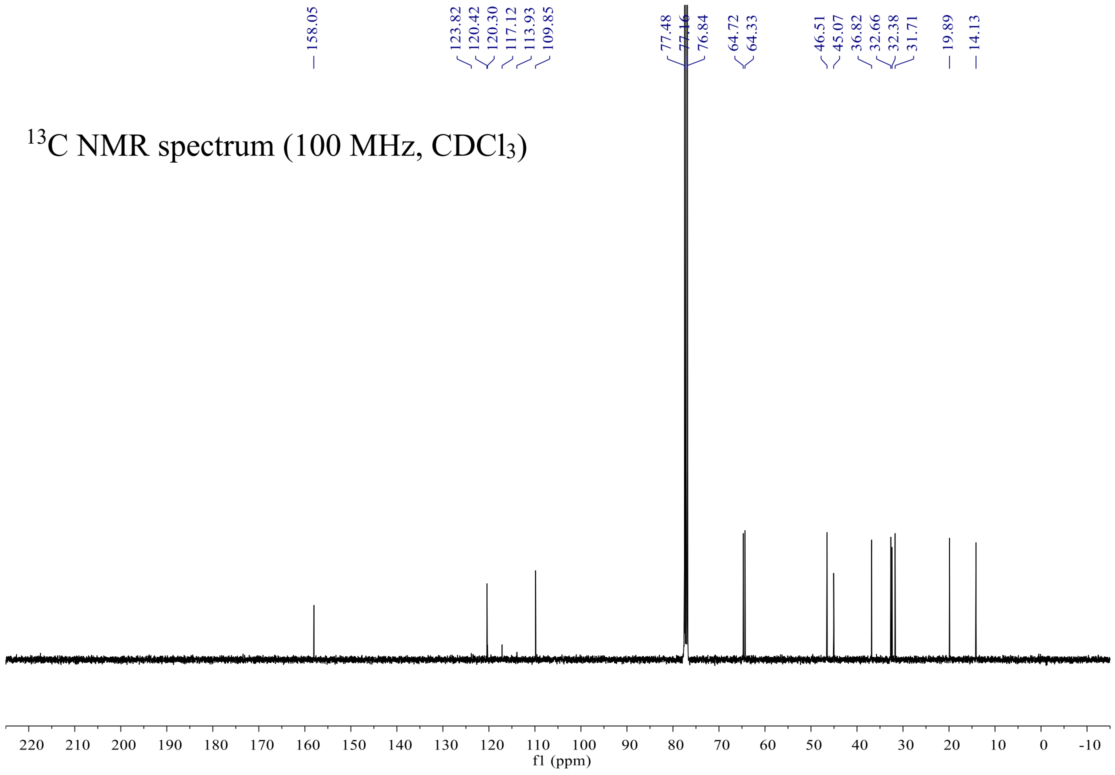


$^{13}$C NMR spectrum (100 MHz, $CDCl_3$)

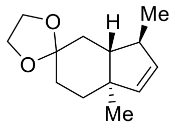


**23**

$^{1}$H NMR spectrum (400 MHz, $CDCl_3$)

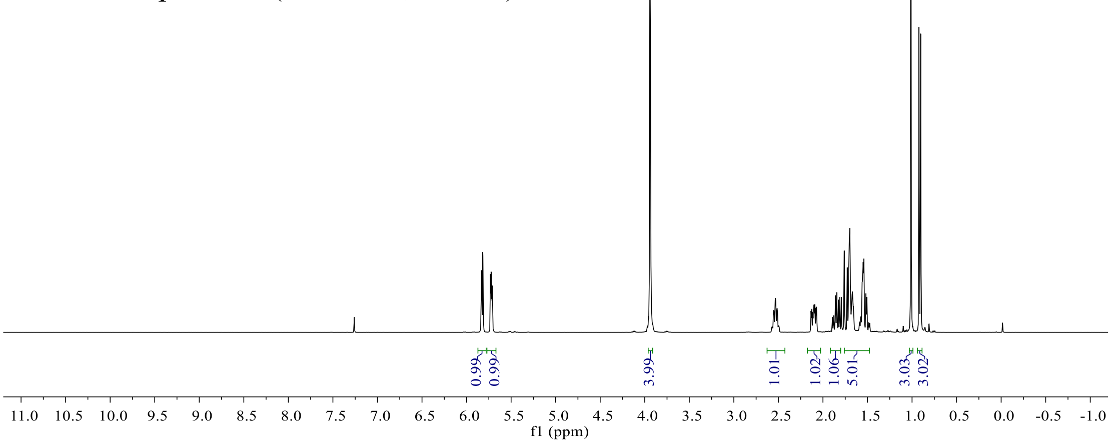


$^{13}$C NMR spectrum (100 MHz, $CDCl_3$)

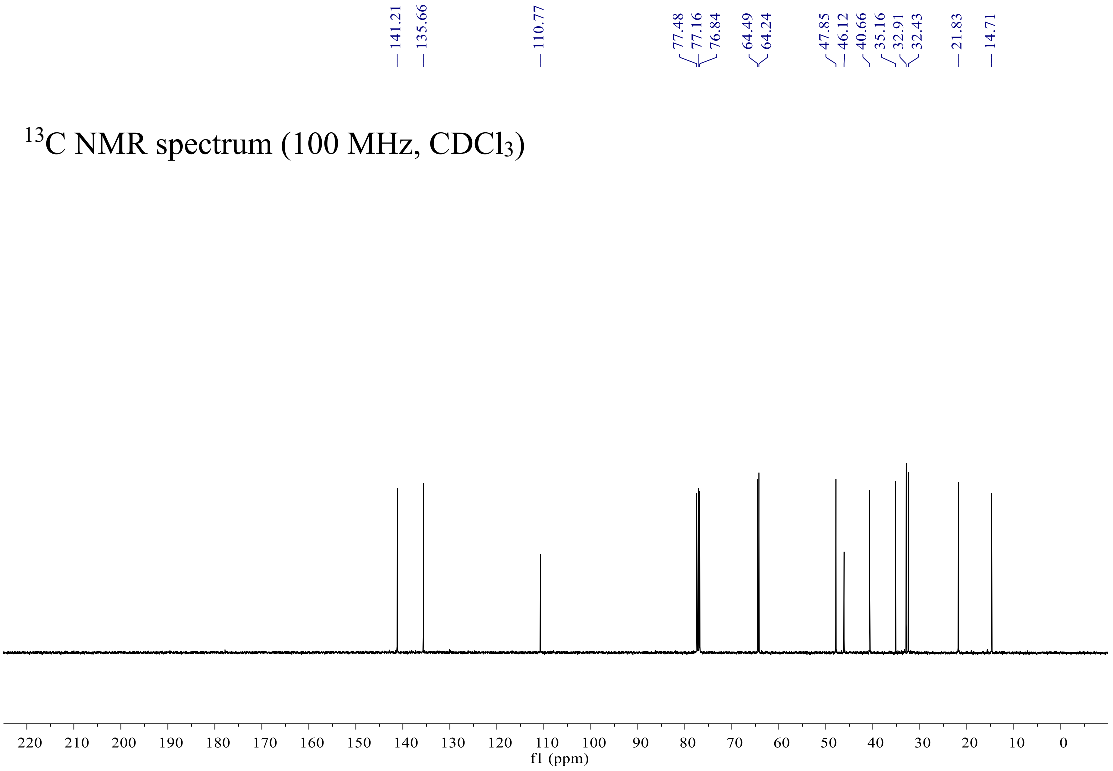

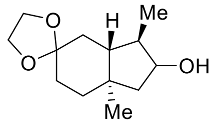


**S-6**

$^{1}$H NMR spectrum (400 MHz, $CDCl_3$)

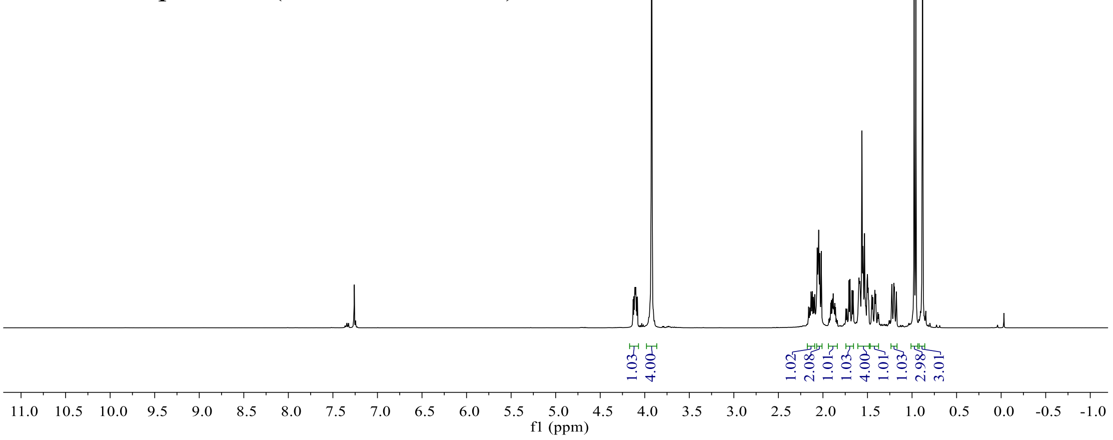


$^{13}$C NMR spectrum (100 MHz, $CDCl_3$)

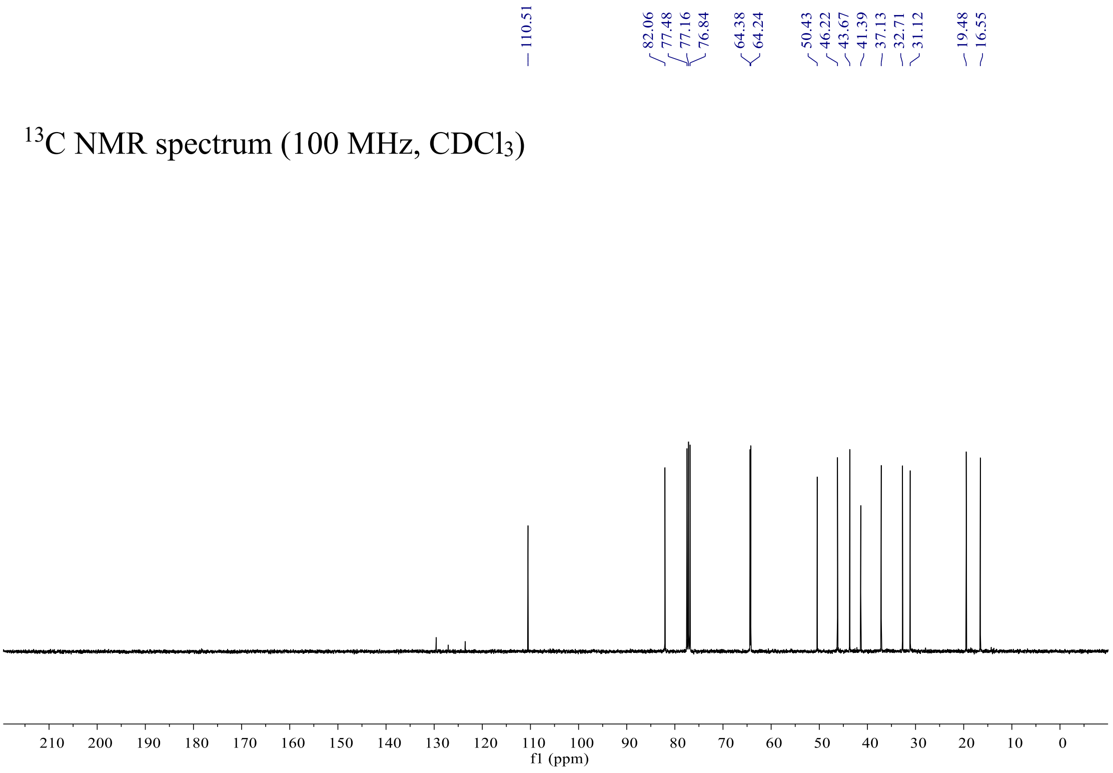

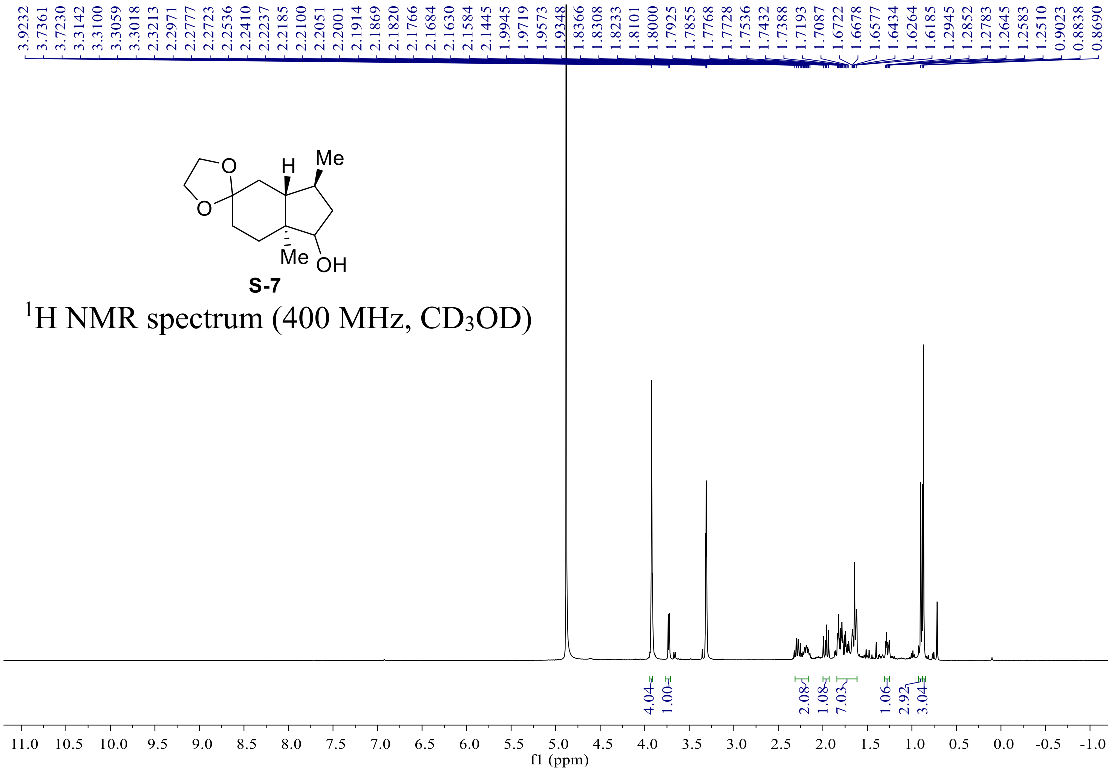


$^{1}H$ NMR spectrum (400 MHz, $CD_3OD$)

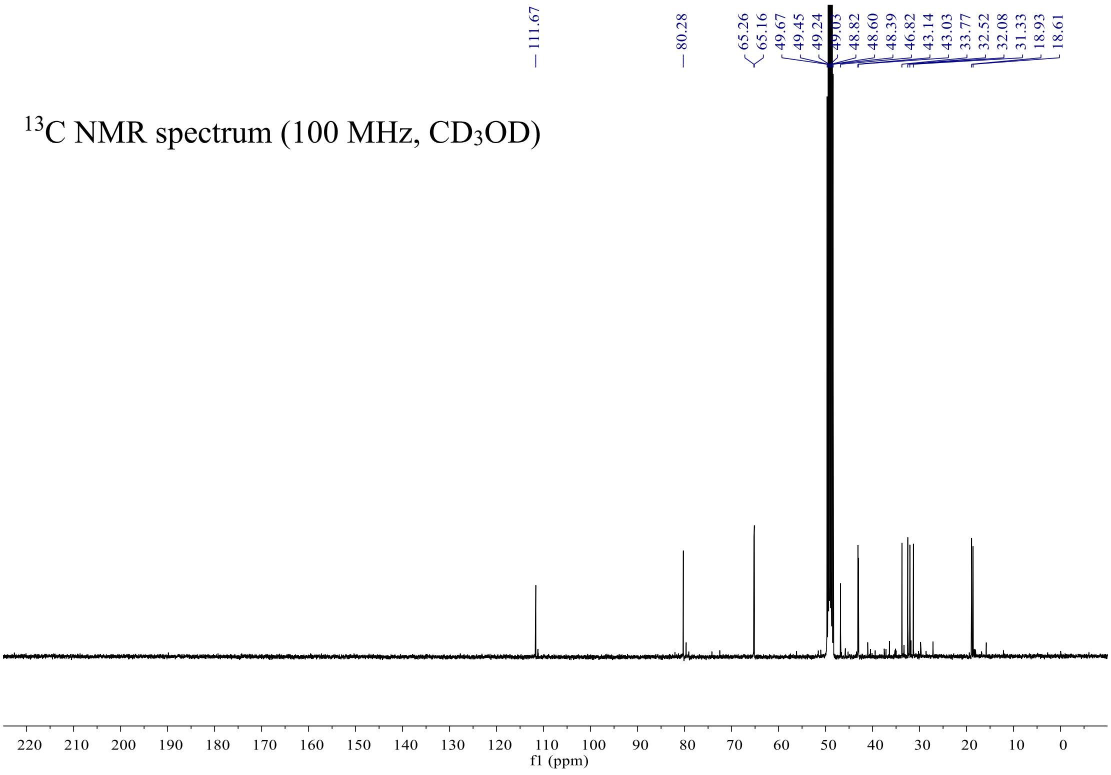


$^{13}C$ NMR spectrum (100 MHz, $CD_3OD$)

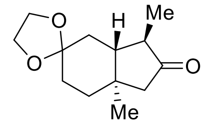

**24**

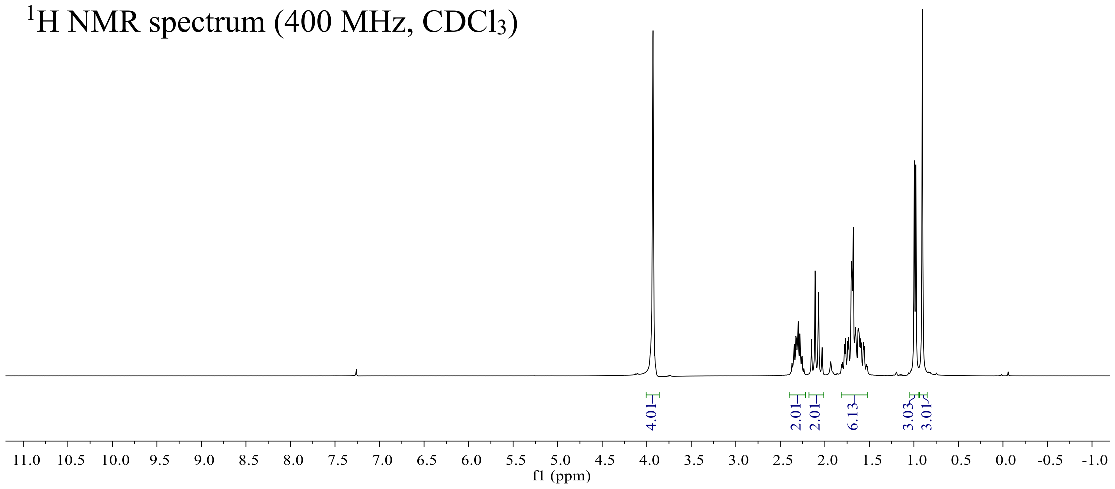


$^{1}$H NMR spectrum (400 MHz, CDCl$_3$)

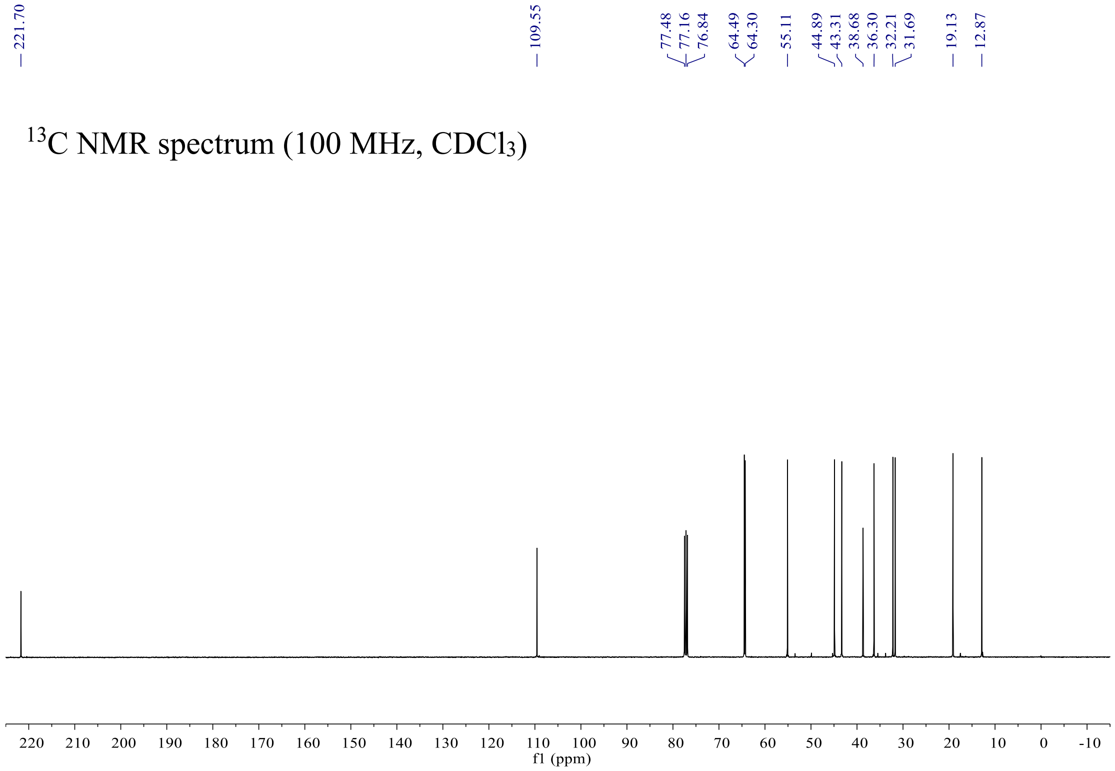


$^{13}$C NMR spectrum (100 MHz, CDCl$_3$)

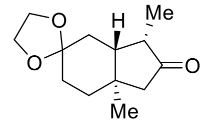


**24'**

$^{1}$H NMR spectrum (400 MHz, $CDCl_3$)

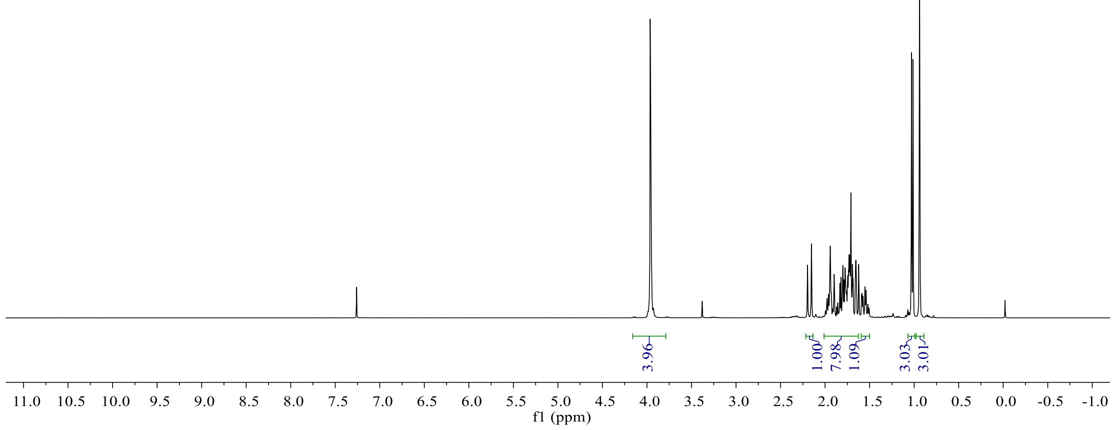


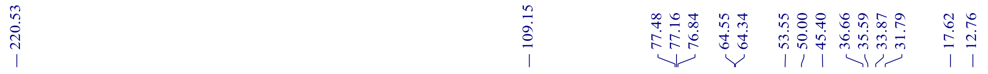


$^{13}$C NMR spectrum (100 MHz, $CDCl_3$)

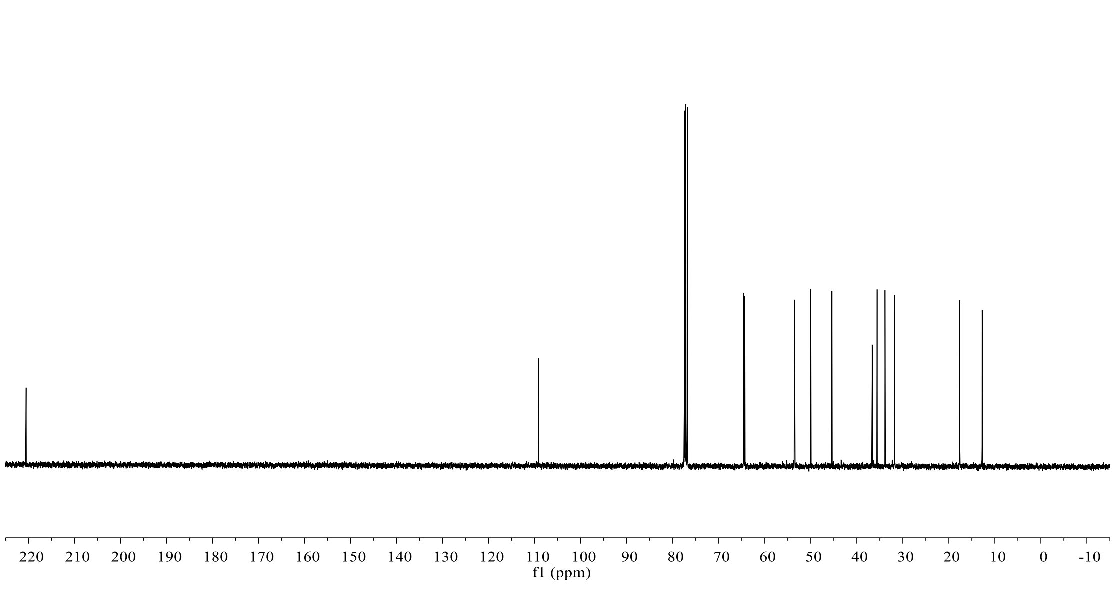

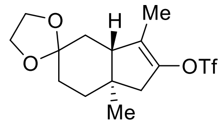

25

$^{1}$H NMR spectrum (400 MHz, $CDCl_3$)

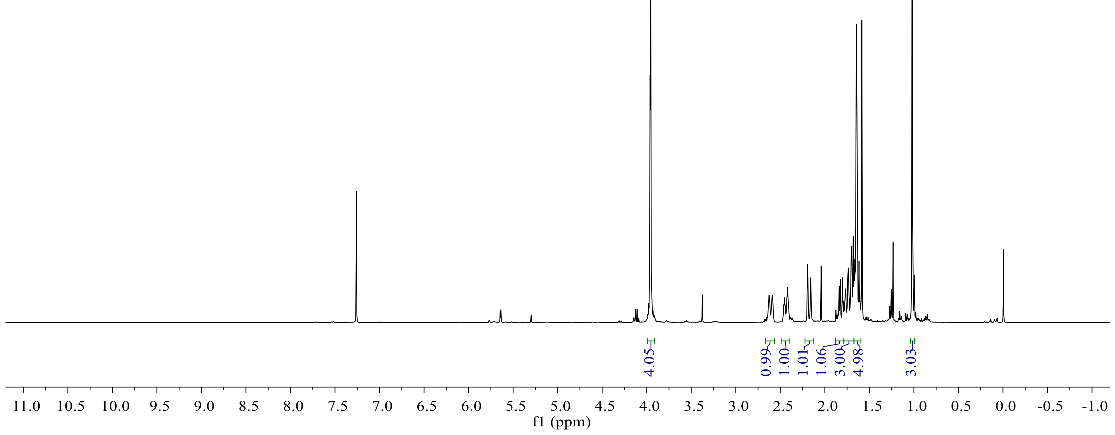

$^{13}$C NMR spectrum (100 MHz, $CDCl_3$)

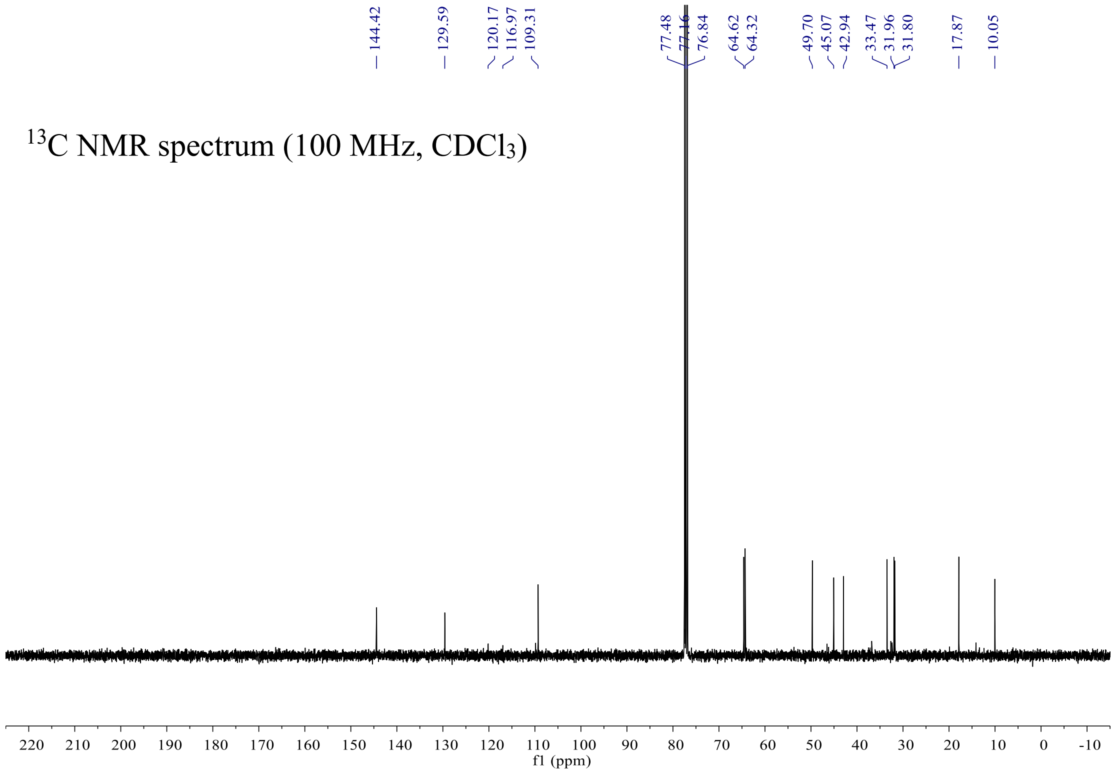

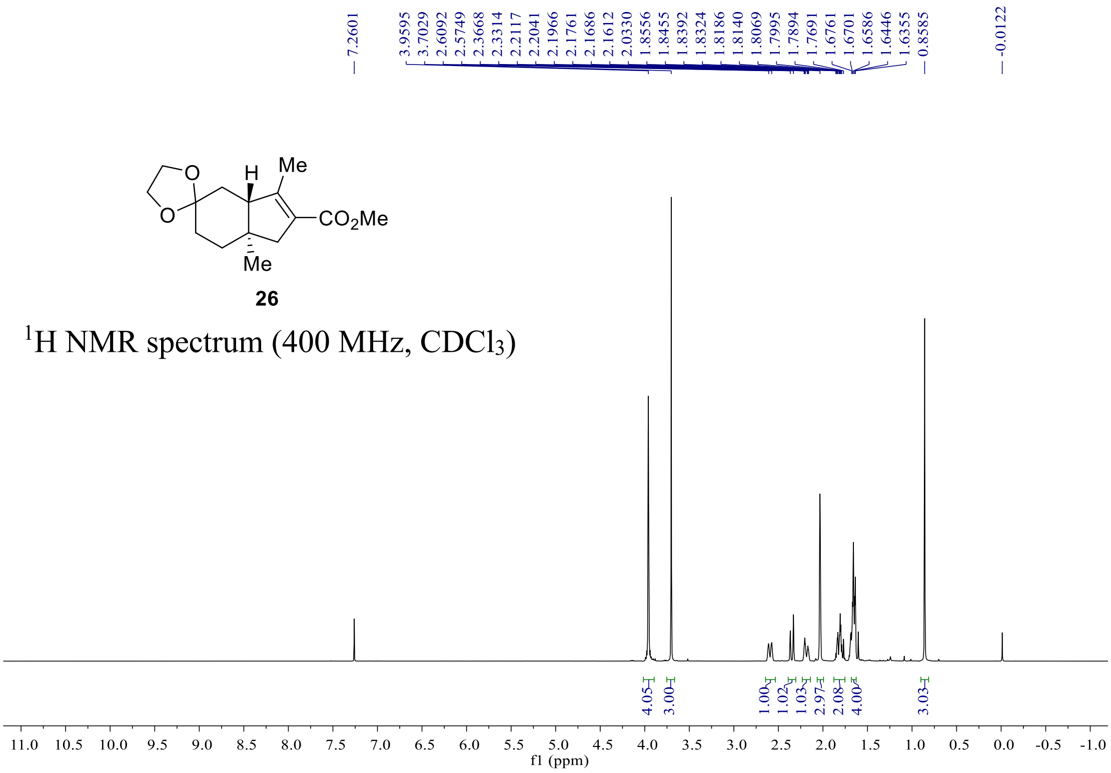


$^{13}$C NMR spectrum (100 MHz, $CDCl_3$)

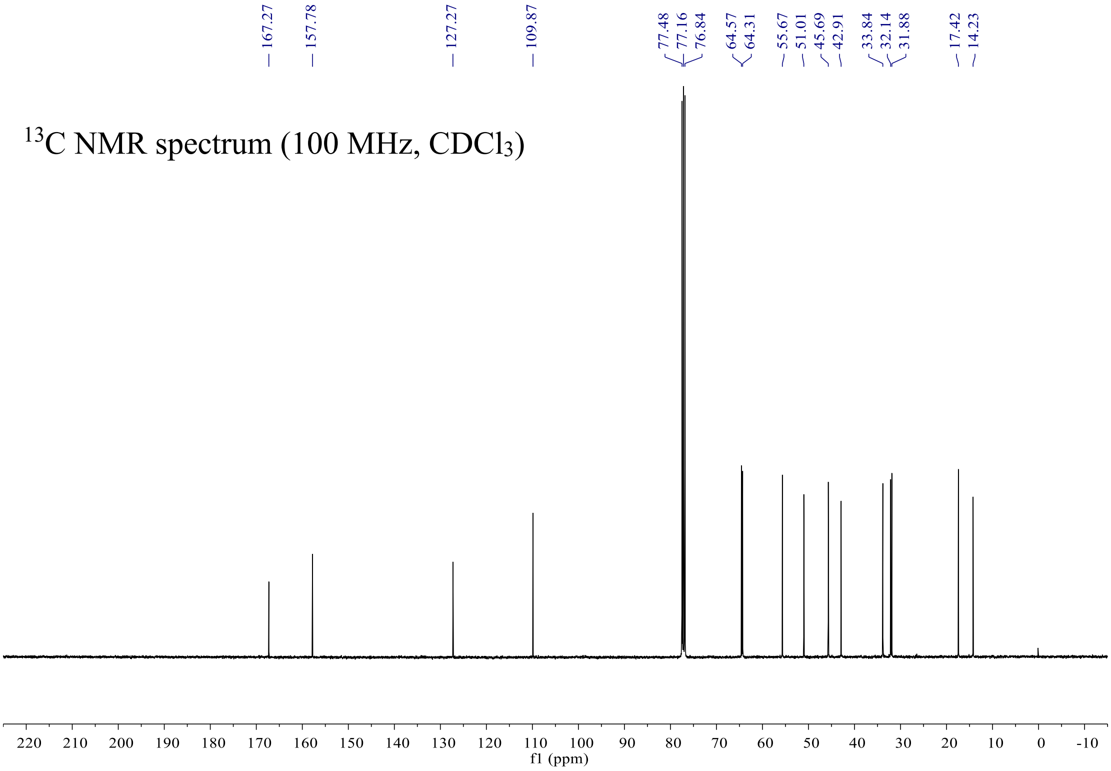

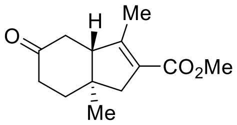


**S-8**

$^1$H NMR spectrum (400 MHz, $CDCl_3$)

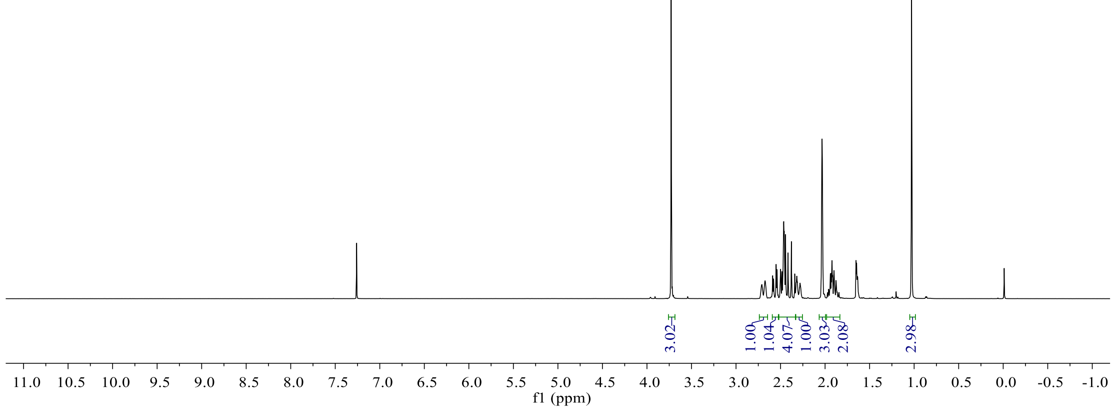


$^{13}$C NMR spectrum (100 MHz, $CDCl_3$)

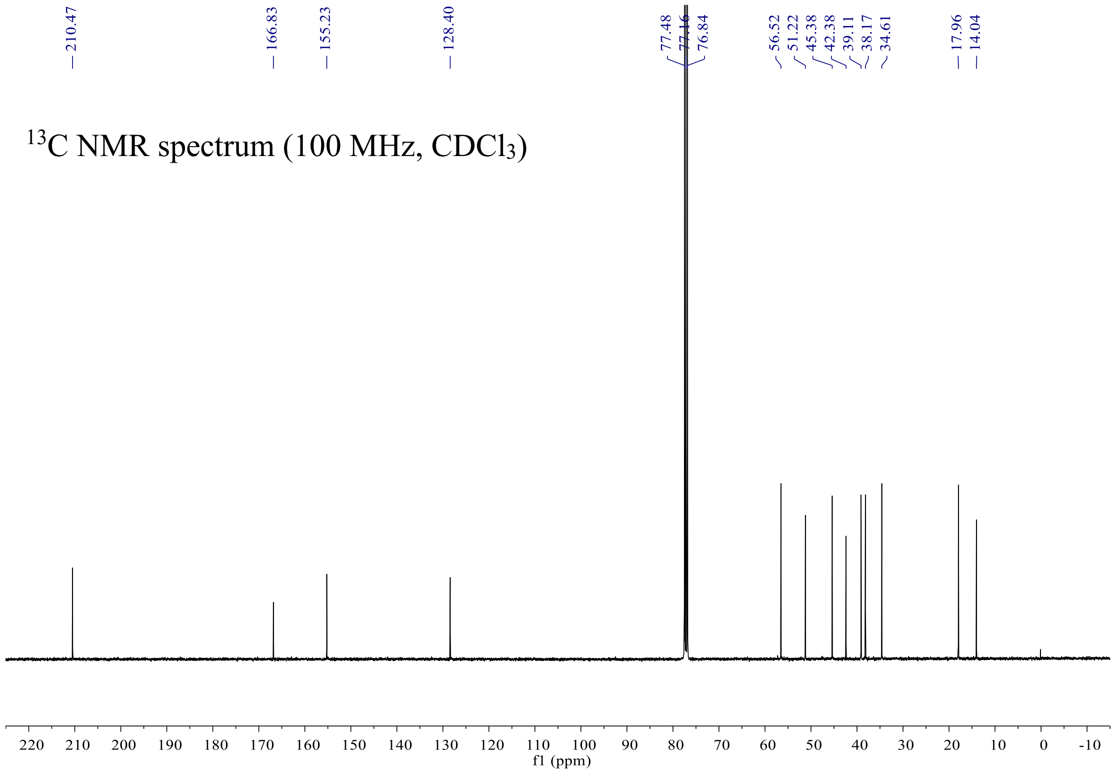

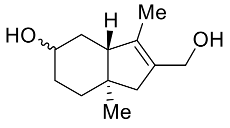

**27**

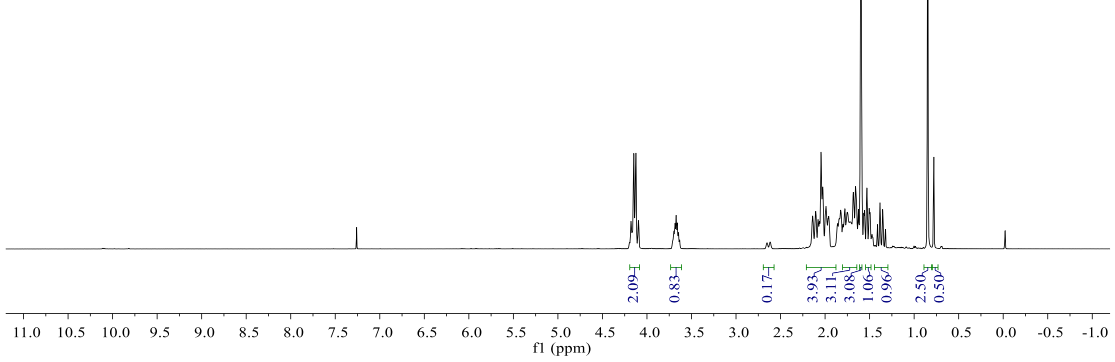


$^1$H NMR spectrum (400 MHz, $CDCl_3$)
(5:1 mixture of diastereomers)

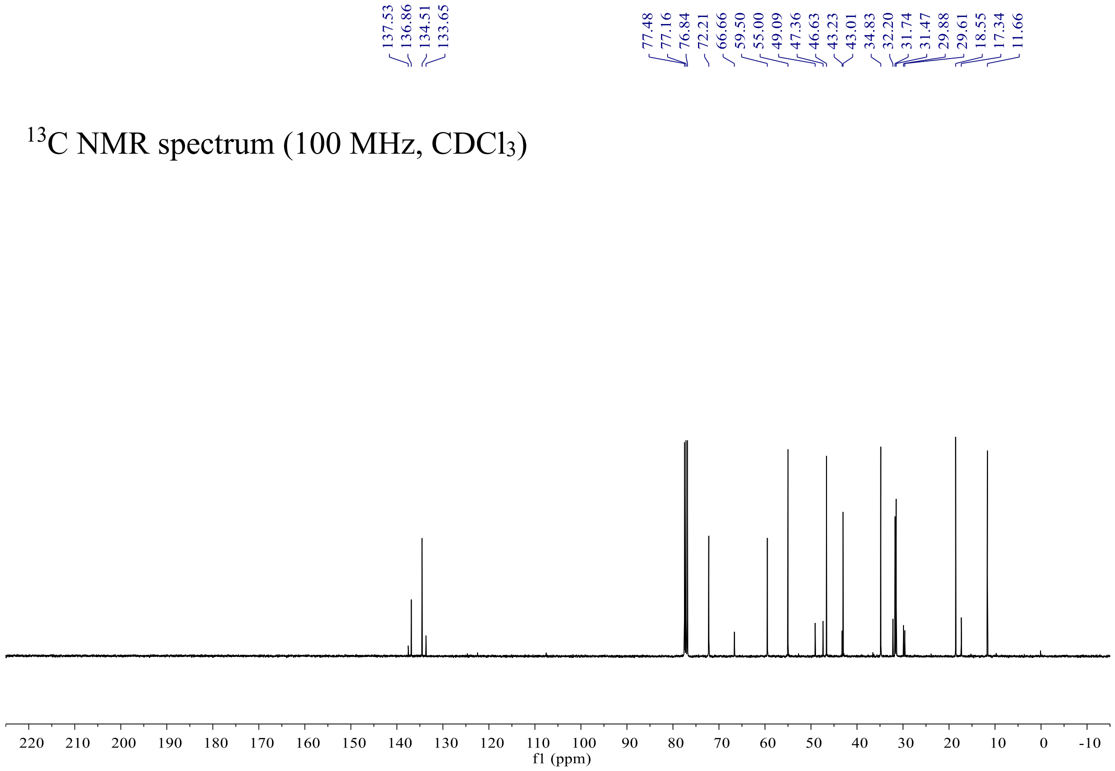


$^{13}$C NMR spectrum (100 MHz, $CDCl_3$)

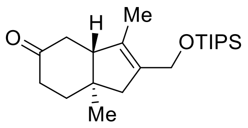

**28**

$^{1}$H NMR spectrum (400 MHz, $CDCl_3$)

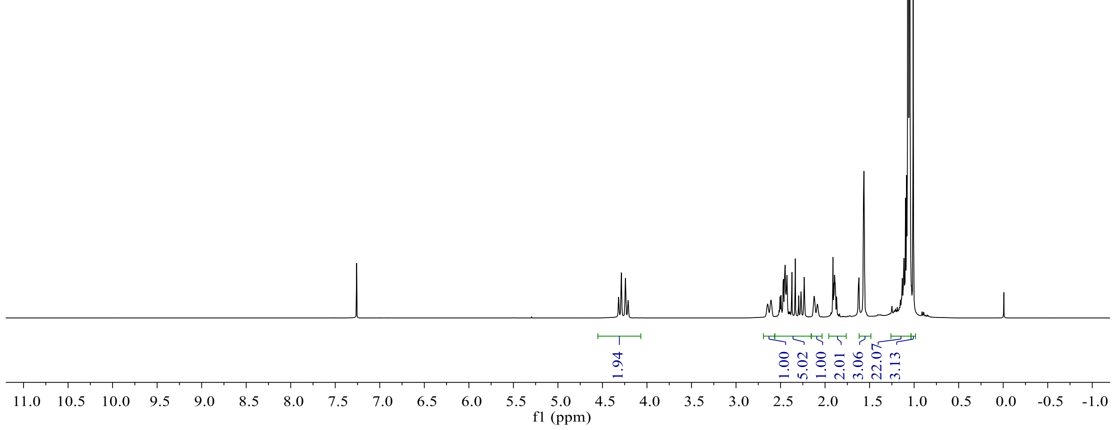


$^{13}$C NMR spectrum (100 MHz, $CDCl_3$)

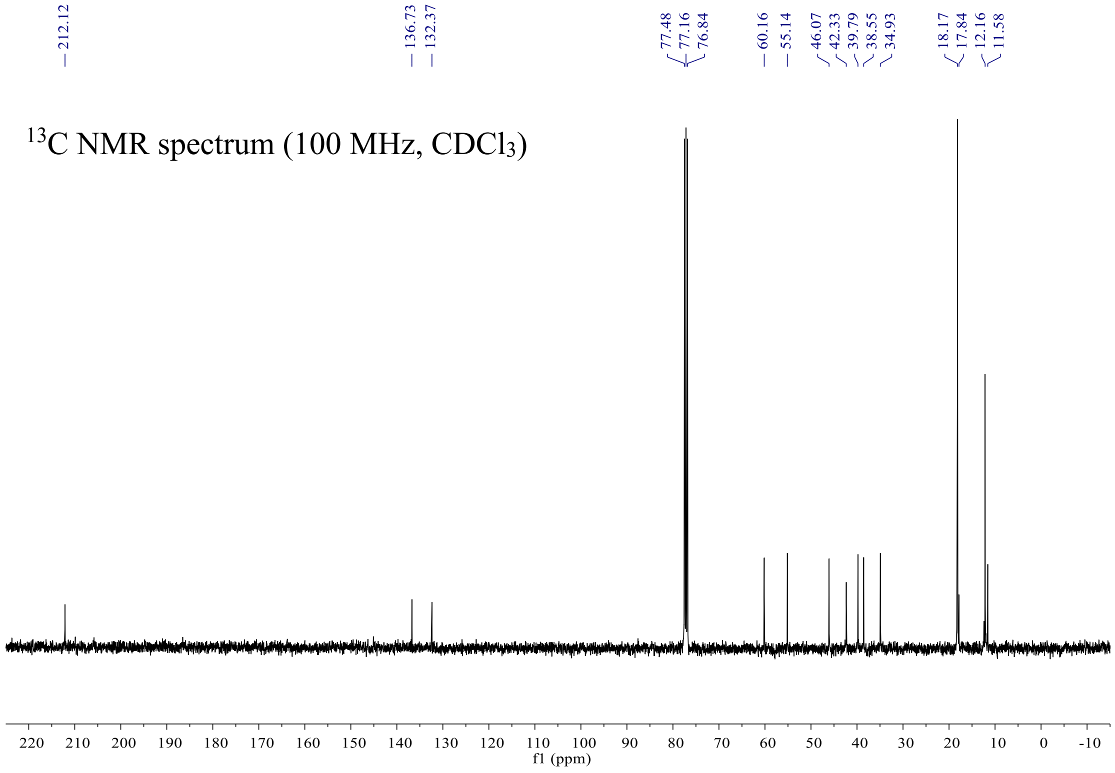

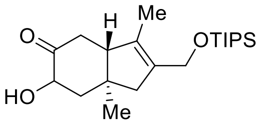


**S-9**

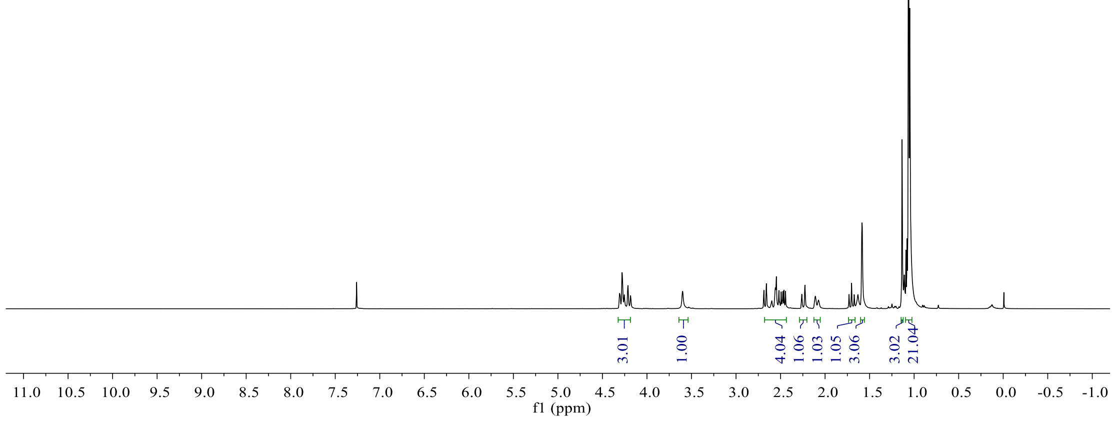


$^{1}$H NMR spectrum (400 MHz, $CDCl_3$)

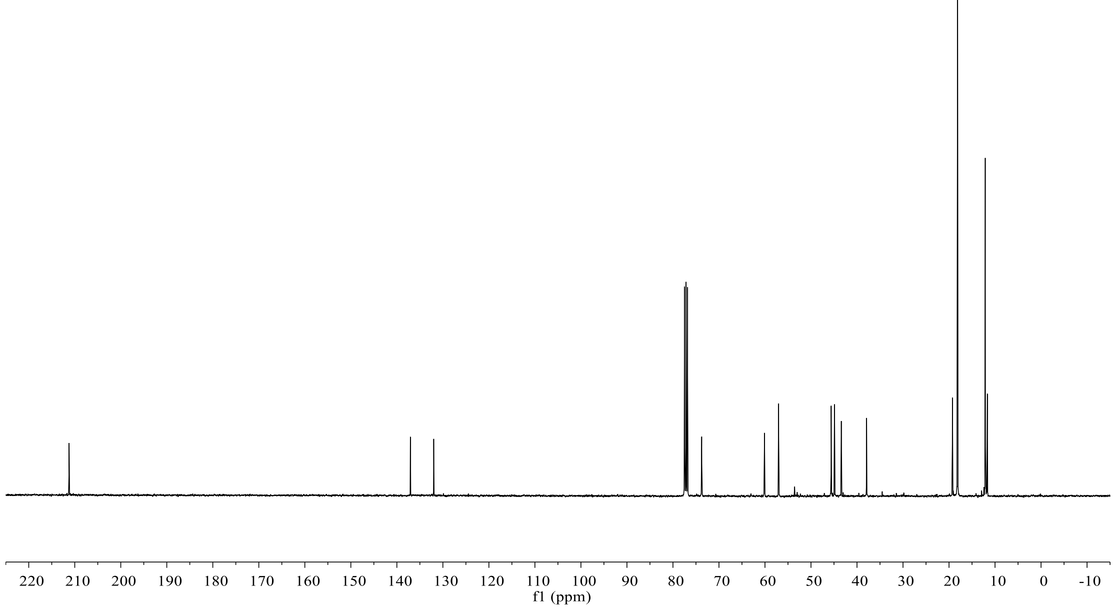


$^{13}$C NMR spectrum (100 MHz, $CDCl_3$)

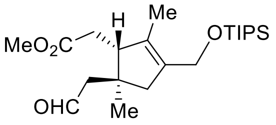


**29**

$^{1}$H NMR spectrum (400 MHz, CDCl$_3$)

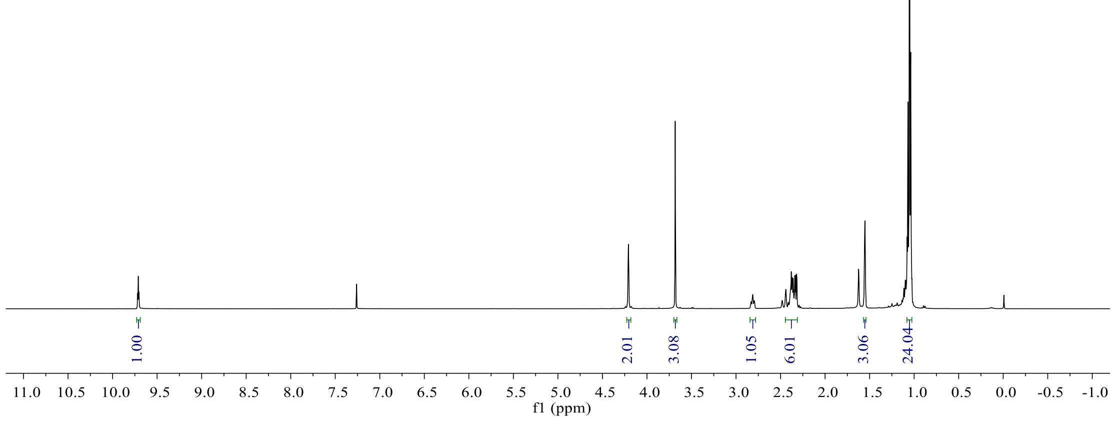


$^{13}$C NMR spectrum (100 MHz, CDCl$_3$)

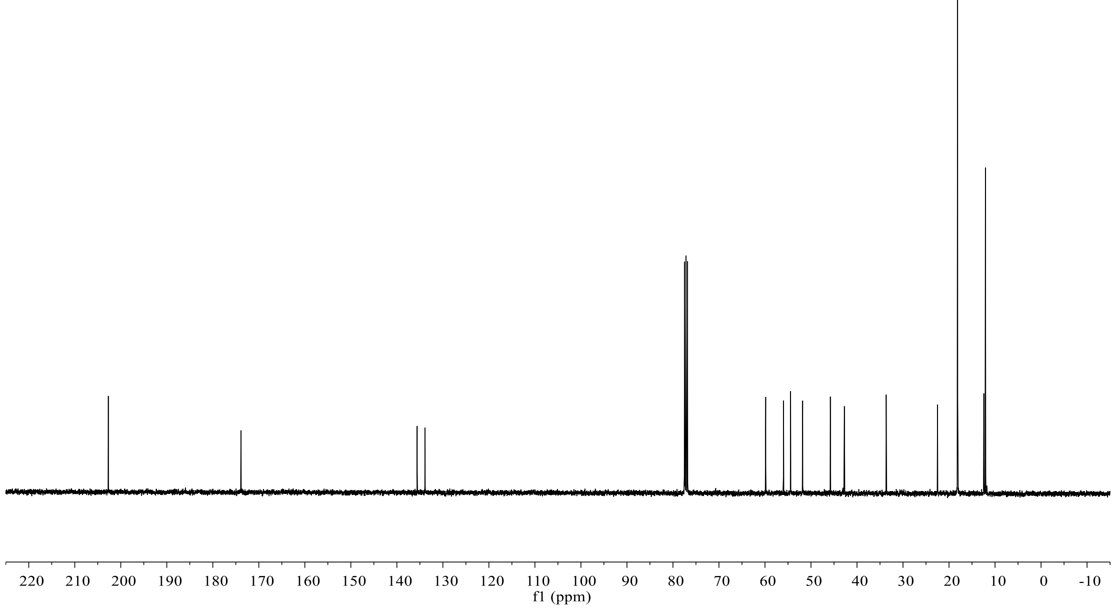

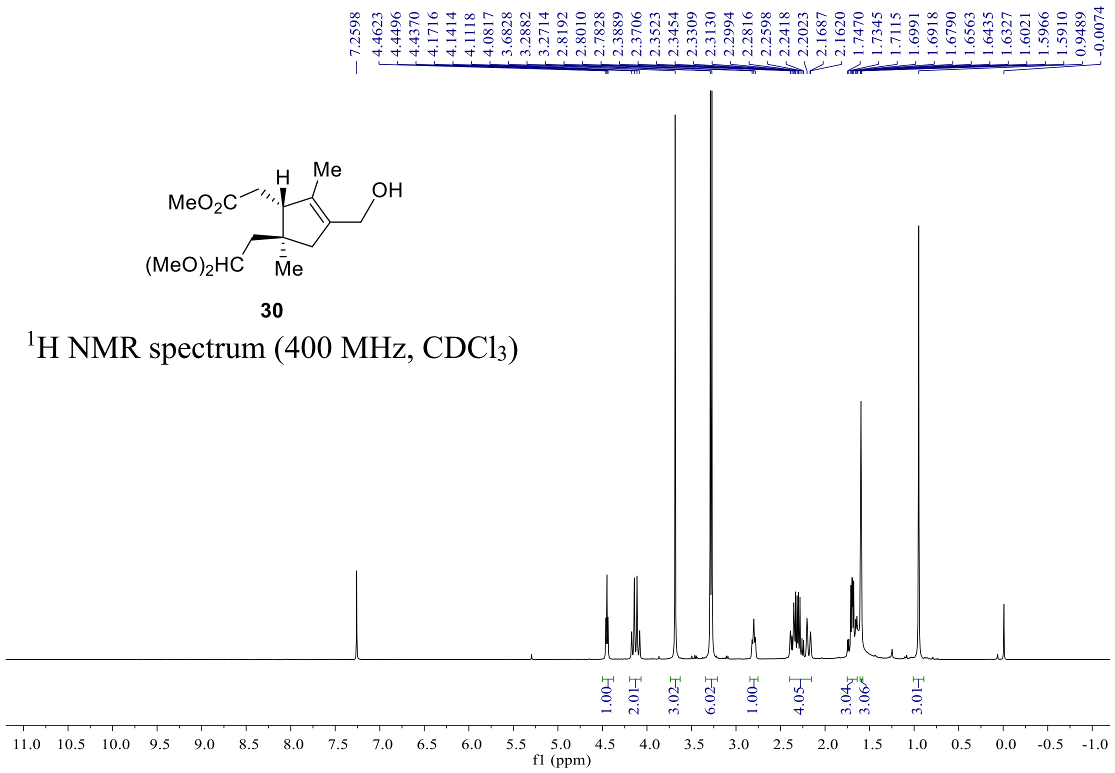
7.2598
4.4623 4.4496 4.4370 4.1716 4.1414 4.1118 4.0817 3.6828 3.2882 3.2714 2.8192 2.8010 2.7828 2.3889 2.3706 2.3523 2.3454 2.3309 2.3130 2.2994 2.2816 2.2598 2.2418 2.2023 2.1687 2.1620 1.7470 1.7345 1.7115 1.6991 1.6918 1.6790 1.6563 1.6435 1.6327 1.6021 1.5966 1.5910 0.9489 -0.0074
Me
H
OH
MeO2C
(MeO)2HC
Me
30
1H NMR spectrum (400 MHz, CDCl3)
1.00 2.01 3.02 6.02 1.00 4.05 3.04 3.06 3.01
11.0 10.5 10.0 9.5 9.0 8.5 8.0 7.5 7.0 6.5 6.0 5.5 5.0 4.5 4.0 3.5 3.0 2.5 2.0 1.5 1.0 0.5 0.0 -0.5 -1.0
f1 (ppm)

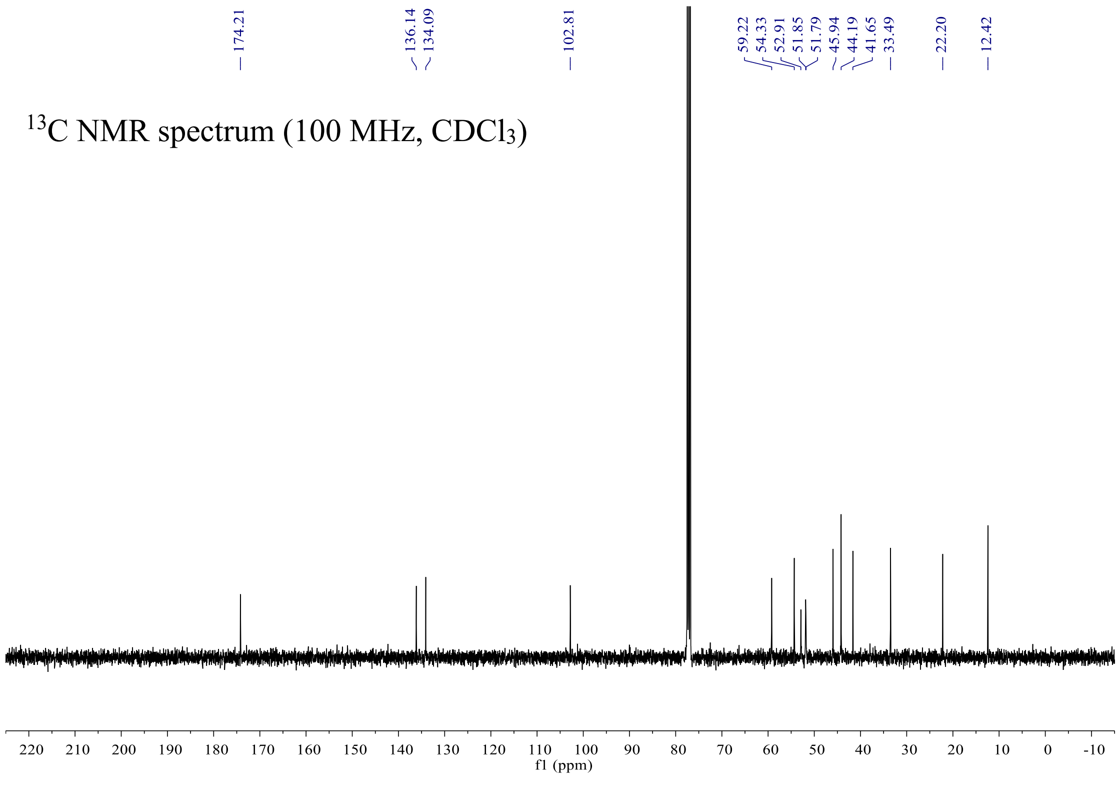
174.21
136.14 134.09
102.81
59.22 54.33 52.91 51.85 51.79 45.94 44.19 41.65 33.49
22.20
12.42
13C NMR spectrum (100 MHz, CDCl3)
220 210 200 190 180 170 160 150 140 130 120 110 100 90 80 70 60 50 40 30 20 10 0 -10
f1 (ppm)

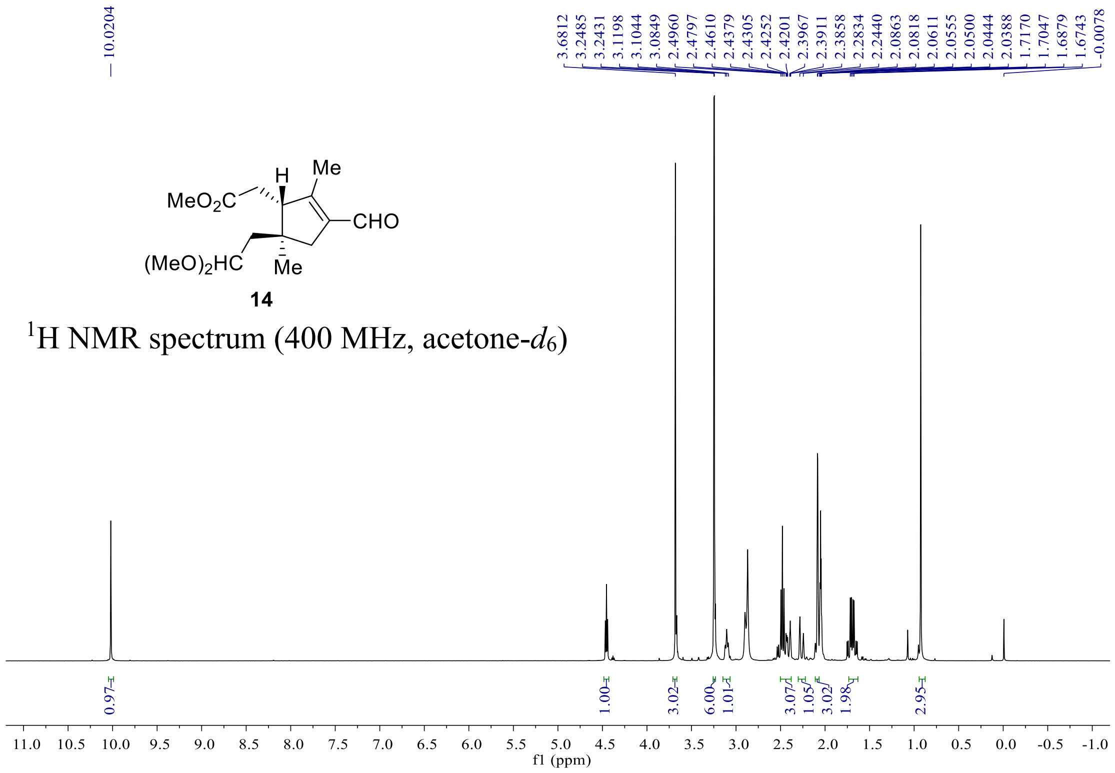
10.0204
3.6812
3.2485
3.2431
3.1198
3.1044
3.0849
2.4960
2.4797
2.4610
2.4379
2.4305
2.4252
2.4201
2.3967
2.3911
2.3858
2.2834
2.2440
2.0863
2.0818
2.0611
2.0555
2.0500
2.0444
2.0388
1.7170
1.7047
1.6879
1.6743
-0.0078
MeO2C
H
Me
CHO
(MeO)2HC
Me
14
1H NMR spectrum (400 MHz, acetone-d6)
0.97
1.00
3.02
6.00
1.01
3.07
1.05
3.02
1.98
2.95
f1 (ppm)

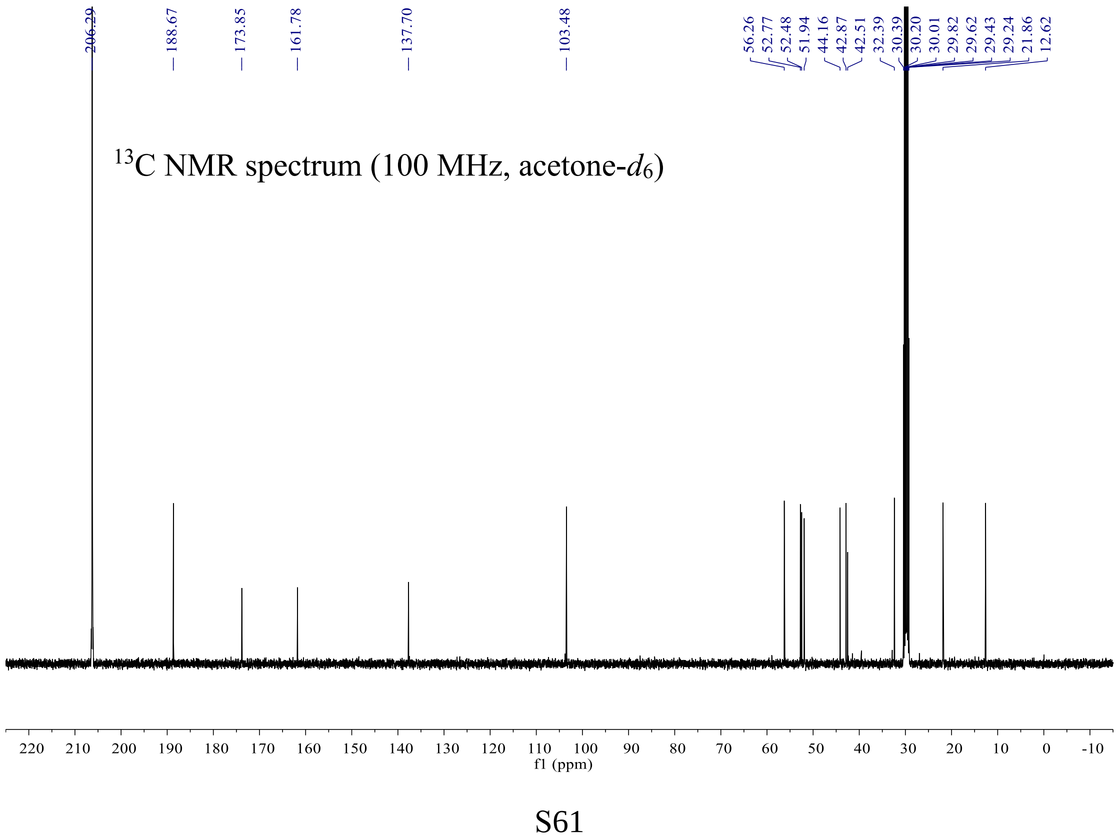
206.29
188.67
173.85
161.78
137.70
103.48
56.26
52.77
52.48
51.94
44.16
42.87
42.51
32.39
30.39
30.20
30.01
29.82
29.62
29.43
29.24
21.86
12.62
13C NMR spectrum (100 MHz, acetone-d6)
f1 (ppm)

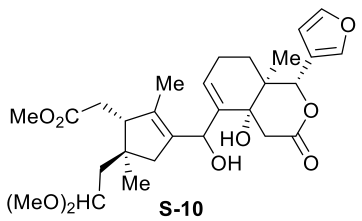

S-10

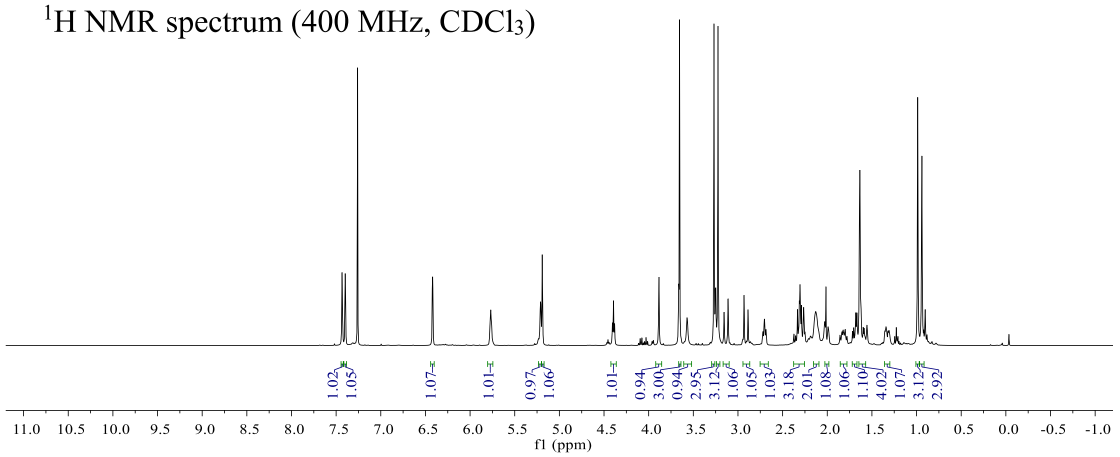

$^{1}$H NMR spectrum (400 MHz, $CDCl_3$)

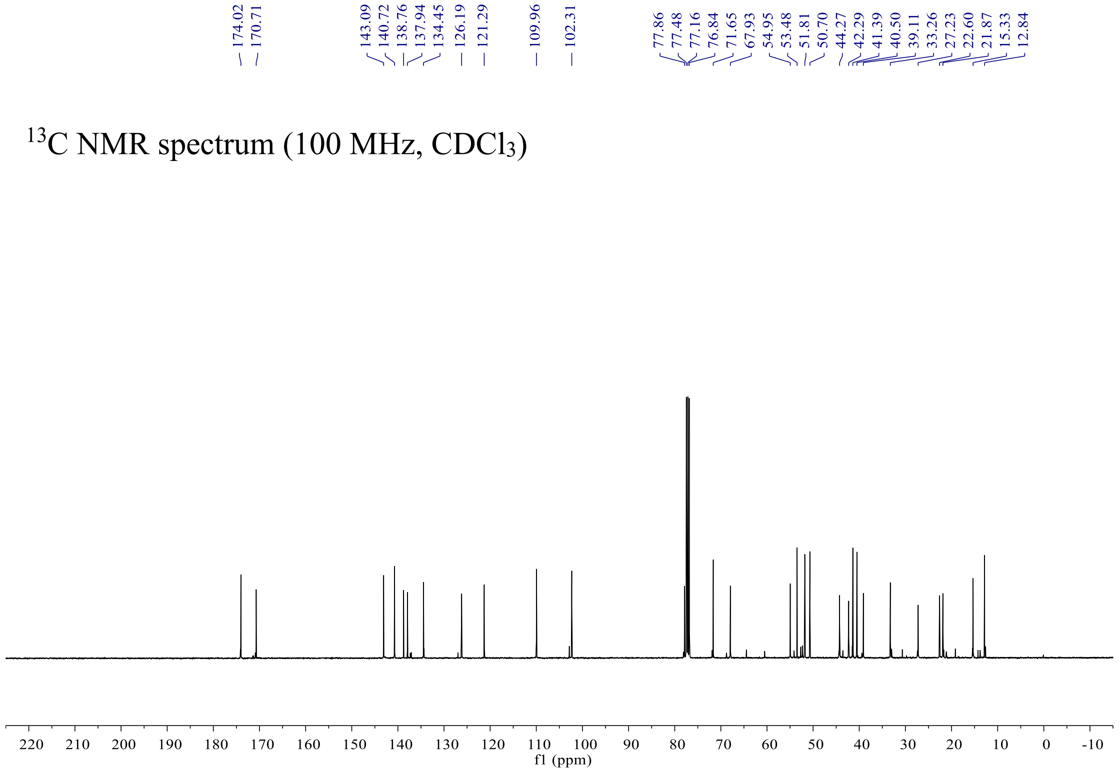

$^{13}$C NMR spectrum (100 MHz, $CDCl_3$)

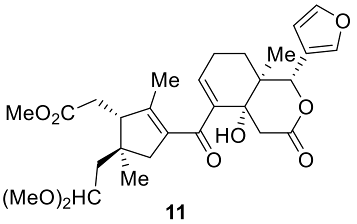


**11**

$^{1}$H NMR spectrum (400 MHz, $CDCl_3$)

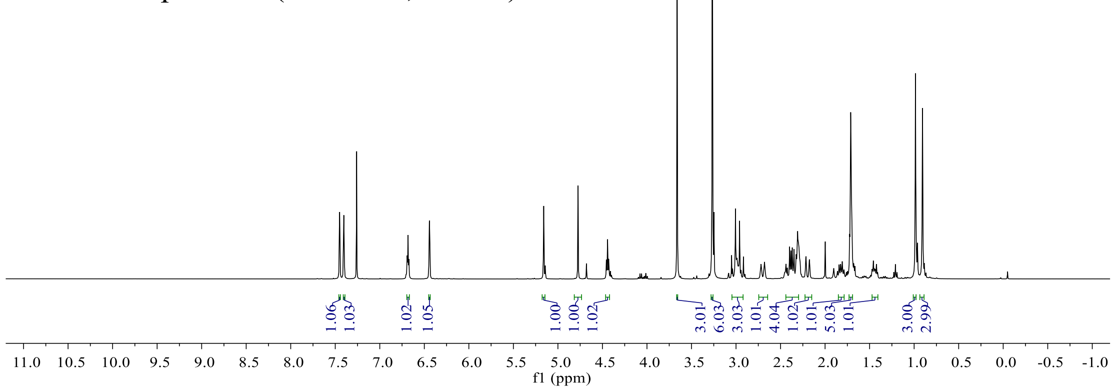


$^{13}$C NMR spectrum (100 MHz, $CDCl_3$)

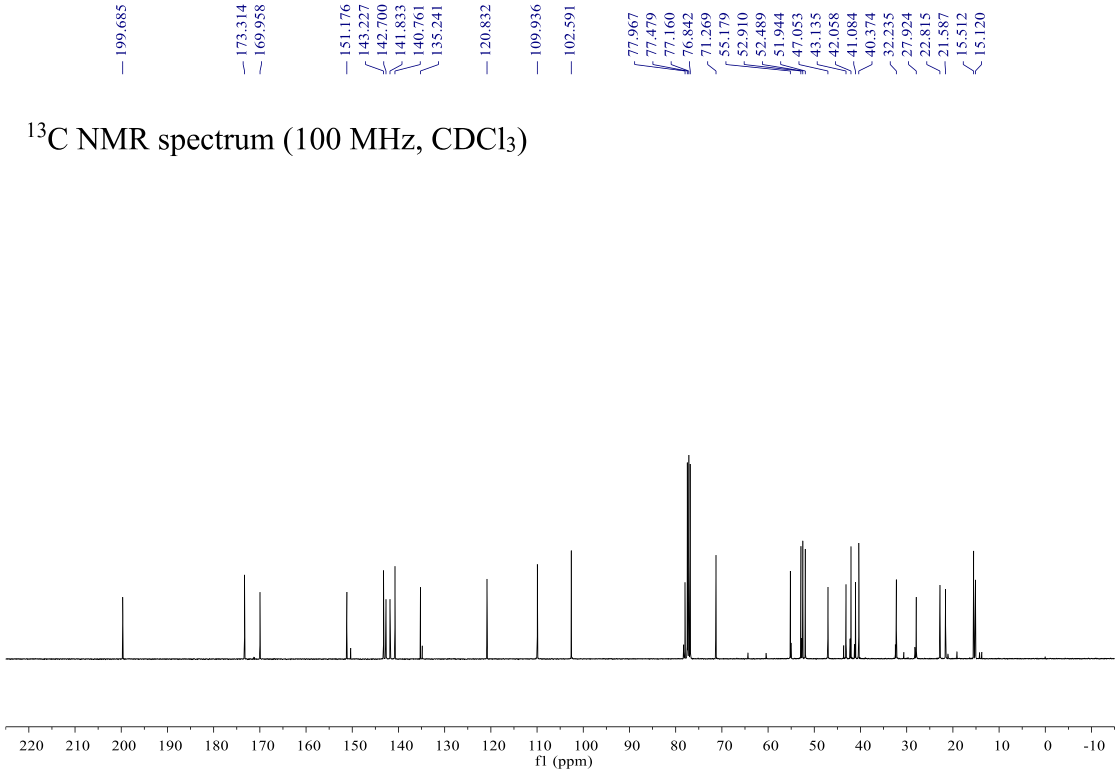

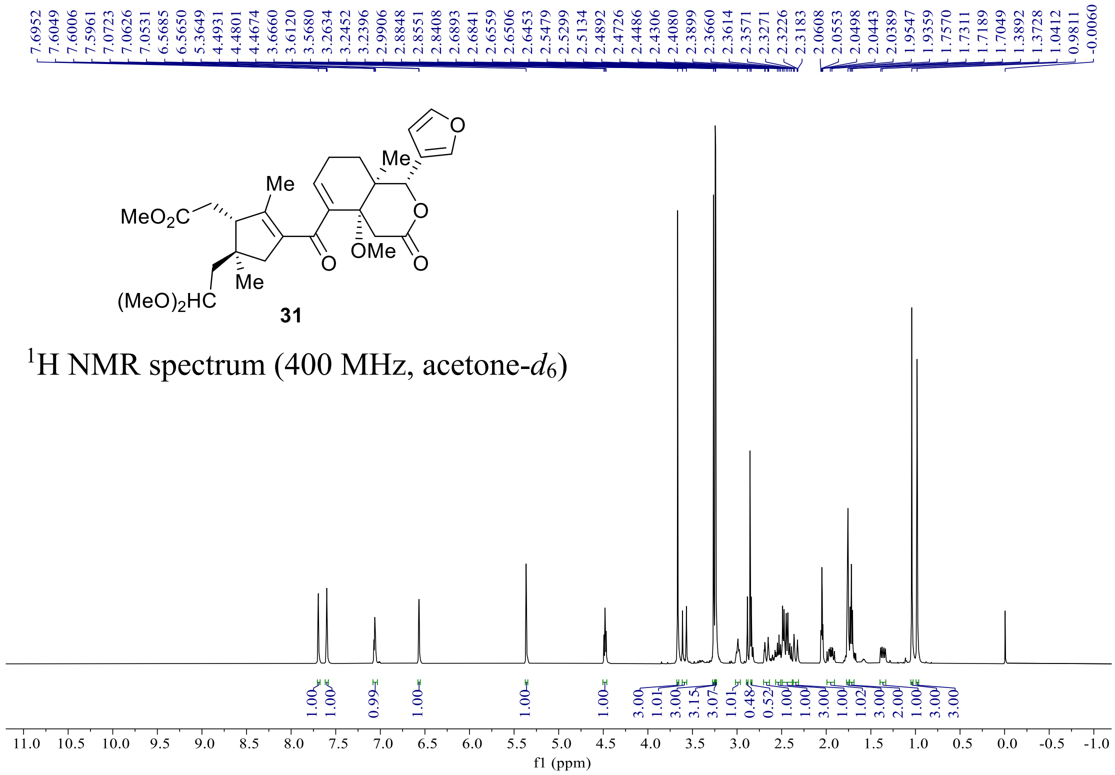


$^{1}$H NMR spectrum (400 MHz, acetone-$d_6$)

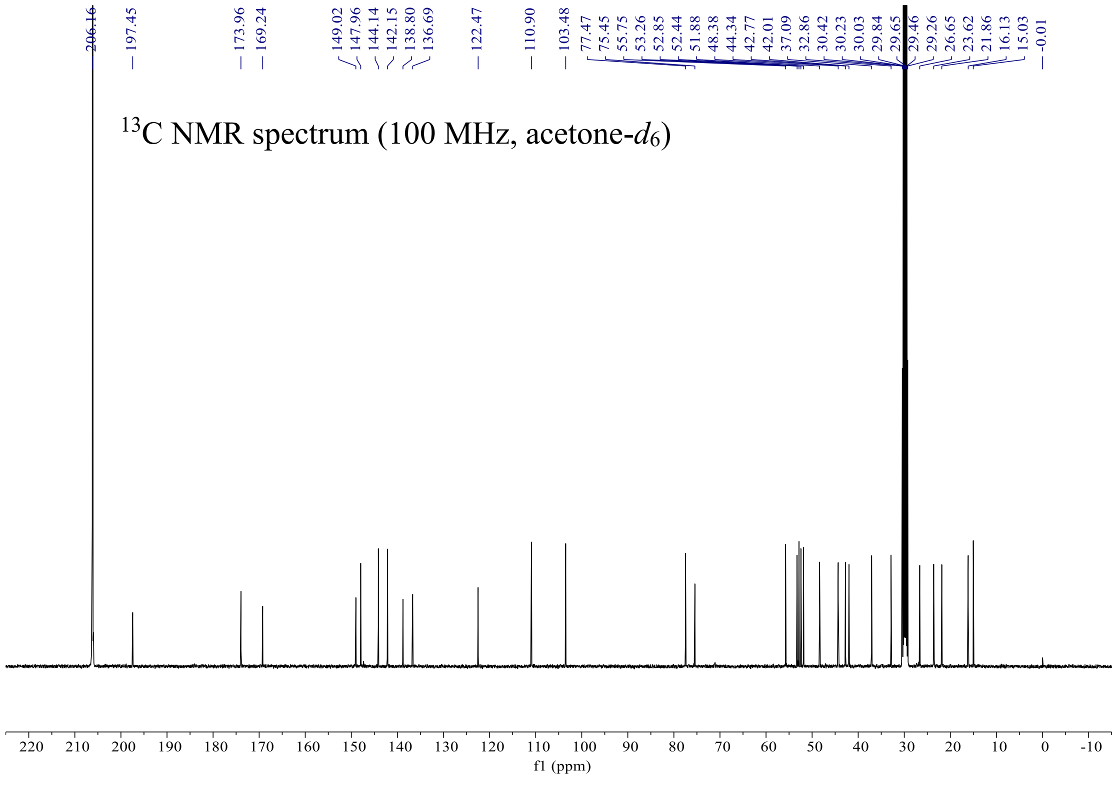


$^{13}$C NMR spectrum (100 MHz, acetone-$d_6$)

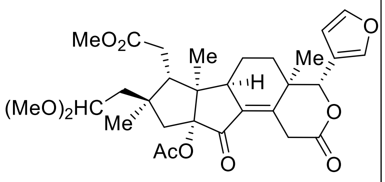
MeO2C
Me
H
Me
O
(MeO)2HC
Me
O
AcO
O
O

**10**

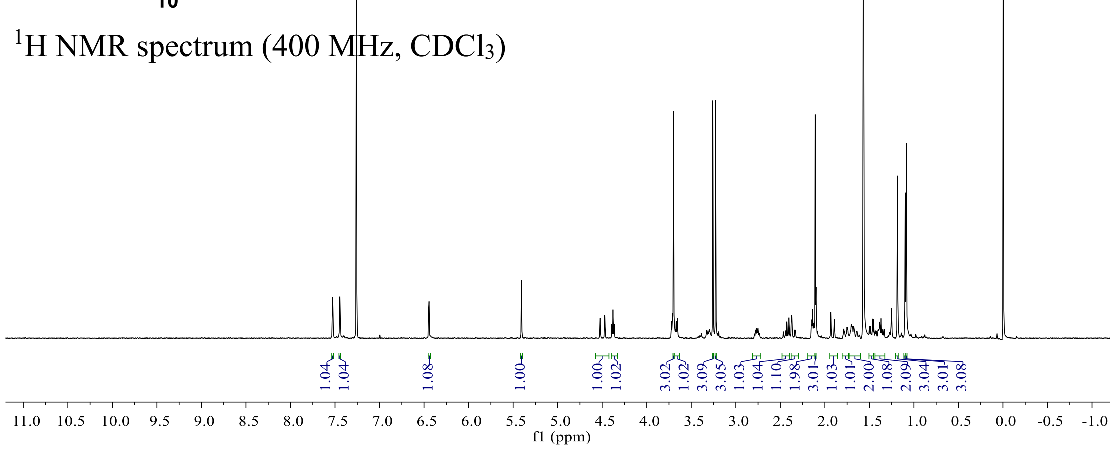
7.5260 7.4500 7.4455 7.4411 7.2600 6.4469 6.4442 5.4068 4.5233 4.4701 4.3920 4.3788 4.3658 3.6993 3.6693 3.6565 3.2577 3.2268 2.7766 2.7634 2.7503 2.4428 2.4262 2.4030 2.3765 2.3705 2.3366 2.3304 2.1512 2.1447 2.1368 2.1277 2.1218 2.1088 1.9320 1.8934 1.7872 1.7780 1.7522 1.7439 1.7129 1.7007 1.6886 1.6753 1.6402 1.4999 1.4874 1.4647 1.4524 1.4353 1.4005 1.3833 1.3692 1.3480 1.3340 1.1845 1.0978 1.0848 -0.0030
1H NMR spectrum (400 MHz, CDCl3)
1.04 1.04 1.08 1.00 1.00 1.02 3.02 1.02 3.09 3.05 1.03 1.04 1.10 1.98 3.01 1.03 1.01 2.00 1.08 2.09 3.04 3.01 3.08
11.0 10.5 10.0 9.5 9.0 8.5 8.0 7.5 7.0 6.5 6.0 5.5 5.0 4.5 4.0 3.5 3.0 2.5 2.0 1.5 1.0 0.5 0.0 -0.5 -1.0
f1 (ppm)

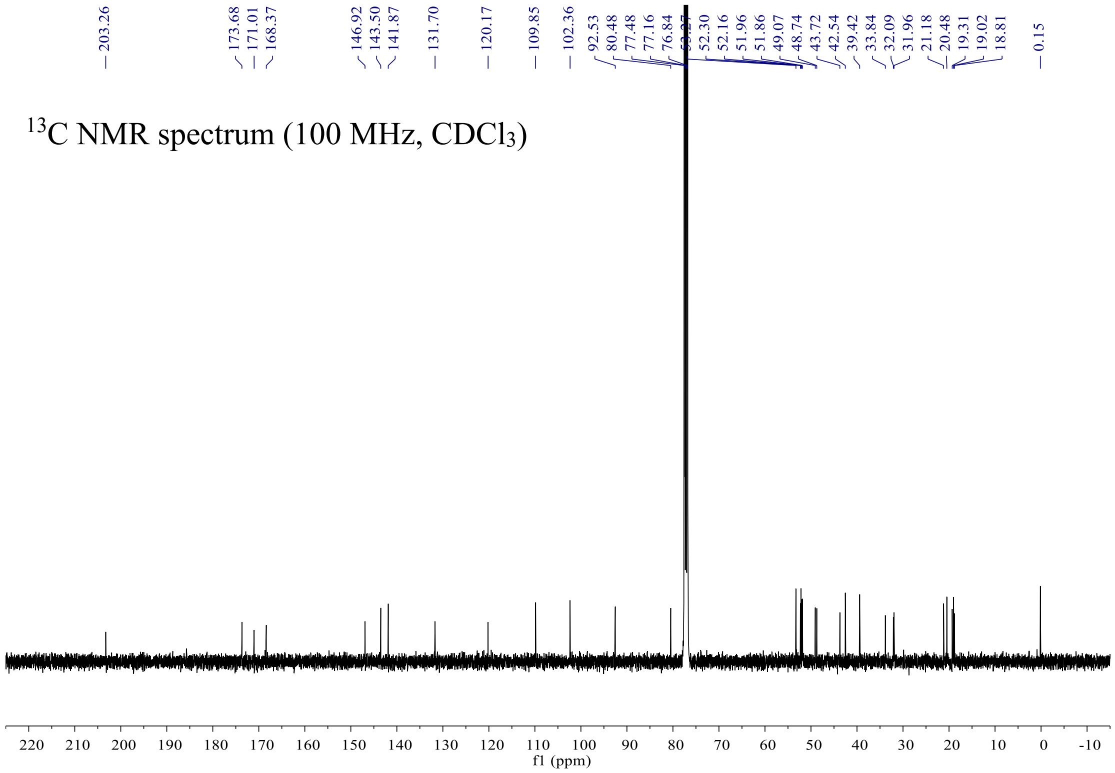
203.26 173.68 171.01 168.37 146.92 143.50 141.87 131.70 120.17 109.85 102.36 92.53 80.48 77.48 77.16 76.84 52.30 52.16 51.96 51.86 49.07 48.74 43.72 42.54 39.42 33.84 32.09 31.96 21.18 20.48 19.31 19.02 18.81 0.15
13C NMR spectrum (100 MHz, CDCl3)
220 210 200 190 180 170 160 150 140 130 120 110 100 90 80 70 60 50 40 30 20 10 0 -10
f1 (ppm)

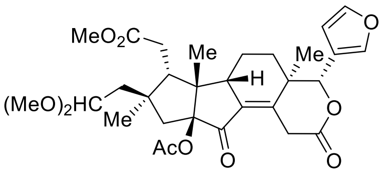


**34**

$^{1}$H NMR spectrum (400 MHz, $CDCl_3$)

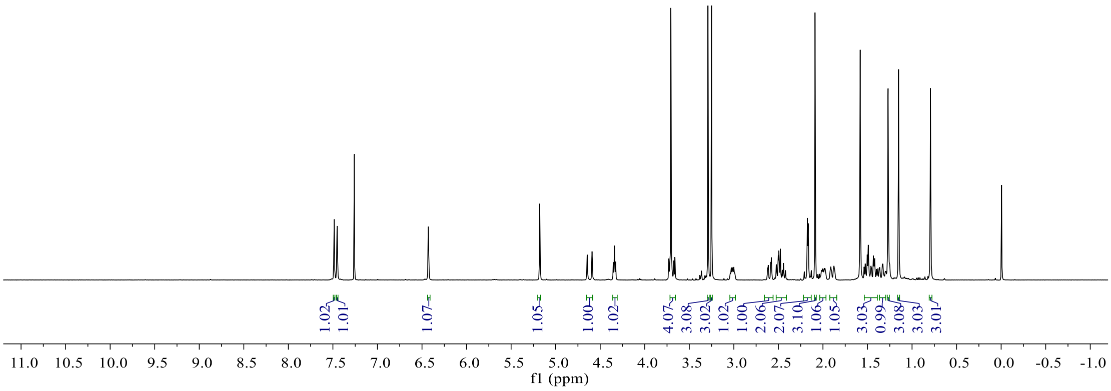


$^{13}$C NMR spectrum (100 MHz, $CDCl_3$)

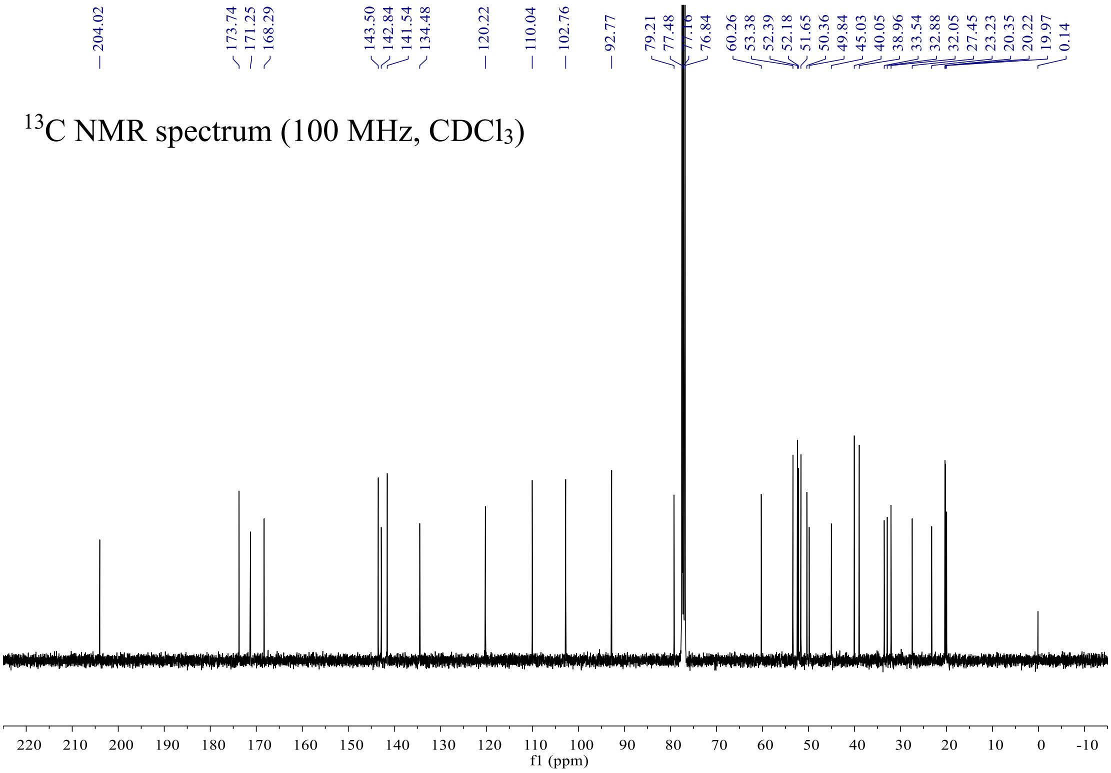

Supplement: File 1 — Experimental procedures, NMR spectra and other characterization data for all new compounds. [file Beilstein_J_Org_Chem-21-926-s001.pdf]
